# Late treatment with autologous expanded regulatory T cell therapy after alemtuzumab induction is safe and facilitates immunosuppression minimization in living donor renal transplantation

**DOI:** 10.1097/TP.0000000000005065

**Published:** 2024-06-07

**Authors:** Matthew O Brook, Conor Hennessy, Salim Hammad, Alaa Alzhrani, Ines Rombach, Susan Dutton, Giovanna Lombardi, Kathryn J Wood, Peter Friend, Joanna Hester, Paul N Harden, Fadi Issa

**Affiliations:** 1 Oxford Transplant Centre, Oxford University Hospitals NHS Foundation Trust, Oxford, UK; 2 Translational Research Immunology Group, University of Oxford, Oxford, UK; 3 Peter Gorer Department of Immunobiology, School of Immunology and Microbial Science, Kings College London; 4 Oxford Clinical Trials Research Unit, Botnar Research Centre, University of Oxford

## Abstract

**Background:**

The TWO study aimed to investigate a novel approach to regulatory T cell (Treg) therapy in renal transplant patients, using a delayed infusion protocol at 6 months post-transplant to promote a Treg-skewed lymphocyte re-population following alemtuzumab induction. We hypothesized that this would allow safe weaning of immunosuppression to tacrolimus alone. The COVID-19 pandemic led to the suspension of alemtuzumab use, and we therefore report the unique seven patient cohort who underwent the original randomized controlled trial protocol. This study presents a unique insight into Treg therapy combined with alemtuzumab, and is therefore an important proof-of-concept for studies in other diseases that are considering lymphodepletion.

**Methods:**

Living donor kidney transplant recipients were randomized to receive autologous polyclonal Tregs at week 26 post transplantation, coupled with weaning doses of tacrolimus, (Treg therapy arm) or standard immunosuppression alone (tacrolimus and mycophenolate mofetil). Primary outcomes were patient survival and rejection free survival.

**Results:**

Successful cell manufacturing and cryopreservation until the 6-month infusion were achieved. Patient and transplant survival was 100%. Acute rejection-free survival was 100% in the Treg treated group at 18 months after transplantation. While alemtuzumab caused a profound depletion of all lymphocytes including Tregs, following cell therapy infusion there was a transient increase in peripheral Treg numbers.

**Conclusion:**

The study establishes that delayed autologous Treg therapy is both feasible and safe, even 12 months post-cell production. The findings present a new treatment protocol for Treg therapy, potentially expanding its applications to other indications.

## 1 Introduction

Solid organ transplant recipients remain dependent on immunosuppressive therapy to prevent rejection of the transplanted organ. Unfortunately, long-term patient and transplant survival are limited by the significant side-effects of immunosuppressive drugs, including increased rates of malignancy, infection and cardiovascular disease^1,2^. Increasing evidence suggests that autologous cell-based therapies may be an effective way of modulating the immune response to allow safe reduction of conventional immunosuppression^3^.

Regulatory T cells (Tregs) are a subset of T lymphocytes that modulate the immune response and prevent an excess inflammatory reaction to alloantigens and are central to maintaining immune homeostasis. Experimental animal models have harnessed this regulatory ability to suppress the inflammatory response and successfully prevent rejection of allografts^4^. Progressive developments in cell culture and manufacture techniques have allowed the ex-vivo expansion of Treg cells from whole blood or leukapheresis products from prospective living donor kidney transplant recipients^5,6^. These cells may be cryopreserved with a negligible impact on their function^7^. Several Phase I clinical trials have reported the feasibility and safety of expanding recipient Treg cells and infusing the cell product safely into the transplant recipient post-transplantation^3,8–11^. Recently, we reported the successful expansion of autologous polyclonal Treg cells and safe infusion into 12 kidney transplant recipients as part of the ONE Study consortium^12^. The Treg-treated cohort had reduced immunosuppression requirements and no acute rejection episodes over 48 months, as well as reduced episodes of opportunistic infection compared to a control cohort who received standard immunosuppression and had an acute rejection rate of 21.1%.

Evidence is now mounting to support the use of Treg therapy to minimize immunosuppression. However, it is not clear which immunosuppression protocol to use or when to infuse cells. Previous work has identified alemtuzumab to promote the enrichment of Treg populations during the late cell repopulation phase at around 6 months post-treatment^13–15^. The 3C Study has also shown alemtuzumab induction to be associated with favourable early rejection rates ^13^. In this study we report a novel approach to the use of autologous ex vivo-expanded naturally-occurring polyclonal Treg therapy in a preliminary randomized controlled study where we have combined alemtuzumab induction with delayed Treg cellular therapy. The infusion of Tregs was delayed with the aim of optimizing the native excess proportion of natural Tregs and transitional B cells that re-populate six months post-transplantation, while providing additional Tregs at the point where they may be needed during post-lymphodepletion cell repopulation. Treg-treated patients further underwent mycophenolate mofetil (MMF) cessation and tacrolimus reduction, with control patients remaining on standard of care immunosuppression with mycophenolate mofetil (MMF) and tacrolimus. The patient cohort described here represent a unique cohort of patients who underwent the original TWO study protocol (supplementary file) (ISRCTN registry 11038572). Due to the COVID-19 pandemic the use of alemtuzumab was suspended nationally in the UK, and the TWO protocol was modified to utilise basiliximab in place of alemtuzumab. The outcomes of the seven patients who completed the alemtuzumab based protocol are reported here and provide and insight into the combination of Treg therapy following alemtuzumab induction. This study therefore provides important proof of concept data for others who may be considering lymphodepletion in conjunction with cellular therapy.

## 2 Materials and Methods

The study was designed as a prospective randomized controlled trial in a single centre in the United Kingdom to explore the role of delayed autologous Treg therapy combined with alemtuzumab induction, MMF cessation, and tacrolimus weaning in living donor kidney transplant recipients ^14^. The objective was to determine the safety and efficacy of delayed Treg therapy during lymphocyte re-population following alemtuzumab induction, as well as the success of Treg treatment at facilitating minimization of immunosuppression. The Treg dose used was based on the ONE Study dose-escalation trial, where 5-10x10^6^ cells/kg was shown to be safe ^12^. Control participants received a standard alemtuzumab-based immunosuppression regimen with long-term tacrolimus and mycophenolate mofetil (MMF) immunosuppression. Primary endpoints were graft survival and the incidence of biopsy-confirmed acute rejection events (Banff criteria) within 18 months of transplantation. The TWO Study is registered on the ISRCTN registry (11038572).

Ethical Approval for the study was granted by the Health Research Authority, South Central, Oxford A research ethics committee (Bristol Research Ethics Committee Centre, Whitefriars, BS1 2NT, UK; +44 (0)207 104 8089; oxforda.rec@hra.nhs.uk), REC ref: 18/SC/0054. The full trial protocol will be made available as a supplementary document.

### 2.1 Patients

**Figure 1:**
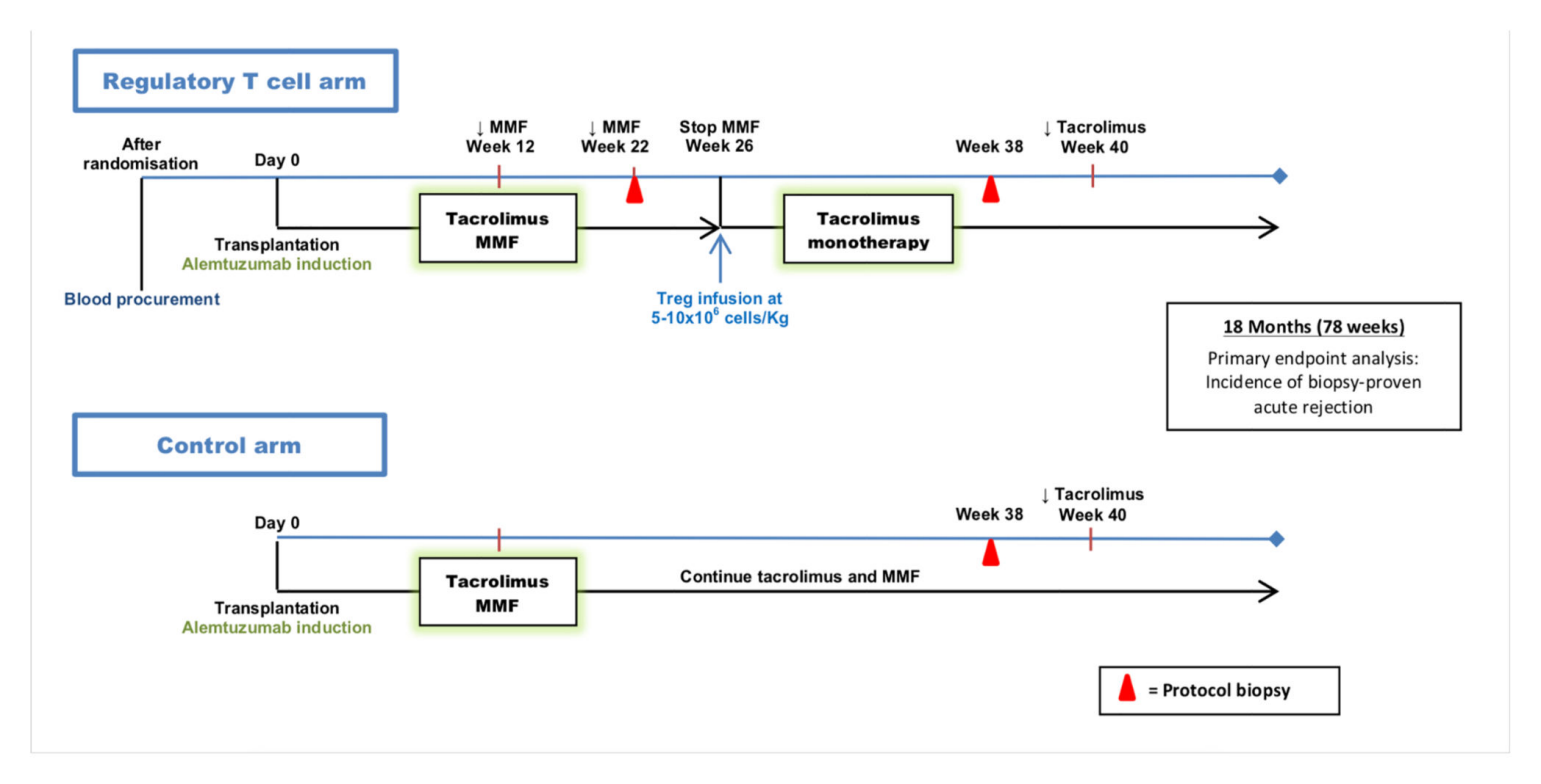
Diagrammatic representation of the original TWO Study trial design. This figure represents the original trial design before Alemtuzumab was discontinued in transplant centres across the UK due to safety concerns. Both arms underwent alemtuzumab induction, and the same initial immunosupressive regimen. At 12 weeks in the cell therapy group, MMF was gradually decreased until week 22, when a protocol biopsy was performed. At week 26 if there were no signs of acute rejection, MMF was stopped, and patients received an infusion of autologous Tregs. Both groups underwent a protocol biopsy at week 38, and follow-up was concluded at 78 weeks (18 months) post-transplantatio

Potential living donor renal transplant recipients were recruited to include first primary transplant recipients and exclude high immunological risk subject (Table 1). Written informed consent was obtained at the pre-visit (V0) prior to eligibility and randomisation, before any trial specific protocols occurred. Information was provided and consent obtained according to the trail protocol (supplementary file). Eligible participants were randomized to the Treg cell therapy arm or standard immunosuppression with alemtuzumab induction and maintenance immunosuppression with tacrolimus and MMF. The cell therapy arm received the same alemtuzumab induction and immunosuppression regimen until week 12 when the MMF dose was progressively reduced until week 22 and a protocol transplant biopsy performed (Figure 1). At week 26 MMF was discontinued, provided the protocol biopsy showed no evidence suggesting acute rejection, and the recipients continued maintenance tacrolimus monotherapy (target trough level 5-10 ng/dl) until the result of a second protocol biopsy at week 38. If the second protocol biopsy revealed no evidence of acute rejection the dose of tacrolimus was reduced (target trough level 4-6ng/dl) until completion of the trial at week 78. The 78-week duration of the trial was determined to capture 90% of primary endpoint events which would have been expected within this timeframe^15^. Recruitment of living donor kidney transplant recipients and donors was carried out in accordance with the inclusion and exclusion criteria outlined in Table 2. As this was designed as a phase II trial, we elected to exclude highly sensitized patients and included only first transplant recipients. Each enrolled patient had 19 trial study visits scheduled relative to the day of transplantation (Day 0). Patient demographics are shown in Table 1. Clinical data collected included creatinine, eGFR, urine protein creatinine ratio and tacrolimus level. Calculation of eGFR was performed using the 2009 CKD-EPI equation.

### 2.2 Manufacture and Treg Therapy

Once randomized, whole blood from each potential living kidney transplant recipient in the Treg therapy arm was collected (370ml), and transported to a GMP manufacturing unit at Guy’s and St Thomas’ NHS Foundation Trust, London UK. Polyclonal Tregs were extracted and expanded in cell culture to a dose of between 5-10x10^6^ cells per kg body weight using a previously validated protocol^6,16^. Briefly, after blood volume reduction, Tregs were isolated via CD8 depletion using CD8 CliniMACS beads, and CD25 enrichment using CD25 CliniMACS beads using CliniMACS Plus Instrument (Miltenyi Biotech). Cells were then stimulated withanti-CD3/anti-CD28 beads (ExpAct Treg kit, Miltenyi Biotec) at a ratio of 4:1 (bead:cell). Rapamycin was added at the beginning of culture and IL-2 added at day 4. Rapamycin and IL2 were replenished every 2-3 days. 3 cycles of bead stimulation were performed. The final product was harvested at day 36. Phenotypic and functional characterisation was performed and then cryopreserved in vapour phase liquid nitrogen ^7^. The cell product was transported in a temperature-monitored cold shipper (-180°C), thawed at the bedside and administered peripherally in 5% human albumin intravenously at week 26, which was 72 hours following cessation of MMF. All clinical trial data up to 78 weeks post-transplant was recorded in a bespoke electronic clinical trial database (Excelya, Germany).

### 2.3 Immune profiling

Immune monitoring was performed using a protocol designed at trial initiation and therefore standardized for the duration of the study. For flow cytometry analysis, 100μl (or 50μl in case of the Treg panel) of EDTA peripheral blood was stained using commercially available pre-mixed, lyophilized antibody panels (Duraclone panels, Beckman Coulter) following the manufacturer’s protocol. For the B cell panel, 300μl of blood was washed twice with PBS before staining. Samples were acquired on a Navios flow cytometer (Beckman Coulter). As a quality control, FlowCheck Pro and FlowSet Pro beads (Beckman Coulter) were run prior to each immunomonitoring visit. Flow cytometry data were analysed using Kaluza software (Beckman Coulter), followed by calculation of absolute cell numbers based on clinical blood morphology results. Data visualisation and statistical analysis were performed using GraphPad Prism 9.

Cytometry of the time of flight (CyTOF) was performed using the MaxPar Direct Immune Profiling Assay (Fluidigm) and acquired at the Mass Cytometry Facility at the Kennedy Institute of Rheumatology on a third-generation Helios mass cytometer (Fluidigm). Briefly, whole blood was incubated with heparin to block Fc receptors, followed by incubation with a pre-mixed cocktail of antibodies. Next, erythrocytes were lysed using ACK Lysing buffer (Gibco), followed by 3 washes with staining buffer and fixation with 1.6% formaldehyde solution. After fixation, cells were cryopreserved in a freezing medium composed of 45% FSC, 45% RPMI, and 10% DMSO and stored in liquid nitrogen for batch analysis. For analysis, samples were thawed, stained with MaxPar Intercalator-Ir (^191^Ir and ^193^Ir) and re-suspended in MaxPar water containing 10% EQ™ four element calibration beads, followed by acquisition on the CyTOF system. FCS files were normalized with calibration beads by Helios software. Manual gating of FCS files was performed using the Cytobank platform. Calibration beads and cell aggregates were excluded through the manual gating, and the population of interest was gated and exported in a new FCS file. Visualization stochastic neighbour embedding algorithm (viSNE) was used for analysis and visualisation of peripheral B cells using the FlowSOM clustering algorithm, heatmaps were performed by the Cytobank platform to show the median expression of individual cellular markers.

## 3 Results

The initial intention was to recruit 68 living kidney donor recipients and donors but recruitment to the trial was halted in March 2020 due to the COVID-19 pandemic. At this stage, 9 recipients had been recruited and we were able to continue to monitor the progress of these patients. Many transplant centres in the UK stopped performing organ transplants for several months during the first wave of the pandemic^17,18^. There was substantial concern about the potential adverse impact of immunosuppression on the severity and risk of COVID-19 infection, in particular profound lymphocyte depletion with alemtuzumab. In response to these concerns we altered the protocol to avoid alemtuzumab induction^14^.

Here we report the results of the 9 living donor kidney transplant recipients in the preliminary phase of the TWO Study prior to its modification, although 2 participants recruited were withdrawn from the trial at an early stage as their transplant was delayed due to the COVID-19 pandemic. The remaining 7 participants have all now completed follow up and form a unique cohort of patients receiving alemtuzumab induction, with 3 receiving delayed Treg cell therapy with immunosuppression minimization and 4 control patients. One of the patients in the cell therapy arm had their protocol biopsy and cell infusion delayed due to the initial lock-down of the COVID pandemic from mid-May 2020 to mid-August 2020.

### 3.1 Clinical Outcomes

**Figure 2:**
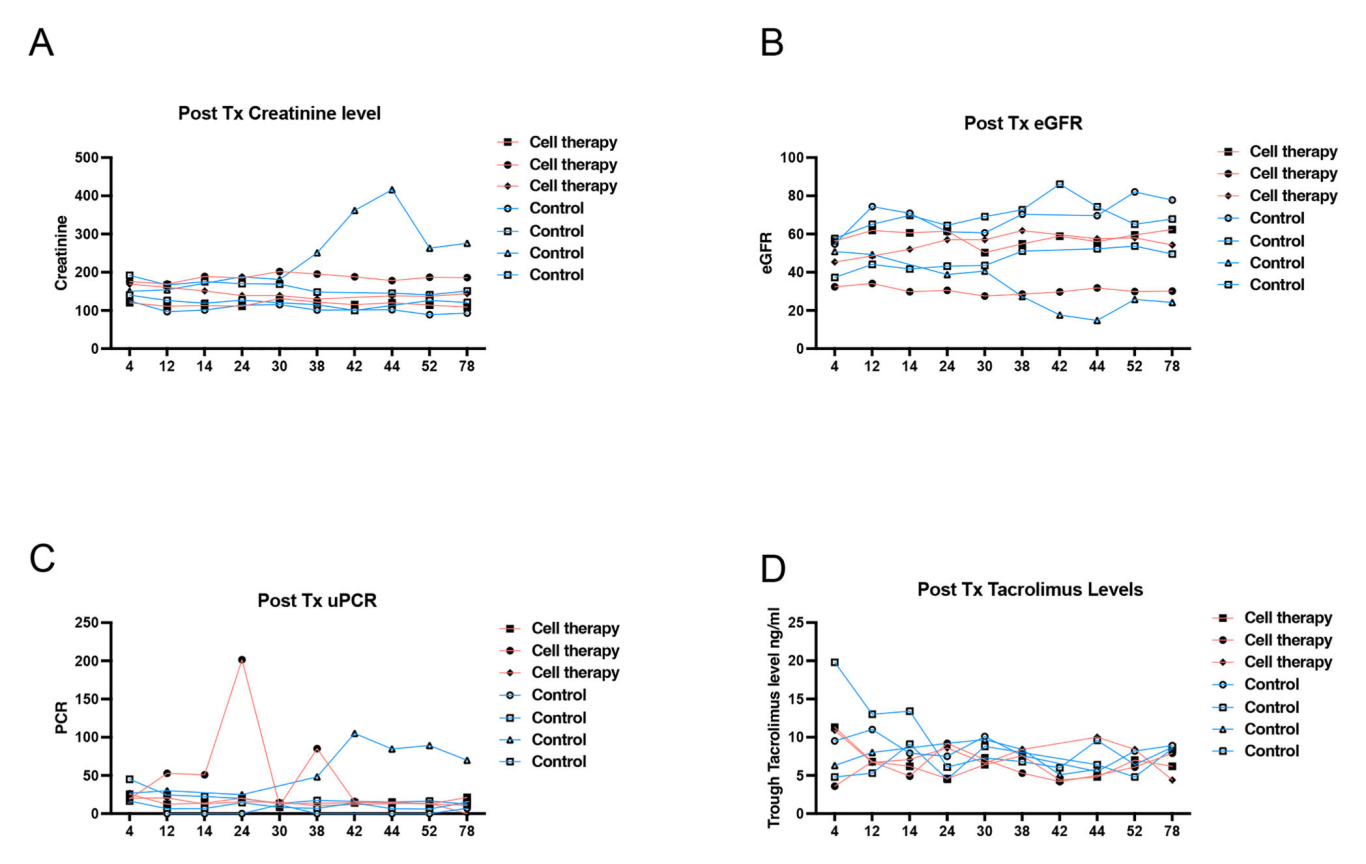
Comparison of creatinine, eGFR, urine protein creatinine ratio and tacrolimus level across follow up points in the Treg treated arm and the control arm. **(A)** Individual measurements of plasma creatinine in the three autologous Treg treated patients and four control patients at 4, 12, 14, 24, 30, 38, 42, 44, 52- and 78-weeks post-transplant. (**B)** Individual measurements of eGFR in the three autologous Treg treated patients and four control patients at 4, 12, 14, 24, 30, 38, 42, 44, 52- and 78-weeks post-transplant. **(C)** Individual measurements of urine protein creatinine ratio in the three autologous Treg treated patients and four control patients at 4, 12, 14, 24, 30, 38, 42, 44, 52- and 78-weeks post-transplant. **(D)** Individual measurements of tacrolimus levels in the three autologous Treg treated patients and four control patients at 4, 12, 14, 24, 30, 38, 42, 44, 52- and 78-weeks post-transplant.

The demographics of the 7 participants are shown in Table 1. Clinical markers of renal function post-transplant are shown in Figure 2. There were no hemodynamic or inflammatory reactions to the infusion of the Treg product and all three patients had the infusion as a day admission and were fit for discharge 4 hours post-infusion. In both groups there was 100% transplant survival at 18 months. No acute rejection episodes occurred in the cell therapy patients, but one of the four control patients had a significant early acute rejection episode in week 1 post-transplant successfully treated with 3 daily doses of methylprednisolone. One of the cell therapy patients and three of the control patients developed neutropenia which responded to temporary cessation of MMF and was thought to be secondary to the combination of alemtuzumab and the trial dose of MMF. Two of these individuals developed mild clinical CMV disease requiring a 3-week course of oral valganciclovir after completion of chemoprophylaxis, one from each treatment arm of the study. Both showed complete resolution of CMV. One control patient developed a decline in transplant function 8 months post-transplant with biopsy evidence of tacrolimus toxicity. Transplant function improved with cessation of tacrolimus and maintenance prednisolone and MMF immunosuppression, however it did not return to baseline. One cell therapy patient had a transient episode of proteinuria, whichspontaneously resolved without explanation. Their kidney function was otherwise stable and protocol biopsies did not show any pathology during the study period. There was no indication to perform a for-cause biopsy. This patient continues to have stable renal graft function. All 7 patients were fit and well at trial completion. In contrast to patients treated with Treg therapy in the ONE Study^12^, there was a notable lack of focal inflammatory infiltrates seen in the protocol biopsies 12 weeks after Treg infusion (Table 3). All 3 cell therapy patients had minimization of immunosuppression to tacrolimus monotherapy and remained free from acute rejection throughout the planned follow up period.

### 3.2 Immunological Outcomes

**Figure 3:**
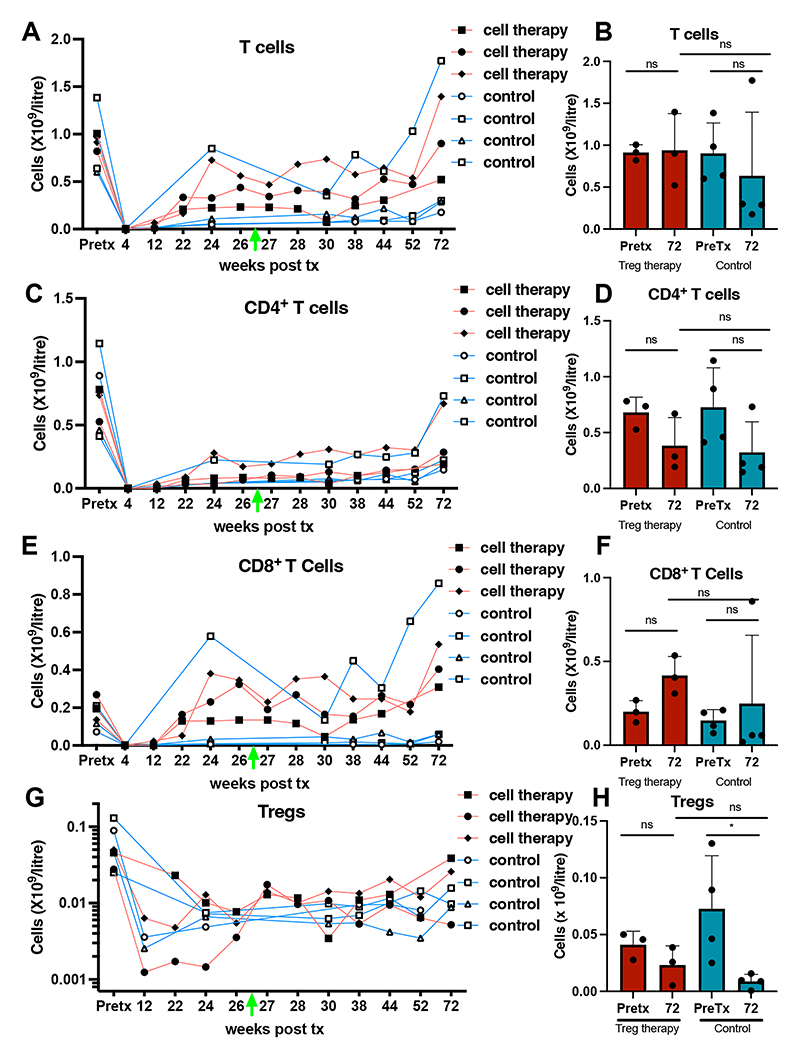
Absolute numbers of T cells, CD4^+^ and CD8^+^ T cells, and FoxP3^+^CD4^+^ Tregs in the peripheral blood over time. **(A-C-E-G)** Absolute numbers of peripheral CD3^+^ T cells, CD4^+^ T cells, and CD8^+^ T cells and FoxP3+CD4+ Tregs in whole blood samples collected from patients prior to transplantation, at 4, 12, 22, 24, 26, 27, 28, 30, 38, 44, 52 and 72 weeks post-transplant (green arrow = week 26, Treg infusion, sample taken prior to cell infusion). Each patient presented as a separate point or point and line, cell therapy patients represented as closed triangles, control patients represented as open squares. **(B-D-F-H)** Absolute numbers of peripheral CD3^+^ T cells, CD4^+^ T cells and CD8^+^ T cells and FoxP3^+^CD4^+^ Tregs in the blood samples of the Treg therapy group (*n* = 3) pre-transplant and at week 72 post-transplant (red) compared to the control group (*n* = 4) pre-transplant and at week 72 (blue). Statistical significance was calculated by one-way ANOVA with Tukey’s for multiple comparisons, ns = not significant, *p>0.05. Data shown as absolute values (A, C, E, G) or mean +/- SD.

As expected, alemtuzumab treatment resulted in a prolonged depletion of T cells, especially CD4^+^ T cells (Figure 3 and Figure S1), with only 2 out of 7 patients returning to a pre-depletion level of CD4^+^ T cells at the 18m visit (week 72). There were no statistically significant differences or obvious trends in levels of total T cells, or CD4^+^ and CD8^+^ T cells and their naïve and memory subsets (Figure 3 and data not shown). Absolute cell numbers of peripheral blood Tregs were reduced after alemtuzumab treatment (Figure 3G), however a relative increase in Treg frequency and the Treg/Teff ratio in the first 12 weeks after induction treatment was observed (Figure S1D). A trend towards a transient increase in Treg numbers was observed at 1 and 2 weeks after cell infusion (Figure 3G and Figure S1D; weeks 27 and 28 post transplantation).

**Figure 4:**
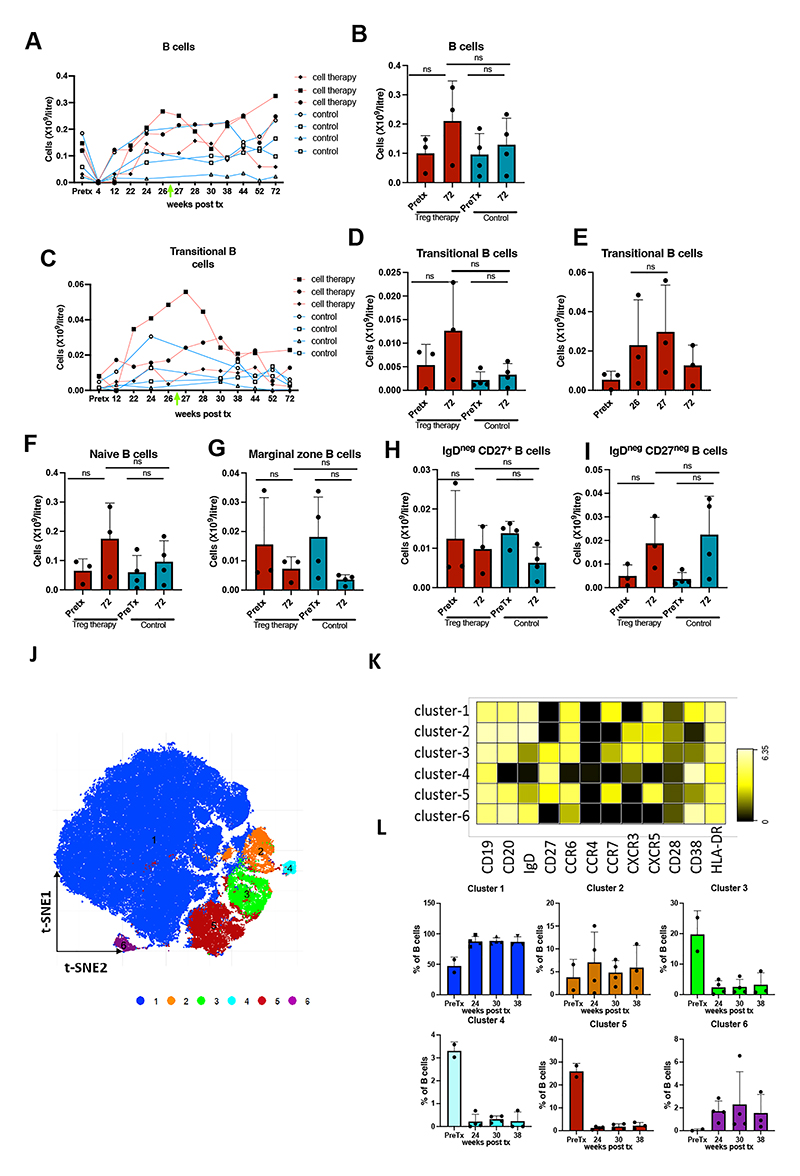
Absolute numbers of B cells in the peripheral blood over time. **(A** and **C)** Absolute numbers of total B cells and CD19^+^CD24^hi^CD38^hi^ transitional B cells in whole blood samples collected from patients prior to transplant, at 4, 12, 22, 24, 26, 27, 28, 30, 38, 44, 52 and 72 weeks post-transplant. Green arrow represents Treg infusion at 26 weeks. Blood samples taken prior to infusion. (**B** and **D)** Absolute numbers of total B cells and CD19^+^CD24^hi^CD38^hi^ transitional B cells in the blood samples of the Treg therapy group (*n* = 3) pre-transplant and at week 72 (red) compared to the control group (*n* = 4) pre-transplant and at week 72 (blue). **(E)** Absolute numbers of CD24^hi^CD38^hi^ transitional B cells in the blood samples of the Treg therapy group (*n* = 3) prior to transplantation and at 26, 27 and 72 weeks post-transplant. **(F-G-H-I)** Absolute numbers of naïve B cells, marginal zone B cells, IgD^neg^CD27^+^ switched memory B cells, and IgD^neg^CD27^neg^ unconventional memory B cells in the blood samples of the Treg therapy group (*n* = 3) prior to transplant and at week 72 (red) compared to the control group (n = 4) pre-transplant and at week 72 (blue). Each patient presented as a separate point or point and line, cell therapy patients represented as closed triangles, control patients represented as open squares. **(J)** Representative viSNE analysis of peripheral B cells using the FlowSOM clustering algorithm from the peripheral blood of renal transplant recipients prior to transplantation (n = 2), at 24 (n = 4), 30 (n = 4), and 38 (n = 4) weeks post-transplant. **(K)** Heatmap showing the median expression of cellular markers expressed in the clusters identified by FlowSOM. Cluster 1 – naïve 1, cluster 2 –naïve 2, cluster 3 – marginal zone B cells 1, cluster 4 – switched memory cells, cluster 5 - marginal zone B cells 2, cluster 6 – transitional B cells. **(L)** Percentages of each cluster within the B cell compartment calculated by the FlowSOM algorithm pre-transplant and at 24, 30, and 38 weeks post-transplant. Dots represent individual samples. Statistical significance was calculated by one-way ANOVA with Tukey’s for multiple comparisons; ns=non-significant. Data shown as absolute values (A, C) or mean +/- SD.

B cells are increasingly recognized as an important component of the immune response to the allograft. Naïve and transitional B cells have been previously reported to be associated with operational tolerance ^19–21^, and we have previously demonstrated an increase in naïve and transitional B cells after alemtuzumab induction^1^. Similar trends can be observed in our limited patient cohort in the Treg-treated group (Figure 4 and Figure S2). Interestingly, in contrast to our previous data from induction free Treg-treated patients from the ONE Study^12^, no increase in marginal zone B cells was observed. The original clinical trial protocol was designed to include donor reactive T cell quantification before and after infusion, however we were unable to obtain these data for the seven patient cohort who underwent the original trial protocol due to challenges with the cell numbers required for these assays.

## 4 Discussion

The TWO Study was designed to test the hypothesis that polyclonal autologous Treg cell infusion would have maximum impact during the lymphocyte re-population phase occurring typically 6 months following infusion of alemtuzumab ^22^. There is evidence that the repopulating lymphocytes are rich in naïve T cells and Tregs with the theoretical potential that infused Tregs could influence the regulatory to effector T cell balance favourably in terms of enhanced natural immune regulation. Here we report the outcome of the patients recruited to the TWO Study original protocol where we demonstrate the feasibility of expansion and cryopreservation of a viable Treg cell product for up to 12 months, as well as the safety of delayed Treg infusion post-transplantation. Unfortunately, the emergence of COVID-19 as a major public health concern resulted in the suspension of alemtuzumab use in transplant centres across the UK. The risk of COVID-19 superinfection following leukodepletion necessitated a switch to alternative induction immunosuppression. The TWO protocol was modified to use basiliximab instead of alemtuzumab, and as such this cohort of 7 patients is a unique subset of patients undergoing this therapeutic combination. This study provides valuable data to support the potential use of Treg therapy in deceased donor kidney transplantation in the future. The only other study to report on this combination of immunotherapy induction and cell therapy is the report by Matthew *et al*, in 2018 ^23^. However, there were some key differences between this study and ours. Firstly, Matthew *et al* infused autologous Tregs at 60 days post alemtuzumab induction, whereas here treatment was delayed for 26 weeks, targeting the lymphocyte repopulation phase. Additionally, Matthew *et al* did not stop MMF treatment, and furthermore converted the tacrolimus to sirolimus. Previous data from our unit has shown conversion to sirolimus to be detrimental^24^ after alemtuzumab induction, and therefore this trial was designed to achieve tacrolimus monotherapy. We achieved this goal in the 3 patients treated with cell therapy without incurring any acute rejection in the period up to 18 months after transplantation. Tacrolimus levels were not significantly different between the control patients and the Treg treated patients. However in the Treg treated patients, the withdrawal of MMF and maintenance on tacrolimus monotherapy represents a significant reduction in immunosuppressive burden. This adds further evidence to the potential value of cell therapy as a means of reducing immunosuppression. An interesting observation was the lack of focal lymphocyte infiltrates in the protocol biopsies, which were prominent in the protocol biopsies following early Treg infusion in the ONE Study ^8,9^. Immune monitoring highlighted similar changes in peripheral immune phenotype as those observed in other Treg trials, with detectable live Tregs post-infusion in a lymphodepleted environment, together with trends towards naïve and transitional B cell increases.

This study suggests that the delayed use of autologous Tregs in living donor kidney transplant recipients, following lymphodepletion with alemtuzumab, is safe, feasible, and allows minimization of immunosuppressive therapy. Furthermore, our analysis shows that there was a transient increase in peripheral Treg numbers following cell therapy, however it was not possible to determine whether this was due to persistence of the infused Tregs, or expansion of endogenous Tregs following the cell therapy. While the trial protocol has since changed, this unique cohort outlines the promising potential for delayed Treg therapy with extended cell cryopreservation, with implications for future studies that may incorporate elements of the protocol.

## Supplementary Material

Supplementary Material

## Figures and Tables

**Table 1:** Demographics of patients included in the trial.

Patient	101	102	103	104	105	106	107
**Treatment group**	Cell therapy	Cell therapy	Control	Control	Cell therapy	Control	Control
**Age**	60	32	57	30	32	35	38
**Sex**	Male	Female	Male	Male	Male	Male	Male
**Race**	White	White	White	White	White	White	White
							
							
**Tx Type**	LRD	LRD	LRD	LRD	LRD	LRD	LURD
**Donor CMV status**	Neg	Pos	Neg	Pos	Neg	Neg	Neg
**Recipient CMV status**	Pos	Pos	Neg	Neg	Neg	Neg	Neg
**Number of mismatches**	0	2	2	2	3	1	2
**cRF %**	34	3	19	0	19	0	33

CMV = cytomegalovirus; LRD = Living related donor; LURD = living unrelated donor; cRF = calculated Reaction Frequency

**Table 2:** Inclusion and exclusion criteria

Donor criteria		Recipient	
Inclusion	Exclusion	Inclusion	Exclusion
Eligible for live donation	Exposure to any investigational agents at or 28 days prior to trial induction	Chronic renal insufficiency necessitating transplantation	Any known contraindication to protocol specific requirements
Age >18	Altruistic donor	Willing and able to give informed consent	ABO blood group incompatibility with donor
ABO blood group compatible with recipient	Paired exchange donor	Age >18	CRF >40% within 6 months prior to transplant
Willing to provide personal, medical and biological data	Any form of substance abuse, psychiatric disorder, or other cause of potential impaired judgement	Can comply with trial requirements	Any form of substance abuse, psychiatric disorder, or other cause of potential impaired judgement
Willing to provide blood samples for analysis		Able to commence immunosuppression at specified time	Concomitant malignancy or history of malignancy within 5 years prior to study entry
Willing and able to give informed consent		Females of CBA/Males with partners of CBA must be willing to use effective contraception for 18 months post trial	Seropositive for HIV, HEPB, HCV, HTLV or syphilis
		Willing to allow his/her GP and/or consultant to be informed of their participation in trial	Significant liver disease (ALT >3x ULN) Participation in another trial within 28 days Female who is pregnant or lactating Any factors which may hamper compliance Any previous desensitisation procedure

**CBA – child bearing age**. **GP – general practitioner (primary care physician)**. **CRF – calculated reaction frequency**

**Table 3:** Results of biopsies obtained from cell therapy and control patients.

Cell therapy				Control arm		
	Patient 1 (105)	Patient 2 (101)	Patient 3 (102)	Patient 1 (104)	Patient 2 (106)	Patient 3 (103)
**Microscopic description**	**10 glomeruli**	**15 glomeruli**	**14 glomeruli**	**16 glomeruli**	**21 glomeruli**	**9 glomeruli**
**Glomerular sclerosis: global**	**neg**	**3/15**	**2/14**	**neg**	**neg**	**neg**
**Glomerular sclerosis: segmental**	**neg**	**n/a**	**n/a**	**neg**	**neg**	**neg**
**Glomerulitis**	**Mild in 3 glomeruli**	**neg**	**neg**	**mild in 1 **	**neg**	**neg**
**Basement membrane**	**normal**	**normal**	**normal**	**normal**	**normal**	**normal**
**Tubulointerstitial fibrosis**	**5%**	**5%**	**10%**	**40%**	**10%**	**0%**
**Tubulitis**	**mild focal lymphocytic**	**none**	**none**	**mild focal lymphocytic**	**none**	**none**
**Vessels**	**normal**	**moderate fibroelastosis**	**mild fibroelastosis**	**normal**	**moderate fibroelastosis**	**normal**
**Rejection**	**no**	**no**	**no**	**no**	**no**	**no**
**C4d staining**	**neg**	**neg**	**neg**	**neg**	**neg**	**neg**
**SV40 staining**	**neg**	**neg**	**neg**	**neg**	**neg**	**neg**
**Comment**	**Minimal abnormality** **No evidence of rejection**	**Minimal chronic damage. No evidence of rejection**	**Mild acute tubular injury. Mild chronic damage**	**Moderate chronic damage. Extensive mononuclear infiltrate in areas of fibrosis**	**Mononuclear infiltrate in areas of fibrosis**	**Minimal abnormality. No evidence of rejection**

All three of the cell therapy group had protocol biopsies at 9 months. Three of the control patients had biopsies as one declined. SV40 = simian virus 40. C4d = complement split product, marker of antibody mediated rejection in transplant.

## Data Availability

The authors can be contacted for queries and any additional data.

## Abbreviations

### Abbreviations

CyTOF: Cytometry of the time of flight
DMSO: Dimethyl sulfoxide
EDTA: Ethylenediaminetetraacetic acid
FCS: Flow Cytometry Standard
MMF: mycophenolate mofetil
PBS: Phosphate-buffered saline
Treg: Regulatory T cells
TWO: transplantation without overimmunosuppression
viSNE: Visualization stochastic neighbour embedding algorithm
