# Supplementary material for: Late treatment with autologous expanded regulatory T cell therapy after alemtuzumab induction is safe and facilitates immunosuppression minimization in living donor renal transplantation
Source: Transplantation. Author manuscript; Available in PMC 2024 Sep 19. (PMC7616465; doi:10.1097/TP.0000000000005065)
Supplement: Supplementary Material [file EMS195686-supplement-Supplementary_Material.pdf]

The TWO Study: Transplantation Without Over-immunosuppression

A Phase IIb Trial of Regulatory T Cells in Renal Transplantation

**Internal Reference Number / Short title:**	TWO Study: Treg Cell Therapy Trial
**Ethics Ref:**	18/SC/0054
**IRAS Project ID:**	227287
**EudraCT Number**:	2017-001421-41
**ISRCTN Number:**	11038572
**Date and Version No**:	18Mar2019 v5.0

Chief Investigator:

Prof. Peter Friend
Oxford Transplant Centre
Oxford University Hospitals NHS Foundation Trust
Churchill Hospital
Oxford
OX3 7LE
Tel +44 (0) 1865 226109
peter.friend@ouh.nhs.uk

Investigators:

Dr. Paul Harden (Clinical PI, OUH)
Oxford Transplant Centre, Oxford University Hospitals NHS Trust
paul.harden@ouh.nhs.uk

Dr. Fadi Issa (Scientific PI, University of Oxford)
Transplantation Research Immunology Group
Nuffield Dept. of Surgical Sciences, University of Oxford
fadi.issa@nds.ox.ac.uk

Co-Investigators:

Ms. Susan Dutton
Oxford Clinical Trials Research Unit
Botnar Research Centre, University of Oxford

Dr. Joanna Hester
Transplantation Research Immunology Group
Nuffield Dept. of Surgical Sciences, University of Oxford

Prof. Giovanna Lombardi
Immunoregulation Laboratory, MRC Centre for Transplantation
Guy's Hospital, London

© Copyright: The University of Oxford and Oxford University Hospitals NHS Foundation Trust 2016

Dr. Matthew Brook
Transplantation Research Immunology Group
Nuffield Dept. of Surgical Sciences, University of Oxford

Prof. Kathryn Wood
Transplantation Research Immunology Group
Nuffield Dept. of Surgical Sciences, University of Oxford

Sponsor: University of Oxford
Wellington Square
Oxford
OX1 2JD
United Kingdom
Telephone: +44 (0) 1865 270000
Fax: +44 (0) 1865 270708

Funder: Medical Research Council
14th floor
One Kemble Street
London
WC2B 4AN
Telephone: 01793 416200

Chief Investigator Signature: ______________________

Statistician Signature: ______________________

There are no potential conflicts of interest to declare.

Confidentiality Statement

This document contains confidential information that must not be disclosed to anyone other than the Sponsor, the Investigator Team, HRA, host organisation, and members of the Research Ethics Committee, unless authorised to do so.

© Copyright: The University of Oxford and Oxford University Hospitals NHS Foundation Trust 2016

TABLE OF CONTENTS

1. KEY TRIAL CONTACTS 9
2. SYNOPSIS 12
3. ABBREVIATIONS 14
4. BACKGROUND AND RATIONALE 19
4.1. Study Rationale and Concept 19
4.2. Organ Transplantation 19
4.3. Kidney Transplantation and Immunosuppression 19
4.4. Immunosuppression Minimisation 20
4.5. Immunological Tolerance of Transplants 21
4.6. Cell-Based Immunosuppression 21
4.7. Human Regulatory T Cells (Tregs) 21
4.8. Pre-clinical Studies of Treg Cellular Therapy 21
4.9. Previous Clinical Studies of Treg Cellular Therapy 22
4.10. Clinical Treg Therapy in Renal Transplant Recipients 22
5. OBJECTIVES AND OUTCOME MEASURES 23
5.1. Primary Objective 23
5.2. Primary Outcome Measure 23
5.3. Time-Point of Evaluation of Primary Outcome Measure 24
5.4. Secondary Objectives 24
5.5. Secondary Outcome Measures 25
5.6. Time-point of Evaluation of Secondary Outcome Measures 25
5.7. Exploratory Objectives and Outcome Measures 26
5.8. Time-point of Evaluation of Exploratory Outcome Measures 26
6. TRIAL DESIGN 26
6.1. Trial Duration 26
Figure 1. Cumulative incidence of first rejection episodes in first three-years post-transplant 27
6.2. Definition of Trial Time Measurements 27
6.3. Trial Follow-Up 27
7. Trial Participants 28
7.1. Trial Participants 28
7.2. Kidney Donor Inclusion Criteria 28

© Copyright: The University of Oxford and Oxford University Hospitals NHS Foundation Trust 2016

7.3. Kidney Recipient Inclusion Criteria ... 28
7.4. Kidney Donor Exclusion Criteria ... 29
7.5. Kidney Recipient Exclusion Criteria ... 29
7.6. Contraception ... 30
8. TRIAL PROCEDURES ... 31
8.1. Initial Contact and Screening ... 31
8.2. Informed Consent ... 31
8.3. Randomisation ... 32
9. Donor Study Visits ... 32
9.1. Visit 0 (Pre-visit) ... 32
9.2. Visit 2 (both arms) ... 32
9.3. Day 0 - Operation (Both arms) ... 33
10. Recipient Study Visits ... 33
10.1. Pre-visit (Visit 0) ... 33
10.2. Visit 1 – Treg arm only ... 34
10.3. Visit 2 – Both arms ... 34
10.4. Day 0 (Operation, standard care – both arms) ... 35
10.5. Visit 3 (both arms) ... 35
10.6. Visit 4 (both arms) ... 36
10.7. Visit 5 (both arms) ... 36
10.8. Visit 6 (both arms) ... 36
10.9. Visit 7 (Biopsy - Treg arm only) ... 36
10.10. Visit 8 (both arms) ... 37
10.11. Visit 9 (Treg arm only) ... 37
10.12. Visits 10 and 11 (Treg arm only) ... 39
10.13. Visit 12 (both arms) ... 39
10.14. Visit 13 (both arms) ... 39
10.15. Visit 14 (both arms) ... 39
10.16. Visit 15 (Treg arm only) ... 40
10.17. Visit 16 (both arms) ... 40
10.18. Visits 17-19 (both arms) ... 40
10.19. Visits 20-23 (both arms) ... 40
10.20. Unscheduled visits ... 40
10.21. Trial Flow-Chart ... 42

© Copyright: The University of Oxford and Oxford University Hospitals NHS Foundation Trust 2016

Table 1. Schedule of all recipient trial visits in the TWO Study: Treg Cell Therapy Trial 42
Table 2. Schedule of recipient clinical assessments 45
11. Sample Handling 46
12. Measurement of Glomerular Filtration Rate and Integrity 46
13. Kidney Graft Biopsies 47
13.1. For-cause biopsies 47
13.2. Biopsy material 47
13.3. Severity of rejection episodes 48
14. Immune Monitoring (IM) 48
14.1. Additional Blood Collection Time Points 49
14.2. Objectives of IM 49
14.3. Design of the IM component of the study 49
14.4. Measures of Safety 49
14.5. Potential immunological indicators of rejection/unresponsiveness 50
14.6. Sampling for immune monitoring assays 51
Table 3. Immune monitoring assays 52
Table 4. Sampling and tubes for immune monitoring assays 53
15. Discontinuation/Withdrawal of Participants from Trial Treatment 53
15.1. Patient Replacement 54
Figure 2. Diagram of potential scenarios that may lead to patient replacement. 55
15.2. Definition of End of Trial 56
16. TRIAL MEDICATION 56
16.1. Drug Regimen (NIMPs) 56
16.2. Monitoring of immunosuppression 57
16.3. Corticosteroids 57
16.4. Alemtuzumab 57
16.5. Tacrolimus 58
16.6. Mycophenolate Mofetil (MMF) 58
Table 5 MMF dosing by treatment arm and time point 58
16.7. Paracetamol 59
16.8. Chlorpheniramine 59
16.9. Supply of ATIMP (See also section 10.11 above) 59
16.10. Labelling of ATIMP 59
Figure 4. Sample label for TR001 cell product 59

© Copyright: The University of Oxford and Oxford University Hospitals NHS Foundation Trust 2016

16.11. Storage of ATIMP ... 60
16.12. Dosage and administration of the ATIMP ... 60
16.13. Accountability of the ATIMP ... 60
16.14. Compliance with Trial Treatment ... 61
16.15. Non-compliance with the immunosuppressive therapy ... 61
16.16. Grade 1 Protocol Treatment Non-Compliance ... 61
16.17. Grade 2 Protocol Treatment Non-Compliance ... 62
16.18. Accountability of the Trial Treatment ... 62
16.19. Concomitant Medication ... 62
16.20. Post-Trial Treatment ... 63
17. SAFETY REPORTING ... 63
17.1. Definitions ... 63
17.2. Causality ... 64
17.3. Adverse Events (AE) ... 64
17.4. Reporting Procedures for Serious Adverse Events ... 65
17.5. Expectedness ... 65
Alemtuzumab ... 65
TR001 Cell Therapy ... 65
Tacrolimus ... 66
Mycophenolate Mofetil ... 66
Methylprednisolone ... 66
Paracetamol ... 66
Chlorpheniramine ... 66
17.6. SUSAR Reporting ... 66
17.7. Data and Safety Monitoring Committee ... 66
17.8. Development Safety Update Reports ... 67
18. STATISTICS ... 67
18.1. Description of Statistical Methods ... 67
18.2. The Number of Participants ... 68
18.3. The Level of Statistical Significance ... 68
18.4. Criteria for the Termination of the Trial ... 68
Table 6. Recruitment targets by time from study initiation ... 69
18.5. Procedure for Accounting for Missing, Unused, and Spurious Data. ... 69
18.6. Procedures for Reporting any Deviation(s) from the Original Statistical Plan ... 69

© Copyright: The University of Oxford and Oxford University Hospitals NHS Foundation Trust 2016

19. DATA MANAGEMENT 70
19.1. Source Data 70
19.2. Access to Data 70
19.3. Data Recording and Record Keeping 70
20. QUALITY ASSURANCE PROCEDURES 71
20.1. Monitoring 71
20.2. Data & Safety Monitoring Committee (DSMC) 71
20.3. Trial Steering Committee (TSC) 71
20.4. Trial Management Group (TMG) 71
20.5. Project Management Group (PMG) 72
20.6. Serious Breaches 72
21. ETHICAL AND REGULATORY CONSIDERATIONS 72
21.1. Declaration of Helsinki 72
21.2. Guidelines for Good Clinical Practice 72
21.3. Approvals 73
21.4. Reporting 73
21.5. Participant Confidentiality 73
21.6. Expenses and Benefits 73
21.7. Other Ethical Considerations 73
22. FINANCE AND INSURANCE 73
22.1. Funding 74
22.2. Insurance 74
23. PUBLICATION POLICY 74
24. REFERENCES 75
25. APPENDIX A: AMENDMENT HISTORY 81

© Copyright: The University of Oxford and Oxford University Hospitals NHS Foundation Trust 2016

TABLES AND FIGURES

Table 1. Schedule of all trial visits in the TWO Study: Treg Cell Therapy Trial 42
Table 2. Schedule of clinical assessments 45
Table 3. Immune monitoring assays 52
Table 4. Sampling and tubes for immune monitoring assays 53
Table 5 MMF dosing by treatment arm and time point 58
Table 6. Recruitment targets by time from study initiation 69
Figure 1. Cumulative incidence of first rejection episodes in first three-years post-transplant 27

Figure 2. Diagram of potential scenarios that may lead to patient replacement. 55
Figure 3. Diagrammatic representation of the immunosuppressive regimen for kidney transplant recipients in the TWO Study .... **Error! Bookmark not defined.**
Figure 4. Sample label for TR001 cell product 59

© Copyright: The University of Oxford and Oxford University Hospitals NHS Foundation Trust 2016

1. KEY TRIAL CONTACTS

**Chief Investigator**	Prof. Peter Friend<br>Oxford Transplant Centre<br>Oxford University Hospitals NHS Foundation Trust<br>Churchill Hospital<br>Oxford<br>OX3 7LE<br>Telephone: +44 (0) 1865 226109<br>Peter.friend@ouh.nhs.uk
**Clinical PI**	Dr. Paul Harden<br>Oxford Transplant Centre<br>Oxford University Hospitals NHS Trust<br>Churchill Hospital<br>Oxford<br>OX3 7LE<br>Telephone: +44 (0) 1865 225614<br>paul.harden@ouh.nhs.uk
**Scientific PI**	Dr. Fadi Issa<br>Transplantation Research Immunology Group<br>Nuffield Department of Surgical Sciences<br>University of Oxford<br>Telephone: + 44 (0)1865 222508/221310<br>fadi.issa@nds.ox.ac.uk
**Immune Monitoring Lead**	Dr. Joanna Hester<br>Transplantation Research Immunology Group<br>Nuffield Dept. of Surgical Sciences<br>University of Oxford<br>Telephone: + 44 (0)1865 222508/221310<br>joanna.hester@nds.ox.ac.uk
**Sponsor**	University of Oxford<br>University Offices<br>Wellington Square<br>Oxford<br>OX1 2JD<br>United Kingdom<br>Telephone: +44 (0) 1865 270000<br>Fax: +44 (0) 1865 270708
**Clinical Trials Unit**	Surgical Intervention Trials Unit<br>Nuffield Department of Surgical Sciences<br>Botnar Research Centre<br>University of Oxford<br>Nuffield Orthopaedic Centre<br>Windmill Road<br>OXFORD<br>OX3 7HE<br>Telephone: +44 (0) 1865 223491<br>situ@nds.ox.ac.uk
**Good Manufacturing**	Dr. Laura Fry (Quality Manager)

© Copyright: The University of Oxford and Oxford University Hospitals NHS Foundation Trust 2016

**Practice Unit**	Biomedical Research Centre<br>Guy's Hospital<br>London<br>SE1 9RT<br>+44 (0)20 7188 7188 ext. 52703<br>laura.fry@gstt.nhs.uk
**Pathologist**	Prof. Ian Roberts<br>Department of Cellular Pathology<br>John Radcliffe Hospital<br>Headington<br>Oxford<br>OX3 9DU<br>Telephone: +44 (0) 1865 220490 / 91<br>Fax: +44 (0) 1865 220516<br>ian.roberts@ouh.nhs.uk
**Statistician**	Ms. Susan Dutton<br>Oxford Clinical Trials Research Unit<br>Botnar Research Centre, University of Oxford<br>Nuffield Orthopaedic Centre<br>Windmill Road<br>Oxford<br>OX3 7HE<br>Telephone: +44 (0) 1865 223451<br>susan.dutton@csm.ox.ac.uk<br><br>Ms Ines Rombach<br>Oxford Clinical Trials Research Unit<br>Botnar Research Centre, University of Oxford<br>Nuffield Orthopaedic Centre<br>Windmill Road<br>Oxford<br>OX3 7HE<br>Telephone: +44 (0) 1865 223451<br>Ines.rombach@ndorms.ox.ac.uk
**Data and Safety Monitoring Committee (DSMC)**	Prof. Alan Salama (**Chair**)<br>UCL Centre for Nephrology<br>Royal Free Hospital<br>Rowland Hill Street<br>London NW3 2PF<br>UK<br>Telephone: +44 (0)20 7794 0500 Ext. 36007<br>a.salama@ucl.ac.uk<br><br>**Members**:<br>Dr. Paul Harden (in attendance in open session; Oxford Transplant Centre)<br>Miss Lorna Marson (University of Edinburgh)<br>Dr. Nicholas Torpey (Cambridge Transplant Centre)<br>Mr Chen Ji (Independent statistician; University of Warwick)<br>Ms. Monica Dolton (as observer; University of Oxford)

© Copyright: The University of Oxford and Oxford University Hospitals NHS Foundation Trust 2016

**Trial Steering Committee (TSC)**	Prof. Edward Geissler (**Chair**)<br>Dept. of Surgery<br>University Hospital Regensburg<br>Franz-Josef-Strauss-Allee 11<br>93053 Regensburg<br>Germany<br>Telephone: +49 941 944 6964 or 6961<br>Fax: +49 941 944 6886<br>edward.Geissler@klinik.uni-regensburg.de<br><br><br>**Members**:<br>MRC representative as observer<br>Dr. Sophie Brouard (Université de Nantes)<br>Ms. Sue Dutton (University of Oxford)<br>Prof. Peter Friend (Oxford Transplant Centre)<br>Prof. John Isaacs (University of Newcastle)<br>Dr. Fadi Issa (University of Oxford)<br>Dr Paul Harden (Oxford Transplant Centre)<br>Dr. Qizhi Tang (University of California)<br>Dr. Jason Lowe (patient representative)
**Trial Management Group (TMG)**	Dr. Joanna Black<br>Dr. Matthew Brook<br>Ms. Sarah Deakin<br>Ms. Monica Dolton<br>Ms. Sue Dutton<br>Dr. Paul Harden<br>Dr. Joanna Hester<br>Dr. Fadi Issa<br>Mr. Damian Haywood<br>Prof. Kathryn Wood<br>Dr. Ines Rombach
**Project Management Group (PMG)**	Ms. Monica Dolton<br>Dr. Paul Harden<br>Dr. Joanna Hester<br>Dr. Fadi Issa<br>Prof. Kathryn Wood

© Copyright: The University of Oxford and Oxford University Hospitals NHS Foundation Trust 2016

2. SYNOPSIS

**Trial Title**	The TWO Study: Transplantation Without Over-Immunosuppression. A Phase IIb Trial of Regulatory T Cells in Renal Transplantation.
**Short title**	TWO Study: Treg Cell Therapy Trial
**Clinical Phase**	Phase IIb
**Trial Design**	Non-commercial, investigator-initiated, single centre randomised-controlled phase II trial
**Trial Participants**	Kidney transplant recipients and their matched donors
**Planned Sample Size**	68 kidney transplant recipients and up to 68 paired living kidney donors (1:1 randomisation). The kidney donors are recruited for generation of baseline immunological and clinical reference data
**Treatment duration**	One Treg cell infusion at 6-months (week 26) post-transplant<br>Current immunosuppression standard of care for all groups with a trial duration of 5 years.
**Follow up duration**	5 years from time of transplant (week 260)
**Planned Trial Period**	98 months

	Objectives	Outcome Measures
**Primary**	To compare the efficacy of autologous regulatory T cell therapy used in conjunction with tacrolimus to standard of care immunosuppression (MMF and tacrolimus) in preventing acute biopsy-proven rejection in renal transplant recipients.	Incidence of biopsy-confirmed acute rejection between 6 and 18 months following renal transplant.
**Secondary**	Secondary endpoints will be used to assess the safety, feasibility and potential additive benefits of both cellular therapy and the associated immunosuppression minimisation. Secondary objectives will therefore be assessed as:<br>1. Indicators of influence of Treg administration on graft outcome<br>2. Markers of over-suppression of the immune system<br>3. Signs of chronic toxicity associated with infusion of cell products<br>4. Patient quality of life	Indicators of influence of Treg administration on graft outcomes will include:<br><br>Impact on acute rejection<br>• Time to first acute rejection episode<br>• Severity of acute rejection episode based on response to treatment and histological scoring<br>• Total immunosuppressive burden at the final trial visit<br>• Incidence of graft loss through rejection<br><br>Success in reduction of immunosuppression<br>• Proportion of patients on tacrolimus monotherapy at the end of the study<br><br>Prevention of chronic graft dysfunction<br>• Chronic allograft dysfunction or interstitial fibrosis/tubular atrophy (IF/TA) assessed by clinical (impairment of eGFR) and

© Copyright: The University of Oxford and Oxford University Hospitals NHS Foundation Trust 2016

		histopathological (Banff staging) measures<br><br>Avoidance of drug-related complications by immunosuppressant reduction<br>• Incidence of drug-related adverse events<br><br>Patient survival<br><br>Markers of over-suppression of the immune system will include:<br>• Incidence of serious and/or opportunistic infections (especially CMV, EBV and polyoma (BK) virus)<br>• Incidence of neoplasia<br><br>Signs of chronic toxicity associated with infusion of cell products will include:<br>• Incidence of auto-immune disorders<br>• Incidence of anaemia, cytopaenias, or biochemical disturbances unrelated to the function of the transplanted kidney<br><br>Patient quality of life will be assessed using SF-36 and EQ-5L-5D questionnaires.
**Exploratory**	The use of a number of immune monitoring techniques to predict tolerance and rejection, and detect an effect of Treg therapy<br><br>Assessment of viral infections within transplant recipients	• Flow cytometric cell phenotype analysis using standardised immune monitoring panels<br>• T cell functional assays against donor and third-party antigen including CD154/CD137 ratio analysis<br>• Serum cytokine analysis and metabolic profiling<br>• Gene expression and RNA sequencing analyses including a Treg-Specific Demethylated Region (TSDR) analysis and T-Cell Receptor Sequencing<br>• Analysis of immune infiltrate within transplant biopsy samples for cell phenotype and transcript<br>• Next generation sequencing of viruses when detected in clinical specimens.

© Copyright: The University of Oxford and Oxford University Hospitals NHS Foundation Trust 2016

**Investigational Medicinal Product(s)**	Autologous polyclonal naturally-occurring regulatory T cells ('TR001')
**Formulation, Dose, Administration Route**	Purified Treg cells suspended in 4.5-5% human albumin solution, $10x10^6$ cells/kg body weight, administered intravenously
**Non-Investigational Medicinal Products**	Alemtuzumab; methylprednisolone; paracetamol; tacrolimus; mycophenolate mofetil; Chlorpheniramine

3. ABBREVIATIONS

Ab	Antibody
ABMR	Antibody-Mediated Rejection
ACE	Angiotensin-Converting Enzyme
AE	Adverse Event
Ag	Antigen
ALT	Alanine Transaminase
AML	Acute Myeloid Leukaemia
APC	Antigen Presenting Cell
APTT	Activated Partial Thromboplastin Time
AR	Adverse Reaction
ATG	Anti-Thymocyte Globulin
ATIMP	Advanced Therapy Investigational Medicinal Product
BCAR	Biopsy-Confirmed Acute Rejection
BD	Bis Dia (Twice Daily)
BKV	BK Virus
BMI	Body Mass Index
BTS	British Transplant Society
BW	Body Weight
CAN	Chronic Allograft Nephropathy
CD-	Cluster Of Differentiation
CF	Consent Form
CI	Chief Investigator
CKD	Chronic Kidney Disease
CKD-EPI	Chronic Kidney Disease Epidemiology Collaboration
CML	Chronic Myeloid Leukaemia

© Copyright: The University of Oxford and Oxford University Hospitals NHS Foundation Trust 2016

CMV	Cytomegalovirus
CNI	Calcineurin Inhibitor
CRA	Clinical Research Associate (Monitor)
CrCl	Creatinine Clearance
CRF	Case Report Form
cRF	Calculated Reaction Frequency
CRO	Contract Research Organisation
CRP	C-Reactive Protein
CT	Clinical Trials
CTA	Clinical Trials Authorisation
CTRG	Clinical Trials And Research Governance
CVC	Central Venous Catheter
CyTOF	Mass Cytometry
DMC/DMSC	Data Monitoring Committee / Data Monitoring And Safety Committee
DSA	Donor Specific Antibody
DSMC	Data Safety Monitoring Board
DST	Donor Specific Transfusion
DSUR	Development Safety Update Report
EBV	Epstein-Barr Virus
ECG	Electrocardiogram
eGFR	Estimated Glomerular Filtration Rate
ELISA	Enzyme-Linked Immunosorbant Assay
ELISPOT	Enzyme-Linked Immunosorbant Spot
ESKD	End-Stage Kidney Disease
FBC	Full Blood Count
FOXP3	Forkhead Box P3
FTE	Full Time Equivalent
G&S	Group & Save Sample
GCP	Good Clinical Practice
GFR	Glomerular Filtration Rate
GMP	Good Manufacturing Practice
GP	General Practitioner
GSTT	Guy's and St. Thomas' Hospitals NHS Foundation Trust
GTAC	Gene Therapy Advisory Committee

© Copyright: The University of Oxford and Oxford University Hospitals NHS Foundation Trust 2016

GvHD	Graft Versus Host Disease
Hb	Haemoglobin
HBV	Hepatitis B Virus
HCV	Hepatitis C Virus
HERC	Health Economics Research Centre
HHV-8	Human Herpes Virus-8 (Kaposi's Sarcoma-Associated Herpes Virus)
HIV	Human Immunodeficiency Virus
HLA	Human Leukocyte Antigen
HRA	Health Research Authority
HSC	Haematopoeitic Stem Cell (Transplant)
HSV	Herpes Simplex Virus
HTA	Human Tissue Act
HTLV	Human T Cell Lymphotropic Virus
IB	Investigators Brochure
ICF	Informed Consent Form
ICH	International Conference On Harmonisation
ICU	Intensive Care Unit
IF	Interstitial Fibrosis
IFN-γ	Interferon Gamma
Ig	Immunoglobulin
IL-2	Interleukin-2
IM	Immune Monitoring
IMP(D)	Investigational Medicinal Product (Dossier)
IOT	Indices Of Tolerance
IP-10	Interferon-Inducible Protein 10
IRB	Independent Review Board
ISF	Investigator Site File
ITT	Intention To Treat
IU	International Units
IV	Intravenous
IVIg	Intravenous Immunoglobulin
LDH	Lactate Dehydrogenase
LFT	Liver Function Tests
LMWH	Low Molecular Weight Heparin

© Copyright: The University of Oxford and Oxford University Hospitals NHS Foundation Trust 2016

MDRD	Modification Of Diet In Renal Disease
MDT	Multi-Disciplinary Team
MHRA	Medicines And Healthcare Products Regulatory Agency
MLR	Mixed Lymphocyte Response
MMF	Mycophenolate Mofetil
MPA	Mycophenolic Acid
MRC	Medical Research Council
mTOR(i)	Mammalian Target Of Rapamycin (Inhibitor)
NDS TRIG	Nuffield Dept. of Surgical Sciences Transplantation Research Immunology Group
NGS	Next Generation Sequencing
NHS	National Health Service
NICE	National Institute for Health and Care Excellence
NIHR	National Institute For Health Research
NIMP	Non-investigational Medicinal Product
NRES	National Research Ethics Service
OCHRe	Oxford Centre for Histopathology Research
OCTRU	Oxford Clinical Trials Research Unit
OTC	Oxford Transplant Centre
OUH	Oxford University Hospitals
PAP	Pulmonary Arterial Pressure
PBMC	Peripheral Blood Mononuclear Cells
PE	Pulmonary Embolism
PI	Principal Investigator
PIS	Participant/ Patient Information sheet
PMG	Project Management Group
PMN	Polymorphonuclear (Leukocyte)
PO	Per Os (By Mouth)
PRA	Panel Reactive Antibody
PT	Prothrombin Time
PTLD	Post-Transplant Lymphoproliferative Disease
qPCR	Quantitative Polymerase Chain Reaction
RA	Renal Association (UK)
R&D	NHS Trust R&D Department
REC	Research Ethics Committee

© Copyright: The University of Oxford and Oxford University Hospitals NHS Foundation Trust 2016

RISET	Reprogramming The Immune System For The Establishment Of Tolerance
RNA	Ribonucleic Acid
RRT	Renal Replacement Therapy
RSI	Reference Safety Information
SAE	Serious Adverse Event
SAP	Statistical Analysis Plan
SAR	Serious Adverse Reaction
sCr	Serum Creatinine
SD	Standard Deviation
SDV	Source Data Verification
SITU	Surgical Interventions Trials Unit (Oxford University)
SMA	Smooth Muscle Antibody
SmPC	Summary of Medicinal Product Characteristics
SOP	Standard Operating Procedure
SUSAR	Suspected Unexpected Serious Adverse Reactions
TA	Tubular Atrophy
TMF	Trial Master File
TMG	Trial Management Group
TR001	Autologous Treg cell product (ATIMP produced by GMP facility)
Tr1	Type 1 Regulatory T Cell
TRALI	Transfusion-Related Acute Lung Injury
Treg	Regulatory T Cell
TRIG	See 'NDS TRIG'
TSC	Trial Steering Committee
TSDR	Treg-Specific Demethylated Region
TSG	Oxford University Hospitals NHS Foundation Trust / University Of Oxford Trials Safety Group
Tx	Transplantation
U&Es	Urea and Electrolytes
UAR	Unexpected Adverse Reaction
ULN	Upper Limit of Normal

© Copyright: The University of Oxford and Oxford University Hospitals NHS Foundation Trust 2016

4. BACKGROUND AND RATIONALE

4.1. Study Rationale and Concept

Patients receiving solid organ transplants must be maintained on multiple immunosuppressive agents in order to prevent the immune system from rejecting the allograft. These medicines are highly efficacious, with one-year renal graft survival in the UK being >95%. However long term outcomes are significantly limited by the serious and life-threatening side-effects of immunosuppressive drugs, which include enhanced rates of infection, malignancy, cardiovascular and metabolic disease. Long term maintenance on immunosuppressive regimens together with treatment of the unwanted sequelae is a huge burden not only for the patient but also for the healthcare system, in terms of resource allocation and expenditure. It is clear therefore that a major goal of transplantation is the minimisation of pharmacological immunosuppression, whilst protecting the allograft from host immune responses.

Regulatory T cells (Treg) are a subset of T cells that act physiologically to prevent autoimmune disease and to limit an 'overshoot' in normal immune responses. A promising approach is to harness this regulatory ability to suppress the transplant rejection response. Having proven the safety and feasibility of Treg-based immunotherapy in the ONE Study (trial identifier: ONETreg1; EudraCT number: 2013-002099-42, co-sponsored by King's College London and Guy's and St Thomas' NHS Foundation Trust, REC number 13/SC/0568), the TWO Study aims to demonstrate the efficacy of this treatment, with the goal of allowing minimisation of pharmacological immunosuppression to a single agent by 6-months post-transplantation.

4.2. Organ Transplantation

Pioneered in the 1950s (Merrill, Murray, Harrison, & Guild, 1956) clinical organ transplantation has revolutionised modern medicine, with remarkable improvements in success rates over the course of the last 60 years, such that it is now the preferred treatment option for many patients with end-stage organ failure (Morris, 2004; Sayegh & Carpenter, 2004).

The major obstacle to transplantation between genetically dissimilar (allogeneic) individuals is rejection of the foreign tissue by the host immune system. The human immune system has evolved into a complex system of cells, antibodies and soluble factors that provides a defence against pathogens. If left unchecked this system recognises allografts as non-self, quickly mounting a destructive response against the organ which would destroy the tissue rapidly without intervention.

The recognition of this process and subsequent development of pharmacological immunosuppressive agents to prevent it has revolutionised the field of transplantation. The past decades have seen a number of newer, more potent and diverse immunosuppressive agents progress to clinical practice with rates of rejection falling as a result. However, for continued graft function patients must continue life-long immunosuppression, exposing them to a cumulative risk of life-threatening complications and co-morbidities (Halloran, 2004).

4.3. Kidney Transplantation and Immunosuppression

Pharmacological immunosuppression used in routine clinical practice has a number of adverse immunological and non-immunological side effects including nephrotoxicity (Naesens, Kuypers, &

© Copyright: The University of Oxford and Oxford University Hospitals NHS Foundation Trust 2016

Sarwal, 2009), metabolic disorders (Mathis, Dave, Knipp, & Friedman, 2004; Montero & Pascual, 2015), cardiovascular disease (Kasiske, Guijarro, Massy, Wiederkehr, & Ma, 1996; Kendrick, 2001), together with life-threatening complications such as infection (Karuthu & Blumberg, 2012) and malignancy (London, Farmery, Will, Davison, & Lodge, 1995; Rama & Grinyo, 2010; Gutierrez-Dalmau & Campistol, 2007). Taken together, the off-target effects with conventional immunosuppression present a formidable barrier to patient longevity and quality of life.

4.4. Immunosuppression Minimisation

Steps have already been taken to reduce the immunosuppressive burden following renal transplantation. The gold standard treatment in the modern era is induction therapy at the time of transplant using basiliximab (an anti-CD25 monoclonal antibody that blocks T-cell signalling and proliferation) followed by maintenance with tacrolimus, mycophenolate and corticosteroids (Ekberg & group, Reduced exposure to calcineurin inhibitors in renal transplantation., 2007). With the advent of newer induction agents, it has been proven that steroids can be successfully avoided or withdrawn with no increase in rejection rates. This was demonstrated in the landmark 3C Study, which used alemtuzumab induction (lymphocyte-depleting anti-CD52 monoclonal antibody) followed by tacrolimus and mycophenolate (Group T. 3., 2014). Compared to the then standard of care, the 3C Study demonstrated a reduction in rejection rates by 58% in the first 6-12 months, with no increase in adverse events (Group T. 3., 2014). This immunosuppressive regimen is being adopted by several UK transplant centres as a new way of minimising long-term immunosuppression load.

To reduce the long-term complications of immunosuppression further there has been much interest in minimisation of immunosuppression to a single agent. Initially the most attractive option would appear to have been sirolimus; a non-nephrotoxic immunosuppressant that is associated with lower rates of de novo malignancy post-transplant (Yanik, Gustafson, & Kasiske, 2015), and which appears to facilitate a regulatory milieu in animal models (Issa, et al., 2010). However, this has proven to be a sub-optimal agent due to exceedingly high rates of adverse side-effects, affecting up to 80% of patients (Welberry Smith, et al., 2008; Lebranchu, et al., 2009), and intolerance leading to drug withdrawal in approximately 25% of patients (Burkhalter, et al., 2012; Weir, et al., 2010; Schena, et al., 2009). Moreover, there have been unacceptably high rates of rejection when used either as monotherapy (17% rejection rates at 12 months (Sutherland, et al., 2014)) or in combination with mycophenolate (14.7% vs 3% in tacrolimus/mycophenolate treated patients at 18 months ([mixed induction therapy]; unpublished data, 3C Study Group)).

An attractive proposition therefore is tacrolimus monotherapy. This is a well-tolerated treatment and a key component of most immunosuppressive regimens in renal transplantation. Several groups have already trialled tacrolimus monotherapy, using alemtuzumab induction. In randomised controlled trials the outcomes appear comparable to tacrolimus and mycophenolate dual therapy with biopsy-proven rejection rates of approximately 10% at one and two years (Chan, et al., 2011; Welberry Smith, et al., 2013). However these are single-centre trials with small numbers of patients and larger trials have reported one-year rejection rates ranging from 14.3% (Villanueva, et al., 2008) to as high as 20% (Marreiter, et al., 2008). The available data is further confounded by the definition of rejection used (clinical decision or biopsy proven, whether borderline histological changes are included, and whether it is that first rejection episodes are reported), and the comparator groups, that are not utilised or have no lymphocyte depletion at induction.

© Copyright: The University of Oxford and Oxford University Hospitals NHS Foundation Trust 2016

4.5. Immunological Tolerance of Transplants

Whilst most patients require lifelong immunosuppression after transplantation, a small number of patients (estimated to be <100 worldwide) maintain good graft function despite cessation of immunosuppression. The term '*operationally tolerant*' has been coined to describe this clinical state (Roussey-Kesler, et al., 2006). Operational tolerance has been documented frequently in rodent transplant models and a useful definition of the state is 'long-term graft function in the absence of long-term immunosuppression' and is immunologically defined as donor-specific unresponsiveness. However spontaneous occurrence in humans is rare (Cippa & Fehr, 2011).

Using rodent models, a number of tolerance-inducing strategies have been successfully developed (Kingsley, Nadig, & Wood, 2007), but translating these into clinically safe and reproducible treatments has proved difficult (Orlando, et al., 2010). Whilst operational tolerance allowing complete withdrawal of immunosuppression remains the ultimate goal, a more realistic objective at present is the induction of an immunological state that facilitates minimisation of immunosuppression, without compromising survival of the transplant (Calne, et al., 1998; Calne R. , 2009). Cell therapy may help achieve this.

4.6. Cell-Based Immunosuppression

In animal models, tolerance can be adoptively transferred to naïve transplant recipients by administering immune modulating cells from an animal that is tolerant to the same donor antigens (Qin, et al., 1993). This transfer of tolerance can be achieved with purified preparations of regulatory immune cells (Jovanovic, Lair, Soulillou, & Brouard, 2008). Various cells within the immune system have regulatory properties, the most compelling evidence being for regulatory T cells (Tregs). Adoptively transferred Tregs can mediate an 'infectious tolerance' in the recipient, whereby they promote a self-perpetuating regulatory response through the recipient's own native T cells, which persists even after the disappearance of the original tolerising cells (Qin, et al., 1993).

4.7. Human Regulatory T Cells (Tregs)

The natural model that demonstrates the importance of Treg function is the IPEX syndrome (immunodysregulation polyendocrinopathy enteropathy X-linked). Patients with this rare inherited disease possess a defective version of the Treg transcription factor Foxp3. These patients suffer profound, severe auto-immune disease (Bacchetta, et al., 2006; Bennett, et al., 2001) which can be reproduced in mouse models which are genetically deficient in Foxp3 (Hori, Nomura, & Sakaguchi, 2003; Fontenot, Gavin, & Rudensky, 2003). Importantly, from this animal work it has been shown that genetic delivery of Foxp3 can convert non-regulatory CD4+ T cells into cells capable of preventing auto-immunity. These studies highlight the role of active regulation mediated by Tregs as an essential part of the normal immune system. It is therefore a logical step to try and harness this cellular mechanism therapeutically in order to control damaging immune responses in the clinical setting.

4.8. Pre-clinical Studies of Treg Cellular Therapy

Various animal models have demonstrated the efficacy of Tregs in promoting a tolerogenic state in a number of different organ transplants. Using a mouse model, ex vivo-expanded Tregs were shown to migrate to and proliferate in allografts, their draining lymph nodes, and induce transplant tolerance (Golshayan, et al., 2007). Our group having been the first to demonstrate human Tregs' ability to prevent

© Copyright: The University of Oxford and Oxford University Hospitals NHS Foundation Trust 2016

rejection of human vessel allografts (Nadig, et al., 2010), went on to prove that Treg therapy can be successfully combined with sirolimus immunosuppression, which may enhance Treg function (Hester, Schiopu, Nadig, & Wood, 2012). Further details of the pre-clinical studies can be found in the Investigator Brochure (IB).

4.9. Previous Clinical Studies of Treg Cellular Therapy

Despite the presence of immunosuppression, Treg concentrations in transplant recipients are similar to that of healthy controls, and these cells demonstrate the ability to suppress T cell alloresponses in vitro (Game, Hernandez-Fuentes, Chaudhry, & Lechler, 2003). Indeed, manipulating immunosuppression induction and subsequent therapy may induce a pro-tolerogenic state. Studies have demonstrated that after initial lymphocyte depletion with alemtuzumab, reconstitution to near normal cell counts takes approximately 6 months (Trzonkowski, Zilvetti, Friend, & Wood, 2006; Tronkowski, et al., 2008). However, compared with other induction regimens, patients treated with alemtuzumab reconstitute with an enhanced proportion of Tregs (Bloom, et al., 2008).

Several clinical trials outside of solid organ transplantation have demonstrated safe and favourable clinical outcomes with Treg therapy. In Minnesota, a clinical trial evaluated the safety profile of partially HLA-matched ex vivo-expanded Tregs for the treatment of graft-versus-host disease (GvHD) post-haematopoeitic stem cell (HSC) transplantation (Brunstein, et al., 2011). The study was designed as a Phase I dose-escalation trial and reported a reduced incidence of grade II-IV acute GvHD in the test group of 23 patients compared to 108 identically-treated historical controls not receiving Treg therapy. An Italian study was also performed to assess the safety and efficacy of human Tregs in prevention of GvHD in 28 patients with high-risk acute leukaemia undergoing HLA-haploidentical HSC and conventional T cell transplants. GvHD did not develop in 25 out of 29 patients. Furthermore, patients displayed overall faster post-transplant immune reconstitution as well as a reduction in the risk of CMV reactivation (Di Ianni, et al., 2011). Expanded autologous Tregs up to a dose of $30x10^6$ cells/kg have also been administered to 12 paediatric patients (age range 8-16 years) with new onset diabetes for the protection of pancreatic islets. No adverse events were associated with cell administration and treated patients have demonstrated lower exogenous insulin requirements than untreated controls (Marek-Trzonkowska, et al., 2014).

4.10. Clinical Treg Therapy in Renal Transplant Recipients

Four groups have now published initial data using Treg therapy in renal transplant recipients. In Chicago nine living donor kidney recipients have received autologous, polyclonally-expanded Tregs in a dose-ranging study up to a maximum dose of $5x10^9$ cells. Alemtuzumab induction was followed by mycophenolate and tacrolimus; the tacrolimus being converted to sirolimus at day +30. At day +60, Tregs were infused. All infusions were given without incident and achieved a 9- to 20-fold increase in peripherally circulating Treg concentrations (identified as CD4+CD127-CD25^{High}FOXP3 cells) without any infectious complications or rejection noted (protocol biopsies in all nine recipients) (Skaro, et al., 2016). In Boston, two living donor recipients have been infused with $2x10^4$ cells/kg donor-reactive Tregs at 10 days post-transplant. Corticosteroids were given at the time of transplantation, followed by mycophenolate, tacrolimus and steroids, the latter of which was stopped at 15 weeks. No reaction to cell infusions was noted, and graft function remains excellent at eight months (Markmann, et al., 2016).

© Copyright: The University of Oxford and Oxford University Hospitals NHS Foundation Trust 2016

In addition, in San Francisco Tregs have been used as a clinical intervention in two transplant recipients with sub-clinical biopsy-proven transplant inflammation that does not meet the criteria for rejection (Banff criteria). Both patients were maintained on prednisolone, MMF and tacrolimus, and each received a single infusion of $320x10^6$ Tregs. Infusions were well tolerated and at 6-months post-infusion, protocol biopsies demonstrated improvement in the inflammatory infiltrates, with stable graft function and no infectious or rejection episodes in the interim. A potentially promising aspect of this study is that Tregs were isolated and expanded (>100-fold increase in both cases) after transplant and whilst the patients were on maintenance immunosuppression (Chandran, et al., 2016). This broadens the clinical utility of Treg therapy, to potentially include deceased-donor transplant recipients whose surgery date is unpredictable making pre-operative blood donation for Treg production logistically challenging.

In the largest series to date, the ONE Study research collaborative from Oxford and London infused 12 living donor kidney transplant recipients with autologous polyclonally-expanded Tregs in a dose escalation safety trial administering doses of $1x10^6$; $3x10^6$; $6x10^6$and $10x10^6$ cells/kg at day five post-transplant. Again, no infusion reactions were noted and in a median follow-up of 10 months (1-15 months) no biopsy-proven rejection episodes were observed. All patients have stable allograft function (median creatinine 128 (90–176) µmol/L) on MMF and tacrolimus dual therapy (Bushell, et al., 2016). This dose escalation study has shown the clinical safety of a dose of $10x10^6$ autologous polyclonally-expanded Tregs resulting in the choice of this dose for use in the TWO Study. No induction therapy was given in the ONE Study patients receiving Treg cell therapy at transplantation. The TWO study has a different approach with the use of a single dose of the powerful lymphocyte depleting antibody Alemtuzumab at the time of transplantation. It would be futile to give Treg cell therapy in the early phase post transplantation as these cells would be rapidly depleted by residual Alemtuzumab. Consequently we plan to wait until the T lymphocytes have largely re-populated 6 months post-transplantation within a native Treg cell rich environment for the maximal impact of infusion of autologous polyclonal Tregs. Other studies have utilised up to $20x10^6$ Tregs with no adverse effects (Marek-Trzonkowska, et al., 2014).

5. OBJECTIVES AND OUTCOME MEASURES

5.1. Primary Objective

To compare the efficacy of autologous regulatory T cell therapy used in conjunction with tacrolimus to standard of care immunosuppression (MMF and tacrolimus) in preventing acute biopsy-proven rejection in renal transplant recipients.

5.2. Primary Outcome Measure

Incidence of biopsy-confirmed acute rejection (BCAR) in the 6 to 18-month period post-transplantation, between groups. Histopathological grading of biopsy material will be performed according to the Banff criteria (Haas, et al., 2014). The management of patients according to histopathological findings will be according to the standard local clinical protocol. As the intervention does not take place until 6 months post-transplant, early acute rejection episodes (<6 months) are expected to be similar in the two treatment arms and are not included in the test for efficacy, although they will be reported.

© Copyright: The University of Oxford and Oxford University Hospitals NHS Foundation Trust 2016

BCAR has been selected for the following reasons:

The incidence of BCAR has become an accepted short-term surrogate marker for kidney allograft survival and is widely used as a primary endpoint in kidney transplantation trials of immunosuppressive therapies (LAchenbruch, Rosenberg, Bonvini, Cavaille-Coll, & Colvin, 2004; Fleiner, Fritsche, Glander, Neumayer, & Budde, 2006). The historical evidence base has informed the expectations underlying the statistical design of this study.

Allograft biopsy is the ‘gold standard’ for the evaluation of kidney allograft dysfunction (Al-Awwa, Hariharan, & First, 1998; Pascual, et al., 1999) and the histopathological criteria for the diagnosis of acute rejection are standardised and unambiguous (Haas, et al., 2014). Histological assessment will be based on the Banff Classification for Kidney Allograft Pathology and this system is used by renal histopathologists throughout the UK. In addition, Professor Ian Roberts of the John Radcliffe Hospital, Oxford, was appointed as the independent Central Pathologist for the entire consortium of the ONE Study and is therefore a leading authority on renal allograft histopathology in the context of cellular therapy-treated patients. Professor Roberts will report the histopathology in this trial.

BCAR represents the most pragmatic and testable primary endpoint for treatment efficacy within the time and resource constraints of a transplantation trial.

5.3. Time-Point of Evaluation of Primary Outcome Measure

Data for demonstration of the primary outcome will be collected in months 6-18 post-transplantation.

Histopathological samples will be taken at 5 months (protocol biopsy) in kidney transplant recipients randomized to the Treg therapy arm. This biopsy will confirm suitability for the Mycophenolate Mofetil immunosuppression taper and cessation prior to Treg therapy at Month 6. The 9 month protocol biopsy will be performed in all participants including the control arm to allow a histological comparison of the impact of Treg therapy in the treatment arm.

Any other biopsies performed during follow-up will be at the discretion of the responsible clinician and will be deemed ‘for-cause’. These will be reviewed and reported by the study pathologist using the Banff criteria and results will be available to the trial investigators and documented in the CRF.

5.4. Secondary Objectives

Secondary endpoints will be used to assess the safety, feasibility and potential additive benefits of both cellular therapy and the associated immunosuppression minimisation. Secondary objectives will therefore be assessed as:

A. Indicators of influence of Treg administration on graft outcome

B. Markers of over-suppression of the immune system

C. Signs of chronic toxicity associated with infusion of cell products

D. Patient quality of life

© Copyright: The University of Oxford and Oxford University Hospitals NHS Foundation Trust 2016

5.5. Secondary Outcome Measures

Indicators of influence of Treg administration on graft outcomes will include:

- Impact on acute rejection
  - Time to first acute rejection episode
  - Severity of acute rejection episode based on response to treatment and histological scoring
  - Total immunosuppressive burden at the final trial visit
  - Incidence of graft loss through rejection

- Success in reduction of immunosuppression
  - Proportion of patients on tacrolimus monotherapy at the end of the study

- Prevention of chronic graft dysfunction
  - Renal function
  - Chronic allograft dysfunction or interstitial fibrosis/tubular atrophy (IF/TA) assessed by clinical (impairment of eGFR) and histopathological (Banff staging) measures

- Avoidance of drug-related complications by immunosuppressant reduction
  - Incidence of drug-related adverse events

- Patient survival

Markers of over-suppression of the immune system will include:

- Incidence of serious and/or opportunistic infections (especially CMV, EBV and polyoma (BK) virus)
- Incidence of neoplasia

Signs of chronic toxicity associated with infusion of cell products will include:

- Incidence of auto-immune disorders
- Incidence of anaemia, cytopaenias, or biochemical disturbances unrelated to the function of the transplanted kidney

Impact of Treg treatment on patient quality of life will be measured in both arms of the study at visits 0, 8, 18 and 19 using:

- SF-36 questionnaire
- EQ-5D-5L questionnaire

5.6. Time-point of Evaluation of Secondary Outcome Measures

All secondary outcomes will be assessed throughout the 60 months post-transplant, with data collected at study visits and any episodes of adverse, or serious adverse events as they arise. A schematic diagram of data collection by times points/study visit can be seen in *Table 1*.

© Copyright: The University of Oxford and Oxford University Hospitals NHS Foundation Trust 2016

5.7. Exploratory Objectives and Outcome Measures

A number of immune monitoring techniques will be used to:

- Predict tolerance and rejection

- Detect an effect of Treg therapy, including an assessment of Treg survival and function

In particular, an assessment will be made of Treg survival and function in vivo as well as alterations in immune phenotype and function using analyses as below:

- Flow cytometric cell phenotype analysis using standardised immune monitoring panels
- T cell functional assays against donor and third-party antigen including CD154/CD137 ratio analysis
- Serum cytokine analysis and metabolic profiling
- Gene expression and RNA sequencing analyses including a Treg-Specific Demethylated Region (TSDR) analysis and T-Cell Receptor Sequencing
- Analysis of transplant biopsy immune infiltrate for cell phenotype and transcripts

Finally, analysis of viral infections within transplant recipients will be performed using next generation sequencing.

5.8. Time-point of Evaluation of Exploratory Outcome Measures

All exploratory outcomes will be assessed at defined time points pre-transplantation and during an 18 month period post-transplantation in the kidney transplant recipient and pre-transplantation on one occasion in the living kidney donor. Exploratory data will also be collected during any episodes of rejection, should they occur. Samples for viral monitoring will be collected according to local clinical protocols. A schematic diagram of data collection by times points/study visit can be seen in *Table 1*.

6. TRIAL DESIGN

The study is designed as a single-centre, open-label randomised-controlled Phase IIb trial.

6.1. Trial Duration

The trial will run for 98 months with a recruitment phase of 36 months and the last visit of the last patient at 98 months. Individual kidney transplant recipient involvement in both the control and treatment arm of the trial are scheduled for 8 weeks from consent to kidney transplantation and a further 260 weeks post-transplantation. Almost all (>90%) treatment failures defined by the primary end-point are expected to occur within 78 weeks post-transplantation (*Figure 1*). Historically 85.7% of first acute rejection episodes occurring within 3-years post transplantation arise within one year; 9.7% of cases present in the second year and 4.5% emerge during the third year (Opelz, Dohler, & Report, 2008).

The trial will run for a maximum of 12 weeks for the living kidney donors from the point of consent to the time of donation. Clinical follow up post-donation is as per standard NHS clinical care outside of the trial. There is no follow up for these donors in relation to the trial.

© Copyright: The University of Oxford and Oxford University Hospitals NHS Foundation Trust 2016

Figure 1. Cumulative incidence of first rejection episodes in first three-years post-transplant

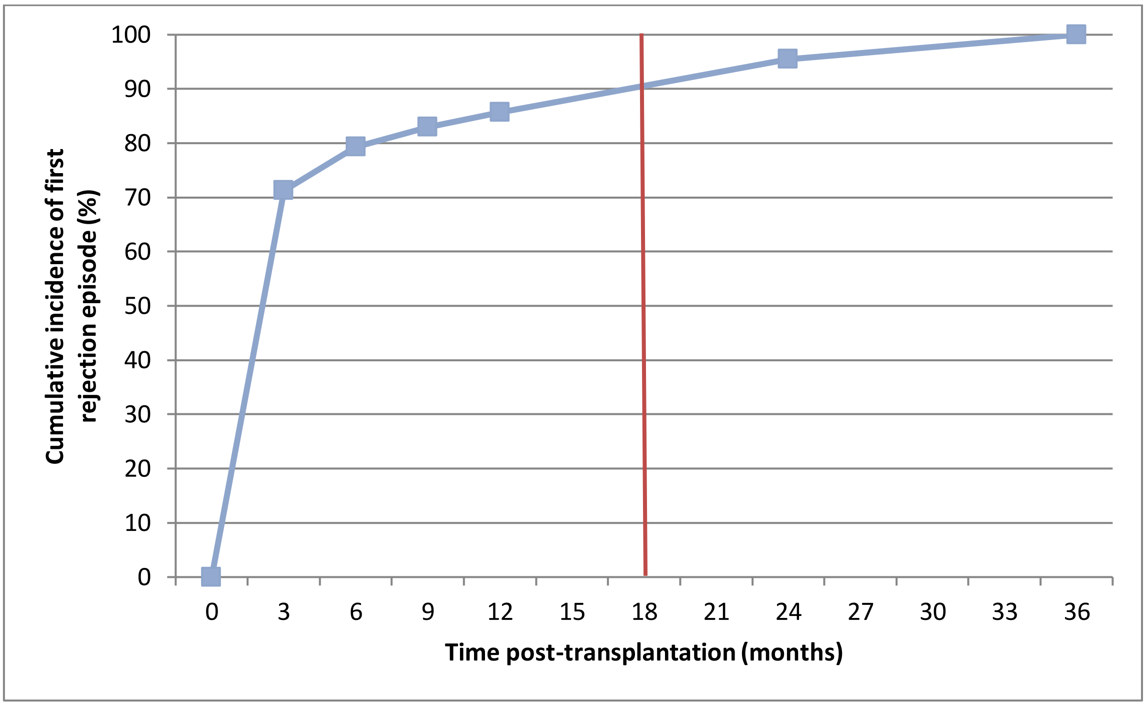

Data adapted from (Opelz, Dohler, & Report, 2008).

6.2. Definition of Trial Time Measurements

All study time points are scheduled relative to the day of transplantation surgery, 'Day 0', which is defined as the day of kidney reperfusion. This occurs approximately 8 weeks post randomisation. All the time points in the TWO Study are measured in days and weeks apart from biopsies which are measured in months. Consequently time points range from the Pre-Visit at a maximum of 12 weeks pre-transplantation at time of consent to Visit 23 at 260 weeks post transplantation in both the control and treatment arm of kidney transplant recipients.

The live kidney donors will be consented at a Pre-Visit at a maximum of 12 weeks before donation and remain in the trial until the day of kidney donation and transplantation at Day 0.

6.3. Trial Follow-Up

The majority of trial-related procedures will occur during standard clinical follow-up visits for kidney transplant recipients in both the control and treatment arms and only rarely at additional time points for collection of blood, in order to minimise inconvenience to participants. In comparison to the control arm, the Treg infusion treatment arm patients require six additional visits: for procurement of blood for TR001 manufacture (V1), at the time of the first protocol biopsy and MMF reduction at 5 months (V7), cessation of Mycophenolate Mofetil and infusion of Treg therapy (V9), two safety visits (V10 and V11), and one further visit (V15) following the protocol biopsy at 9 months post-transplantation to supervise reduction of tacrolimus therapy.

© Copyright: The University of Oxford and Oxford University Hospitals NHS Foundation Trust 2016

Live kidney donors will attend for a pre-visit to obtain consent and provide baseline samples for exploratory monitoring. Clinical data will be documented in the CRF related to the donor until the point of living kidney nephrectomy on Day 0.

7. Trial Participants

7.1. Trial Participants

68 adults with end-stage kidney disease assessed as suitable for renal transplantation by their direct care team will be recruited along with their proposed kidney donor where possible. This gives a combined maximum total of 136 participants Transplant recipients will be randomised on a 1:1 basis to receive cell therapy or control treatment.

The kidney recipient's donor will also be recruited to generate baseline data and will be asked to provide one set of blood samples only to inform the recipient IM analyses. However, if the donor is a paired exchange or altruistic donor then they will not be recruited to the trial as these donors remain anonymous to the transplant recipient. Paired donor exchanges take place where two donors exchange their recipients in returned for ABO or enhanced HLA matching. The recipient of a paired exchange donor transplant will be recruited as normal.

7.2. Kidney Donor Inclusion Criteria

A prospective donor is eligible if all of the following inclusion criteria apply:

- Eligible for live kidney donation
- Aged at least 18 years
- ABO blood group compatible with the organ recipient
- Willing to provide personal, medical and biological data for the trial analysis
- Willing and able to provide a blood sample for the immune monitoring assays
- Willing and able to give informed consent for participation in the trial[*]

7.3. Kidney Recipient Inclusion Criteria

A prospective kidney transplant recipient is eligible for enrolment into the study if all of the following inclusion criteria apply:

- Chronic renal insufficiency necessitating kidney transplantation and approved to receive a kidney allograft from a living donor
- Willing and able to give informed consent for participation in the trial[*]

[*] *For patients unable to read and/or write, the willingness of the consentee to give informed consent will be confirmed by the signature of an independent witness when the consentee has fully understood oral information given by the investigator.*

© Copyright: The University of Oxford and Oxford University Hospitals NHS Foundation Trust 2016

- Aged 18 years or above
- In the Investigator's opinion, is able and willing to comply with all trial requirements
- Able to commence the immunosuppressive regimen at the protocol-specified time point
- Female participants of child bearing potential and male participants whose partner is of child bearing potential must be willing to ensure that they or their partner use highly effective contraception during the first 18 months post-transplant (see section on Contraception)
- Willing to allow his or her General Practitioner and consultant, if appropriate, to be notified of participation in the trial.

7.4. Kidney Donor Exclusion Criteria

If a prospective donor fulfils any of the following criteria, they are ineligible for the trial:

- Exposure to any investigational agents at the time of kidney donation, or within 28 days prior to kidney donation
- Any form of substance abuse, psychiatric disorder, or other condition that, in the opinion of the Investigator, may invalidate communication with the Investigator designated personnel
- Is a paired exchange donor
- Is an altruistic donor

7.5. Kidney Recipient Exclusion Criteria

The participant may not enter the trial if ANY of the following apply:

- Patient has previously received any tissue or organ transplant
- Known contraindication to the protocol-specified treatments or medications
- ABO blood group incompatible with donor
- Calculated reaction frequency (CRF) of >40% within 6 months prior to transplant
- Previous treatment with any desensitisation procedure (with or without IVIg)
- Concomitant malignancy or history of malignancy within 5 years prior to planned study entry (excluding successfully treated non-metastatic basal or squamous cell carcinomas of the skin)
- Serologically positive for anti-HIV-1/2 Ab, HbsAg, anti-HBcAb, antiHCV Ab, anti-HTLV-1/2 Ab or syphilis (treponema palladium)
- Significant liver disease, defined as persistently elevated ALT levels >3 x upper limit of normal range (ULN)
- Any other significant disease or disorder which, in the opinion of the Investigator, may either put the participants at risk because of participation in the trial, or may influence the result of the trial, or the participant's ability to participate in the trial

© Copyright: The University of Oxford and Oxford University Hospitals NHS Foundation Trust 2016

- Participation in another clinical trial during the study or within 28 days prior to planned study entry
- Female participant who is pregnant, lactating or planning pregnancy during the course of the trial
- Psychological, familial, sociological, or geographical factors potentially hampering compliance with the study protocol and follow-up visit schedule
- Any form of substance abuse, psychiatric disorder, or other condition that, in the opinion of the investigator, may invalidate communication with the investigator and/or designated personnel

7.6. Contraception

Female participants of child bearing potential and male participants whose partner is of child bearing potential must be willing to ensure that they or their partner use highly effective contraception during the initial 18 month investigatory period of the trial post transplant. Female of child bearing potential are female patients who have experienced menarche and who are not post-menopausal or permanently sterilised (e.g. by tubal occlusion, hysterectomy, bilateral salpingectomy). Highly effective methods of birth control are those with a failure rate of <1% per year when employed consistently and correctly. These include:

- Combined hormonal contraception associated with inhibition of ovulation (oral, intravaginal or transdermal)

- Progestogen-only hormonal contraception associated with inhibition of ovulation (oral, injectable, or implantable)

- Intrauterine device

- Intrauterine hormone-releasing system

- Bilateral tubal occlusion

- Vasectomised partner

- True abstinence, when this is in line with the preferred and usual lifestyle of the subject. [Periodic abstinence (e.g., calendar, ovulation, symptothermal, post-ovulation methods), declaration of abstinence for the duration of exposure to IMP, and withdrawal are not acceptable methods of contraception].

All transplant recipients in this trial must agree to employ highly effective methods of birth control throughout the trial duration. While this is required for patients receiving the ATIMP, it is NHS standard of care for highly effective contraception to be used for at least 12 months after transplantation for patients on MMF immunosuppression (NIMP in this trial) and therefore required for transplant recipients in both trial arms for 18 months post-transplant.

© Copyright: The University of Oxford and Oxford University Hospitals NHS Foundation Trust 2016

8. TRIAL PROCEDURES

8.1. Initial Contact and Screening

There is a well-defined clinical pathway for patients being assessed and worked-up for living donor transplantation which involves a multidisciplinary team. The direct care team will meet weekly with the living donor nursing team to review all patients currently undergoing living donor work-up and identify donor and recipient pairs potentially suitable for the TWO Study Clinical Trial. The direct care team will contact eligible patients and provide them with the donor and recipient patient information sheets (PIS). On occasion a trial investigator may be a member of the clinical care team for a specific donor or recipient.

Individuals potentially interested in participation after provision of the PIS will contact the Trial Investigators directly, when they may request further information and discuss if they are willing to participate with the Trial Investigators. This will be an informal discussion in the first instance. If there is no response after two weeks, the direct care team may make a second approach. If eligible and willing to participate the donor and recipient will attend a pre-visit where written informed consent will be taken by a Trial Investigator after checking the donor and recipient fulfil all inclusion and exclusion criteria from existing medical records. A trial assignment number will be allocated and the recipient will be randomised to either the control or treatment arm of the trial.

See section 8.2 below for further details on the consent process.

The investigator is responsible for ensuring that study-related activities do not commence prior to a participant providing written informed consent.

8.2. Informed Consent

The participant must personally sign and date the latest approved version of the Informed Consent form at the Pre-Visit (V0), before any trial specific procedures are performed. Written and verbal versions of the Participant Information and Informed Consent will be presented to the participants detailing no less than: the exact nature of the trial; what it will involve for the participant; the implications and constraints of the protocol; the known side effects and any risks involved in taking part. It will be clearly stated that the participant is free to withdraw from the trial at any time for any reason without prejudice to future care, without affecting their legal rights and with no obligation to give the reason for withdrawal.

The participant will be allowed as much time as they wish to consider the information, and the opportunity to question the Investigator, their GP or other independent parties to decide whether they will participate in the trial. Written Informed Consent will then be obtained by means of participant dated signature and dated signature of the person who presented and obtained the Informed Consent. The person who obtained the consent must be suitably qualified and experienced, and have been authorised to do so by the Chief/Principal Investigator. Specifically, this will be a GCP-trained Consultant Nephrologist or Clinical Fellow who specialises in renal transplantation. A copy of the signed Informed Consent will be given to the participant, a copy kept in the site file, and a copy kept in the patient's medical records (electronically if EPR is used). The original signed form will be retained at the trial site.

© Copyright: The University of Oxford and Oxford University Hospitals NHS Foundation Trust 2016

8.3. Randomisation

Randomisation will occur after written informed consent is obtained and eligibility is confirmed at the Pre-Visit (V0). Participants will be randomised on a 1:1 basis to either one Treg cell infusion at 6 months (week 26) post-transplantation or standard care. Randomisation will be performed using a minimisation algorithm including a probabilisticelement to ensure balanced allocation of participants across the treatment groups stratified by:

- Ethnicity (white vs. other)
- HLA-DR mismatch (0 vs 1 or 2)

The first 6 participants (approximately 10% of the sample size) will be randomised using simple randomisation to seed the minimisation algorithm. This will be designed in conjunction with the study statistician and performed off-site using a validated computer-based system (RRAMP, Registration/Randomisation and Management of Product) at the Oxford Clinical Trials Research Unit (OCTRU). As the TWO study is an open-label trial no blinding or code-breaking protocols are required.

9. Donor Study Visits

Donors will only attend the pre-visit, Visit 2 and the donation visit (i.e. day 0 – operation). All visits coincide with standard clinical visits.

9.1. Visit 0 (Pre-visit)

The pre-visit will coincide with a standard clinical visit. Recipients and their donors identified from a previous MDT as potentially suitable for recruitment will have an eligibility checklist completed at this visit. If passed, consent will be taken and unique trial identifier issued.

9.2. Visit 2 (both arms)

Visit 2 will take place between 3 and 1 weeks prior to the transplant operation.

Donor patients will attend for collection of baseline data as well as a physical examination to include a 12-lead ECG, vital signs, and measurement of BMI. The following donor data will be collected from the NHS Clinical Data package utilised for clinical work-up of donor suitability and stored in the electronic database (eCRF):

- Date of Birth
- Gender
- Ethnicity
- Relationship of the donor to the recipient
- ABO and Rhesus blood types
- HLA-type
- Serology (CMV, EBV, HBV, HIV, VZV, HTLV, Syphilis)
- Infectious disease status
- Relevant medical history
- Results of split kidney function tests if performed

© Copyright: The University of Oxford and Oxford University Hospitals NHS Foundation Trust 2016

Finally, the donor will give a sample of 40mls blood to be used in immune monitoring assays to permit analysis of the recipient's immune response specifically to donor antigens. Such analysis forms an important part of the experimental outcomes.

9.3. Day 0 - Operation (Both arms)

The donor will be admitted via the admission unit before the donor kidney retrieval operation. Patients will undergo a full clerking, including a physical examination, with a particular emphasis on identifying any recent or ongoing infections which might preclude donation. A trial Investigator will ensure that all donor eligibility criteria have been met.

The donor operation will proceed first and the removed kidney will be placed in cold storage prior to transplantation into the recipient which will occur sequentially on the same day. Both surgical procedures will be performed as part of standard NHS clinical care.

10. Recipient Study Visits

Apart from Visits 1, 7, 9, 10, 11, and 15, all visits will coincide with a standard clinical follow up appointment. This section details visits in which members of the research team may interact with participants during their visit. There may be unscheduled clinical visits which are clinical only and in which there is no interaction with the research team, although information pertaining to medication levels and clinical assessments may be shared with the research team and entered into the eCRF.

Patients randomised to the control arm will not attend for the cell isolation visit (Visit 1), undertake a protocol biopsy at week 22 (Visit 7), stop MMF or receive Treg infusion at Visit 9, or have the subsequent visits at weeks 27 and 28 (Visits 10 & 11). Both control and treatment arms will have a protocol kidney transplant biopsy at Visit 13 and attend Visit 14 for the biopsy result. Only the treatment arm will have tacrolimus immunosuppression reduced following the protocol biopsy at Visit 14 if there is no evidence of histological changes of rejection. An additional visit, V15, applies only to the treatment arm only to monitor the safety of tacrolimus reduction.

The majority of visits will take place in the out-patient clinic of the OTC. Exceptions are the cell procurement visit (V1), Visits 7 and 13 (Protocol biopsies), and the cell infusion visit (V9), which will all take place in the Renal Day-Case Unit, based at the Churchill Hospital in Oxford. This will allow close monitoring of patients, with equipment available to manage all possible complications.

10.1. Pre-visit (Visit 0)

The pre-visit will coincide with a standard clinical visit. Patients identified from a previous MDT as potentially suitable for recruitment will have an eligibility checklist completed at this visit. If passed, consent will be taken. Baseline demographic and medical/biological data including past medical history and current medications will be collected for trial participants after signing the written informed consent. This will permit characterisation of each subject in terms of immunological risk and ensure compliance with aspects of the trial eligibility criteria. Recipients in both arms will be asked to complete a baseline EQ-5D-5L and SF-36 quality of life questionnaire to be completed by the participant whilst they are alone. Patients will proceed to randomisation. All patients will receive a unique trial identifier.

© Copyright: The University of Oxford and Oxford University Hospitals NHS Foundation Trust 2016

10.2. Visit 1 – Treg arm only

Patients in the Treg therapy arm will be admitted to the Churchill Hospital Day-Case Unit 3 to 6 weeks before the planned operation date for procurement of 370ml of blood by peripheral venepuncture. Observations and an ECG will be taken. Patients will be asked if there has been any change in their clinical condition and this will be documented if necessary. Blood will be collected under sterile conditions, sealed, packaged and transported to the GSTT GMP Unit under a third-party HTA Licence. The pulse rate, blood pressure and respiratory rate will be monitored throughout the venesection and for 30 minutes after completion of venesection. In order to comply with the EU Tissues and Cells Directive, a sample for serology testing will also be collected at the time of cell isolation or, if not possible, within 7 days of cell isolation.

If insufficient cells are produced from the donor blood sample or the product does not fulfil the QC specifications, the potential recipient will be given the option of providing a second 370ml venesection sample a minimum of 2 weeks later if haemoglobin levels are >100g/L. This may delay the proposed donation and transplant by a maximum of 12 weeks and will only be considered with the consent of the donor and recipient and if clinically appropriate.

10.3. Visit 2 – Both arms

This visit coincides with a standard NHS follow up visit. This will take place between 3 to 1 weeks prior to the planned operation date. Patients will attend for collection of baseline data as well as tests as per standard NHS care: bloods (U&E, FBC, LFTs, bone profile, lipid profile, CRP, glucose, DSA), a clinical urine test (spot urinary protein:creatinine ratio), a pregnancy test, and the first trial-specific immune monitoring blood tests. In addition patients will undergo a physical examination to include a 12-lead ECG, vital signs, and measurement of BMI.

The following recipient data will be collected from the NHS Clinical Data package obtained during the assessment process for suitability for transplantation as part of standard clinical care and included in the electronic database (eCRF):

- Date of birth
- Gender
- Ethnicity
- HLA-type and number of mismatches with the prospective organ donor
- ABO and Rhesus D blood types
- cRF grade (and date of test)
- Tests for pre-formed antibodies
- Viral serology (CMV, EBV, HBV, HCV, HIV, VZV, HTLV, Syphilis)
- Infectious disease status
- Medical history, including:
    - Cause of end-stage kidney disease
    - Type and duration of pre-transplant treatment modalities for the primary diagnosis (e.g. dialysis modalities)
    - Co-morbidities and risk factors with known influence on transplantation outcome (e.g. cardiovascular disease, diabetes, infections, hyperlipidaemia)
    - Evidence of possible immunological sensitisation (pregnancies, blood transfusions)

© Copyright: The University of Oxford and Oxford University Hospitals NHS Foundation Trust 2016

- Other relevant medical history within 4 weeks prior to enrolment[1]

CMV, VZV, and EBV serology will not be repeated if the patient is already known to be seropositive in the past as indicated by his/her medical records. Also current data will be used for HLA, ABO and Rhesus type.

Further physical, biochemical and anthropometric measures taken at this and subsequent visits are detailed in *Table 2*.

10.4. Day 0 (Operation, standard care – both arms)

The recipient will be admitted to hospital prior to transplantation. Patients will undergo a full clerking, including a physical examination, with a particular emphasis on identifying any recent or ongoing infections which might preclude transplantation, vital signs, and BMI measurement. A trial investigator will ensure that all recipient eligibility criteria have been met. The donor operation will proceed first and the removed kidney will be placed in cold storage. Transplant surgery into the recipient will occur sequentially on the same day. Both surgical procedures will be performed as part of standard NHS clinical care. Immunosuppression will be commenced as per NHS standard care (MMF, methylprednisolone, tacrolimus and alemtuzumab). Concomitant medication will be documented. Clinical blood and urine tests will be taken as per NHS standard care (U&Es, FBC, LFTs, bone profile, glucose, coagulation screen, G&S, pregnancy test).

Data that can only be provided after the surgical procedure will be collected on a dedicated transplantation page in the electronic database. These data will include:

- Time and date of transplantation[2]
- Duration of organ explantation and warm ischaemia[3]
- Duration of organ implantation and cold ischaemia[4]
- Type of surgical technique (e.g. laparoscopic, flank incision, robotic)
- Anatomical abnormalities of the donor organ (if applicable)
- Surgical complications (if applicable)
- Early post-operative renal function (e.g. acute kidney injury, delayed graft function)

10.5. Visit 3 (both arms)

This visit coincides with a standard NHS follow up visit. This visit takes place approximately 1 week post-operatively. Standard clinical assessments are made as well as urine and blood tests as per standard NHS

[1] Reasonable efforts will be made to determine prior therapies. For each medication, the name of the agent, and the duration of treatment, should be recorded in the electronic database.

[2] The time of transplantation is defined as the time of graft reperfusion
[3] Warm ischaemic time is defined as the time from arterial clamping of the kidney to storage of the graft on ice
[4] Cold ischaemic time is defined as the time from storage on ice to reperfusion with recipient blood (recipient arterial unclamping)

© Copyright: The University of Oxford and Oxford University Hospitals NHS Foundation Trust 2016

care (U&Es, FBC, LFTs, bone profile, glucose, tacrolimus level, urine protein:creatinine ratio). A physical examination will be performed to include vital signs and BMI measurement and concomitant medication documented. Details of these assessments and results will be made available to the trial team.

10.6. Visit 4 (both arms)

This visit coincides with a standard NHS follow up visit. At this visit trial-specific immune monitoring blood samples will be taken as per *Tables 1, 2 and 3*. A physical examination will be performed to include vital signs and BMI measurement. Blood tests will be performed as per standard NHS care (U&Es, FBC, LFTs, bone profile, CRP, glucose) and urinary protein:creatinine ratio. Concomitant medication will be documented as per standard NHS care and any changes recorded in the eCRF.

10.7. Visit 5 (both arms)

This visit coincides with a standard NHS follow up visit and takes place at approximately week 12 post-operatively. Visit 5 is a standard clinical visit for clinical assessment as per standard NHS care that coincides with an immune monitoring visit where trial-specific immune monitoring blood samples will be taken as per *Tables 1, 2 and 3*. NHS standard care tests will include blood U&Es, FBC, LFTs, bone profile, glucose, tacrolimus level, DSA and urinary protein:creatinine ratio. A physical examination will be performed to include vital signs and BMI measurement.

Furthermore, at this visit, changes will be made to MMF immunosuppression dosing in the Treg arm only as follows: If trough tacrolimus levels remain ≥5 ng/mL, the MMF dose will be reduced to 500mg BD. If the trough tacrolimus level is <5 ng/mL, the MMF will continue at 750 mg BD, and the tacrolimus dose will be adjusted by the patient's nephrologist, with a repeat trough level 3 – 5 days later (as per standard clinical practice). This step will be repeated until the tacrolimus level reaches ≥5 ng/mL, and only then will the MMF be reduced to 500mg BD.

Concomitant medication in both arms of the study will be documented as per standard NHS care and any changes recorded in the eCRF.

10.8. Visit 6 (both arms)

This visit coincides with a standard NHS follow up visit and takes place at approximately week 14 post-operatively. Visit 6 is a standard clinical visit for clinical assessment. NHS standard care tests will include blood U&Es, FBC, LFTs, bone profile, glucose, tacrolimus level and urinary protein:creatinine ratio. Details of these assessments and results will be made available to the trial team. A physical examination will be performed to include vital signs and BMI measurement. Concomitant medication will be documented as per standard NHS care and any changes recorded in the eCRF

10.9. Visit 7 (Biopsy - Treg arm only)

The patient will be admitted to the Renal Day-case Unit for a renal transplant biopsy approximately 22 weeks post operatively. Blood will be taken for research for standard panels (FBC, U&E, LFTs, bone profile, glucose, tacrolimus level, coagulation screen, G&S, DSAs), urinary protein:creatinine ratio as well as immune monitoring panels. The patient will be consented for the biopsy procedure according to local clinical policy. The biopsy will only be performed if the patient is on no concomitant anticoagulation, has

© Copyright: The University of Oxford and Oxford University Hospitals NHS Foundation Trust 2016

no bleeding diatheses identified on blood tests, blood pressure on the day is <160/90 mmHg, and, bedside ultrasound demonstrates no hydronephrosis or obstruction. A physical examination will be performed to include vital signs and BMI measurement. Concomitant medication will be documented as per standard NHS care and any changes recorded in the eCRF.

The biopsy will be performed percutaneously under local anaesthetic with ultrasound guidance. This will be performed by a clinician (Consultant or Clinical Fellow) who is an experienced operator. Tissue equivalent to two full cores will be taken using an 18 Gauge Biopsy Gun Needle and sent for conventional histology reporting by the Trial Pathologist and exploratory testing by the TRIG research laboratory.

The participant will be on bed-rest with regular observations for 6-hours post biopsy, as per local Oxford Transplant Centre protocol. Any change in clinical state or suggestion of bleeding will be treated as an emergency, with further management according to local and national guidance. If the patient is haemodynamically stable, pain-free with no evidence of macroscopic haematuria after 6-hours they will be discharged home.

If the renal biopsy demonstrates no evidence of rejection, the renal function remains stable and the trough tacrolimus level remains ≥5 ng/mL the patient will be instructed to reduce the MMF dose to 250mg BD. If the trough tacrolimus level is <5 ng/mL, the MMF will continue at 500 mg BD, and the tacrolimus dose will be adjusted by the patient's nephrologist, with a repeat trough level 3–5 days later (as per standard clinical practice). This step will be repeated until the tacrolimus level reaches ≥5 ng/mL, and only then will the MMF be reduced.

10.10. Visit 8 (both arms)

This visit coincides with a standard NHS follow up visit that takes place at approximately week 24 post-operatively. Visit 8 is a standard clinical visit as per standard NHS care for clinical assessment that coincides with an immune monitoring visit where trial-specific immune monitoring blood samples will be taken as per *Tables 1, 2 and 3*. NHS standard care tests will include blood U&Es, FBC, LFTs, bone profile, glucose, tacrolimus level and urinary protein:creatinine ratio. Viral serology will be checked in accordance with standard clinical practice for CMV and BK virus. A physical examination will be performed to include vital signs and BMI measurement. Concomitant medication will be documented as per standard NHS care and any changes recorded in the eCRF. Patients in both arms will be asked to complete an EQ-5D-5L and SF-36 quality of life questionnaire to be completed whilst they are alone.

10.11. Visit 9 (Treg arm only)

This visit takes place at approximately week 26 post-operatively. Blood procured at V1 is transferred in a sealed container under HTA licensing to GSTT via NHS Blood Bikes where the ATIMP is manufactured according to the IMPD. The product remains cryopreserved at GSTT until required at V9. One week before V9, confirmation will be obtained from the GSTT GMP Unit (GSTT GMP) that the Treg cell product is available and that no delay in delivery is anticipated. The cryopreserved Treg cell product will be delivered from the GMP facility to the Transplant ward where it will be stored in a locked ward drug room only when the relevant release criteria for the product have been met. This will be at least 24 hours before the due infusion date and time. The Treg product will remain cryopreserved throughout all

© Copyright: The University of Oxford and Oxford University Hospitals NHS Foundation Trust 2016

of the delivery steps. Delivery to the Oxford Transplant Centre will be by specialist courier in a time and temperature tracked container.

The patient will be instructed to stop MMF 72 hours before the scheduled appointment for visit 9. Study specific blood samples must be collected before Treg infusion, as stated in *Table 2*. CMV and BK viral titres from Visit 8 will be reviewed and causes of an elevated CRP or leucocytosis will be examined as possible contraindications to Treg infusion. If these causes are reversible (e.g. acute upper respiratory tract infection), then a second attempt at Treg infusion will take place 1-2 weeks later.

Monitoring of the patient will be started, including; ECG, blood pressure, respiratory rate, SaO_2 on air and tympanic temperature. A pregnancy test will be performed prior to cell infusion. Blood will be taken for research for standard panels (FBC, U&E, LFTs, bone profile, lipid profile, CRP, glucose, tacrolimus levels, DSAs) and urinary protein:creatinine ratio as well as immune monitoring panels.

The patient will receive a single prophylactic dose of low molecular weight heparin , dosage adjusted to renal function and weight as per local policy. They will also receive a prophylactic dose of paracetamol 1g PO to mitigate possible febrile reactions to Treg infusion. Prophylactic treatment with an antihistamine, chlorpheniramine 4mg PO, will be given to prevent possible urticaria or pruritus.

The clinician responsible for the infusion will cross-check that the recipient is the intended recipient of the Treg cell product by comparing the 3-point ID labelling of the cell product and the patient's ID wrist band. The cell product will be thawed at the bedside by appropriately trained staff and delivered by syringe into 100 mL 4.5-5% sterile isotonic human albumin solution ((HAS), Human Albumin Biotest 5%, PL 04500/0011) in a glass bottle. The final dispersed cell product will then be infused intravenously through a standard giving set with a 200 µm filter by an infusion pump by a qualified medical practitioner. The infusion rate will be 2-5 ml/min. During the infusion, the clinical condition of the recipient will be observed closely. The infusion rate will be slowed if the patient's temperature rises by >1°C or the patient appears flushed, or if the patient develops pruritus or urticaria. The full volume of the cell product should be infused to the recipient, after which the transfusion bag will be flushed with sterile HAS to ensure full cell delivery. When the infusion is complete, all lines should be detached from the infusion set according to normal routine practice.

Close monitoring of the recipient should continue for 6 hours from the start of the infusion. Particular attention will be paid to possible respiratory complications arising from cardiopulmonary fluid overload, pulmonary embolism or transfusion-associated acute lung injury. At 1 hour post-infusion observations will be recorded in the eCRF. In addition, blood will be taken for FBC, U&E, LFTs, bone profile and CRP. A repeat 12 lead ECG will be taken. 4 hours post-infusion the patients observations and a 12 lead ECG will be taken and results recorded in the eCRF.

An episode of biopsy-confirmed rejection in the first 6 months post transplantation will not be a contraindication to Treg administration providing that (1) the renal function is stable, and (2) the responsible clinician and principal investigator considers it safe and appropriate to proceed. This will be reviewed on a case-by-case basis.

© Copyright: The University of Oxford and Oxford University Hospitals NHS Foundation Trust 2016

10.12. Visits 10 and 11 (Treg arm only)

These visits take place at approximately weeks 27 and 28 post-operatively, respectively. Visits 10 and 11 are additional safety and clinical assessment visits to assess for any changes in renal function or clinical markers that may be associated with Treg therapy as per *Table 1*. Blood will be taken for research for standard panels (FBC, U&E, LFTs, bone profile, CRP, glucose, tacrolimus levels), urinary protein:creatinine ratio, as well as immune monitoring blood tests per *Tables 2 and 3*. A physical examination will be performed to include vital signs and BMI measurement. Concomitant medication will be documented as per standard NHS care and any changes recorded in the eCRF.

10.13. Visit 12 (both arms)

This visit coincides with a standard NHS follow up visit that takes place at approximately 30 weeks postoperatively. This is a standard clinical visit for clinical assessment as per standard NHS care that coincides with an immune monitoring visit where trial-specific immune monitoring blood samples will be taken as per *Tables 1, 2 and 3*. NHS standard care tests will include blood U&Es, FBC, LFTs, bone profile, glucose, tacrolimus level, and urinary protein:creatinine ratio. A physical examination will be performed to include vital signs and BMI measurement. Concomitant medication will be documented as per standard NHS care and any changes recorded in the eCRF.

10.14. Visit 13 (both arms)

This visit coincides with a standard NHS follow up visit. This visit takes place approximately 38 weeks post-operatively. Patients will be admitted to the Renal Day-Case Unit for a protocol biopsy. The biopsy will be performed using a standard technique under local anaesthesia by an experienced clinician. Tissue equivalent to two full cores will be taken using an 18 Gauge Biopsy Gun Needle and sent for conventional histology reporting by the Trial Pathologistand exploratory immune monitoring by the TRIG research laboratory. Results of the conventional histology reported by the Trial Pathologist from this biopsy will be used to guide a reduction in tacrolimus in the Treg therapy arm at Visit 14. Histological assessment will also guide standard clinical treatment as per NHS standard of care. Standard blood tests will be taken for clinical assessment as well as immune monitoring blood tests. A physical examination will be performed to include vital signs and BMI measurement. NHS standard care tests will include blood U&Es, FBC, LFTs, bone profile, glucose, tacrolimus level, coagulation screen, G&S and urinary protein:creatinine ratio. Concomitant medication will be documented as per standard NHS care and any changes recorded in the eCRF.

10.15. Visit 14 (both arms)

This visit coincides with a standard NHS follow up visit that takes place approximately 40 weeks post-operatively. Biopsy results from V13 will be provided to both arms. In the Treg therapy arm only, if the renal biopsy performed at visit 13 demonstrates no evidence of rejection and the renal function remains stable, the tacrolimus dose will be adjusted to achieve whole blood trough tacrolimus levels of 4-6 ng/mL. A physical examination will be performed to include vital signs and BMI measurement. NHS standard care tests will include blood U&Es, FBC, LFTs, bone profile, glucose, tacrolimus level, DSA and urinary protein:creatinine ratio. Concomitant medication will be documented as per standard NHS care and any changes recorded in the eCRF.

© Copyright: The University of Oxford and Oxford University Hospitals NHS Foundation Trust 2016

10.16. Visit 15 (Treg arm only)

This visit takes place approximately 41 weeks postoperatively. The visit is a clinical assessment after tacrolimus reduction in the Treg therapy arm. Blood will be taken for research for standard panels (FBC, U&E, LFTs, bone profile, glucose, tacrolimus levels, DSAs) and urinary protein:creatinine ratio, with no immune monitoring blood tests. A physical examination will be performed to include vital signs and BMI measurement. Concomitant medication will be documented as per standard NHS care and any changes recorded in the eCRF.

10.17. Visit 16 (both arms)

This visit coincides with a standard NHS follow up visit that takes place approximately 42 weeks post-operatively. This visit is a clinical assessment for both arms as per standard NHS care. NHS standard care tests will include blood U&Es, FBC, LFTs, bone profile, glucose, tacrolimus level, and urinary protein:creatinine ratio, with no immune monitoring blood tests. Test results will be shared with the research team. A physical examination will be performed to include vital signs and BMI measurement. Concomitant medication will be documented as per standard NHS care and any changes recorded in the eCRF.

10.18. Visits 17-19 (both arms)

These visits coincide with standard NHS follow up visits that takes place at approximately 44, 52 and 78 weeks post-transplant, respectively. These are visits for clinical assessment as per standard NHS care that coincide with immune monitoring visits where trial-specific immune monitoring blood samples will be taken as per *Tables 1, 2 and 3*. NHS standard care tests will include blood U&Es, FBC, LFTs, bone profile, glucose, tacrolimus level, and urinary protein:creatinine ratio. At visit 19 blood DSA will also be measured as per standard NHS care. A physical examination will be performed to include vital signs and BMI measurement. Concomitant medication will be documented as per standard NHS care at each visit and any changes recorded in the eCRF. Patients in both arms will be asked to complete an EQ-5D-5L and SF-36 quality of life questionnaire at visits 18 and 19 that will be completed whilst the patient is alone.

10.19. Visits 20-23 (both arms)

These visits coincide with standard NHS follow up visits that take place at approximately 104, 156, 208 and 260 weeks post-transplant, respectively. These are visits for clinical assessment as per standard NHS care. NHS standard care tests will include blood U&Es, FBC, LFTs, bone profile, glucose, tacrolimus level, and urinary protein:creatinine ratio and DSA. A physical examination will be performed to include vital signs and BMI measurement. Concomitant medication will be documented as per standard NHS care at each visit. Continued monitoring for adverse events including the development of malignancy will occur.

10.20. Unscheduled visits

Additional visits will be for episodes of rejection requiring biopsies, clinical assessment, clinical blood tests, immune monitoring blood tests, and adjustment of immunosuppression as per Oxford Transplant Centre Standard NHS Protocol and as advised by the patient's Nephrologist.

In the event of a suspected clinical rejection episode a '*for cause*' transplant biopsy will be taken as part of standard clinical care. The decision to perform a 'for cause' biopsy is taken at consultant nephrologist

© Copyright: The University of Oxford and Oxford University Hospitals NHS Foundation Trust 2016

level. Additional exploratory immune monitoring trial tests would be taken at the time of this biopsy and at standard clinic review appointments 1 and 2 weeks later if a rejection episode was confirmed on the transplant biopsy. A physical examination will be performed to include vital signs and BMI measurement. Blood tests and assessments taken at unscheduled visits will be as per standard NHS care and will include blood U&Es, FBC, LFTs, bone profile, lipid profile, glucose, CRP, tacrolimus level, DSA, coagulation screen, and urinary protein:creatinine ratio. Immune monitoring assays will include blood gene expression, leukocyte/serum profiling, and functional assays.

An Unscheduled Visit CRF will be used to gather and enter information contemporaneously at unscheduled visits. Results of clinical blood and urine tests and histological material would be shared with the Trial team.

© Copyright: The University of Oxford and Oxford University Hospitals NHS Foundation Trust 2016

10.21. Trial Flow-Chart

Table 1. Schedule of all recipient trial visits in the TWO Study: Treg Cell Therapy Trial

ASSESSMENTS	V0 Pre-visit	V1 W-3 - 6	V2 W-2 ±1W	Op D0	V3 W1 ±3D	V4 W4 ±1W	V5 W12 ±1W	V6 W14 ±1W	V7 W22 ±1W	V8 W24 ±1W	V9 W26 ±1W	V10 W27 ±1W	V11 W28 ±1W	V12 W30 ±1W	V13 W38 ±1W	V14 W40 ±1W	V15 W41 ±1W	V16 W42 ±1W	V17 W44 ±1W	V18 W52 ±1W	V19 W78 ±1W	V20 W104 ±1M	V21 W156 ±1M	V22 W208 ±1M	V23 W260 ±1M
**STUDY GROUP**																									
Control	X	-	X	X	X	X	X	X	-	X	-	-	-	X	X	X	-	X	X	X	X	X	X	X	X
Intervention	X	X	X	X	X	X	X	X	X	X	X	X	X	X	X	X	X	X	X	X	X	X	X	X	X
**TRIAL ADMINISTRATION**																									
Eligibility check list[a]	X			X																					
Taking informed consent	X																								
Patient trial identifier	X																								
Patient randomisation	X																								
Baseline characteristics	X		X																						
**Rx ADMINISTRATION**																									
Treg/Cell isolation[b]		X																							
TR001 (IMP) administration											X														
Infusion monitoring[c]											X														
MMF initiation				X																					
MMF cessation[d]											X														
Tacrolimus initiation				X																					
Alemtuzumab				X																					
Methylprednisolone				X																					
**ASSESSMENTS**																									
Vital signs		X	X	X	X	X	X	X	X	X	X	X	X	X	X	X	X	X	X	X	X	X	X	X	X
BMI			X	X	X	X	X	X	X	X	X	X	X	X	X	X	X	X	X	X	X	X	X	X	X
Physical examination			X	X	X	X	X	X	X	X	X	X	X	X	X	X	X	X	X	X	X	X	X	X	X
Concomitant medication	X		X	X	X	X	X	X	X	X	X	X	X	X	X	X	X	X	X	X	X	X	X	X	X
12-lead ECG			X								X														

© Copyright: The University of Oxford and Oxford University Hospitals NHS Foundation Trust 2016

EQ-5D-5L and SF-36 QOL questionnaire	X									X										X	X				
**ONGOING**																									
Drug dose adjustments<br>Rejection episode<br>For-cause graft biopsy<br>Dialysis episodes<br>SAE reporting	← AS NECESSARY →																								
**CLINICAL TESTS (See Table 2 for specific details)**																									
Clinical blood tests[e]			X	X	X	X	X	X		X				X	X	X		X	X	X	X	X	X	X	X
Clinical urine test[e]			X	X	X	X	X	X	X	X	X	X	X	X	X	X	X	X	X	X	X	X	X	X	X
Pregnancy test[f]			X	X							X														
Donor-Specific Antibodies			X				X		X		X					X					X	X	X	X	X
Viral load[g]	← As per local clinical policy →																								
Renal biopsy[c]									X						X										
**ADDITIONAL RESEARCH BLOOD TESTS (See Table 2 for specific details)**																									
Standard panel blood tests									X		X	X	X				X								
**IMMUNE MONITORING**																									
Gene expression			X			X	X		X	X	X	X	X	X	X				X	X	X				
Leukocyte/serum profiling			X			X	X		X	X	X	X	X	X	X				X	X	X				
Functional assays			X				X							X							X				
HLA-DR									X		X	X	X												
**BLOOD REQUIRED (mL)**		359	64	23	16	44	71	16	39	44	39	39	39	64	21	21	16	16	39	39	64	21	21	21	21

D = day(s); W = week(s); M = month(s).

[a] Required for the patient no more than 30 days prior to the cell isolation visit

[b] The patient may undergo venesection of 370 mL on a maximum of two occasions separated by a minimum period of 2 weeks. If a second cell isolation visit is required, the patient will not need a new patient identifier.

© Copyright: The University of Oxford and Oxford University Hospitals NHS Foundation Trust 2016

[c] Admission to the Oxford Renal Day Case Unit; pre-treatment with 1g paracetamol and 4mg chlorpheniramine PO; vital signs to be monitored every 15 minutes during the infusion and for one hour after. Thereafter vital signs to be measured hourly for a further 3 hours.

[d] Only for patients randomised to cell therapy arm

[e] Refer to table 2 (schedule of clinical follow-up assessments) for a comprehensive list of all the blood and urine tests required

[f] Only for female patients of child-bearing potential

[g] Viral testing will occur as per transplant centre policy as part of routine clinical care. Surplus blood from these routine clinical tests may be transferred to the University of Oxford and undergo viral sequencing. Additional samples may be taken at other time points depending on clinical indication. Current screening policy includes: CMV PCR testing at approximately 2, 4, 8 and 12 weeks after transplant (or from date of 12-week CMV prophylaxis cessation, if given); BKV PCR testing at 2, 4, 6, 8, 12 weeks, then at 4, 6, 8, 10, and 12 months after transplant.

Visits highlighted in red are additional visits for patients in the Treg therapy arm.

© Copyright: The University of Oxford and Oxford University Hospitals NHS Foundation Trust 2016

Table 2. Schedule of recipient clinical assessments

CLINICAL PARAMETERS	V1 W-6	V2 W-2 ±1W	Op D0	V3 W1 ±3D	V4 W4 ±1W	V5 W12 ±1W	V6 W14 ±1W	V7 W22 ±1W	V8 W24 ±1W	V9 W26 ± 1W		V10 W27 ±1W	V11 W28 ±1W	V12 W30 ±1W	V13 W38 ±1W	V14 W40 ±1W	V15 W41 ±1W	V16 W42 ±1W	V17 W44 ±1W	V18 W52 ±1W	V19 W78 ±1W	V20 W104 ±1M	V21 W156 ±1M	V22 W208 ±1M	V23 W260 ±1M
										Pre-dose	Post-dose														
Additional Treg arm visits	X							X		X		X	X				X								
U&Es[a]		X	X	X	X	X	X	X	X	X	X	X	X	X	X	X	X	X	X	X	X	X	X	X	X
FBC[b]		X	X	X	X	X	X	X	X	X	X	X	X	X	X	X	X	X	X	X	X	X	X	X	X
LFTs[c]		X	X	X	X	X	X	X	X	X	X	X	X	X	X	X	X	X	X	X	X	X	X	X	X
Bone profile[d]		X	X	X	X	X	X	X	X	X	X	X	X	X	X	X	X	X	X	X	X	X	X	X	X
Lipid profile[e]		X								X										X	X	X	X	X	X
CRP		X			X					X	X	X	X												
Glucose		X	X	X	X	X	X	X	X	X		X	X	X	X	X	X	X	X	X	X	X	X	X	X
Tacrolimus level				X	X	X	X	X	X	X		X	X	X	X	X	X	X	X	X	X	X	X	X	X
Coagulation[f]			X					X							X										
DSA		X				X		X		X						X					X	X	X	X	X
Viral serology[g]	X								X																
G&S			X					X							X										
Prot:Creat[h]	X	X		X	X	X	X	X	X	X		X	X	X	X	X	X	X	X	X	X	X	X	X	X
Baseline characteristics		X																							
Vital signs[i]	X[i]	X[i]	X[i]	X[i]	X[i]	X[i]	X[i]	X[i]	X[i]	X[ii]	X[ii]	X[i]	X[i]	X[i]	X[i]	X[i]	X[i]	X[i]	X[i]	X[i]	X[i]	X[i]	X[i]	X[i]	X[i]
BMI[k]		X		X	X	X	X	X	X	X		X	X	X	X	X	X	X	X	X	X	X	X	X	X
Physical examination		X	X	X	X	X	X	X	X	X	X	X	X	X	X	X	X	X	X	X	X	X	X	X	X
Renal biopsy[l]								X							X										

The matrix outlines the correct panel of clinical assessments to be performed at each trial visit. A pregnancy test or a *for-cause* biopsy may be required at any point during the follow-up period. M = month(s). W = week(s). D = Days.

[a] [Na^+], [K^+], [Urea], [Creatinine]
[b] Red blood cells, haemoglobin, haematocrit, mean cell volume, white blood cells (with differential including neutrophils, lymphocytes, eosinophils, basophils, monocytes), platelets
[c] Bilirubin, albumin, aminotransferases and alkaline phosphatase
[d] [Ca^{2+}], [PO_4^{3-}]
[e] Cholesterol, triglycerides, LDL-cholesterol and HDL-cholesterol
[f] Prothrombin Time (PT) and Activated Partial Thromboplastin Time (APTT)
[g] HBsAg, HBcAb, HCV Ab, HIV 1/2 Ab, VZV Ab, HTLV 1/2 Ab, syphilis Ab (unless already known positive; see section 10.3)
[h] Spot urinary protein:creatinine ratio
[i] Heart rate, blood pressure, and temperature measurements. Heart rate and blood pressure should be measured after 5 minutes rest in the semi-recumbent position
[ii] Heart rate, blood pressure, temperature measurements, respiratory rate, SaO_2 on air
[k] Patient mass (kg) and height (m) should be measured to calculate BMI; formula = mass/(height)^2

© Copyright: The University of Oxford and Oxford University Hospitals NHS Foundation Trust 2016

11. Sample Handling

Clinical laboratory tests will be performed on blood samples collected at each trial visit and on urine samples collected as per Table 2. The laboratory assessments contained in this protocol comprise routine haematology, biochemistry and urinalysis for kidney transplant recipients. All samples taken for clinical follow-up will be processed in the local clinical chemistry laboratories (based at the Churchill and John Radcliffe Hospitals, Oxford). These tests are part of routine NHS care of a recent transplant recipient with standard haematological and biochemical measurements of blood and urine. Samples are destroyed after analysis as per Trust standards. The results of these routine tests will be accessed by the research team. Visits 1, 7, 9-11 and 15 are extra visits as part of the treatment arm of the TWO Study trial and whilst the same haematological and biochemical markers will be tested in the pathology laboratories of the Oxford University Hospital, the samples are taken for research purposes and destroyed after analysis.

The blood volume taken at each visit for routine biochemical and haematological testing will be approximately 16 mL. Visits requiring the extra clinical bloods for research will increase the blood volume required as follows: coagulation studies (2.7 mL), DSA (5 mL), Group & Save (G&S; 4 mL) and viral serology (9 mL). The total additional blood taken for research purposes at specific visits varies from 23-48ml for immune monitoring as outlined in detail in Table 1.

During the cell procurement visit (pre-transplant), a total of approximately 370ml of whole blood will be collected for Treg isolation and expansion (intervention arm only). Raw consumable materials used for blood procurement will be stored at ambient temperature (15 – 25 °C as per manufacturer instructions) in a locked storage area of the OTC. A daily temperature log will be maintained in this storage area for quality assurance purposes. Temperature excursions beyond the recommended limits will result in the disposal and replacement of such materials.

Immune monitoring samples will be taken as per Section 14.6. Samples will be analysed on receipt in TRIG laboratories or batch analysed during the course of the Trial. Samples not analysed during the course of the trial will either be destroyed or transferred to an HTA-approved facility for later analysis.

EDTA samples will be collected as per local clinical policy for viral screening and monitoring (PCR). The remnants of these samples (after clinically indicated PCR testing is performed) will be collected from the clinical microbiology laboratory and transferred to University of Oxford. Where detectable virus (primarily CMV and BKV) is identified, these samples will be used for extraction of viral DNA and viral next generation sequencing. Results will be correlated to immunophenotyping of the patient, performed as part of The TWO Study. No additional sampling will be required beyond those already being taken for routine viral monitoring. Any samples not analysed during the course of the trial will be destroyed.

12. Measurement of Glomerular Filtration Rate and Integrity

For the purposes of this study, creatinine clearance (CrCl) will be taken as an approximation to GFR. To avoid the need for 24-hour urine collection, CrCl will be estimated from serum creatinine values. Several formulae to estimate GFR exist, have been tested in renal transplant recipients, but none has been

© Copyright: The University of Oxford and Oxford University Hospitals NHS Foundation Trust 2016

shown to be consistently superior to others. When creatinine is measured during the study, the electronic database will automatically calculate eGFR using CKD-EPI.

For all equations given below, serum creatinine (SCr) is in mg/dL, mass in in kilograms (kg), age is in years.

- **CKD-EPI: eGFR =**
  $141 \times \min(SCr/\kappa,1)^{\alpha} \times \max(SCr/\kappa,1)^{-1.209} \times 0.993^{Age} \times 1.018$ [if female] $\times 1.159$ [if black]

  $\alpha = -0.411$ for males; -0.329 for females

  $\kappa = 0.9$ for males; 0.7 for females

Glomerular integrity: Impairment of glomerular integrity is manifest as proteinuria, which will be assessed by formal laboratory measurement (urinary protein: creatinine ratio).

13. Kidney Graft Biopsies

Tissue biopsies of the transplanted allograft will be taken as either scheduled (protocol biopsies) or unscheduled (*for-cause* biopsies). Protocol biopsies are required at Visit 7, week 22 (intervention group only) and Visit 13, week 38 (both groups) in order to facilitate the decision to reduce immunosuppression.

If a kidney transplant biopsy (protocol) is performed and demonstrates signs of antibody-mediated rejection, a serum sample will be collected for donor-specific antibody (DSA) testing, as per standard clinical practice. The serum sample will be processed locally by the clinically accredited tissue-typing laboratory. Additional blood samples will be taken for immune monitoring as per *Table 3.*

13.1. For-cause biopsies

For-cause biopsies are standard of care procedures and will be taken by the direct care team as clinically necessary to obtain histological confirmation of a clinical acute rejection episode, or to confirm resolution of a rejection episode. For-cause biopsies may be taken at any time after transplantation as part of standard NHS care, independently of the trial. Whenever rejection is suspected, a for-cause graft biopsy will always be offered and performed with the patient’s permission. The decision to proceed to biopsy will be taken by a consultant nephrologist taking into account the full clinical picture as per standard NHS practice. The results of the for-cause biopsies will be available to the trial investigators and the outcome will be documented in the electronic database. Whenever a for-cause biopsy is reported as *suspicious for rejection* on the basis of borderline changes, responsibility for a diagnosis of rejection (and the patient registering a primary end-point) lies with the treating physician.

13.2. Biopsy material

The allograft will be biopsied under local anaesthetic with real-time ultrasound guidance. Tissue equivalent to two full cores will be taken using an 18 Gauge Biopsy Gun Needle Protocol biopsies will be sent for histopathology assessment and immune monitoring purposes. These biopsies will be anonymised with a unique biopsy identifier which will be linked to the patient identifier and sent for processing by Oxford Centre for Histopathology Reseach (OCHRe) and then stored in the NDS TRIG

© Copyright: The University of Oxford and Oxford University Hospitals NHS Foundation Trust 2016

laboratory once histopathology assessment is complete. Samples will be anonymised and used for immune phenotyping, which may include assessment of cellular infiltration with Tregs by TCR sequencing, single cell sequencing, immunohistochemistry, andgene signature by transcriptomics, microarrays or qPCR. For cause biopsies taken as part of standard NHS care will be sent as usual for analysis in the Department of Cellular Pathology, John Radcliffe Hospital.

Histopathology should be used to guide the clinical management of the patient, and the analysis recorded in the electronic database. Histopathological grading performed by Professor Ian Robert's team will be used as the official result for the trial. Additional staining of the biopsy material to aid interpretation by Prof. Roberts will be undertaken by the Oxford Centre for Histopathology Research (OCHRe), based at the John Radcliffe Hospital, Oxford.

Once reviewed, for cause histology slides and specimens will be stored in a secure clinical archive at the Department of Cellular Pathology at the John Radcliffe Hospital, for at least 30 years (consistent with HTA requirements and as per standard local practice according to Royal College of Pathologists (UK) guidance).. Only authorised histopathology staff will have access to the material, but the samples may be made available for future reference to clinicians depending on clinical need, or for future ethically-approved studies. Protocol biopsy samples that remain unanalysed at the expiry of ethics approval may be destroyed or transferred to an HTA approved facility for future research.

13.3. Severity of rejection episodes

In addition to histological grading, acute T-cell mediated rejection episodes will also be classified according to their response to treatment as:

- ***Spontaneously resolving***: An episode of clinically manifest rejection which resolves without new or increased corticosteroid treatment, irrespective of any change in tacrolimus or mycophenolate dose
- ***Glucocorticoid-responsive***: An episode of clinically manifest rejection which resolves following administration of new or increased doses of corticosteroid treatment, irrespective of any change in tacrolimus or mycophenolate dose
- ***Responsive to depleting antibody treatment***: An episode of clinically manifest rejection which does not resolve following administration of new or increased doses of corticosteroid treatment, but is responsive to treatment with lympho-depletive antibody therapy. Episodes of rejection initially treated with lympho-depletive antibody therapy will always fall into this category.

14. Immune Monitoring (IM)

Immune monitoring measures functional and molecular correlates of immune reactivity. As clinical transplantation attempts to move from empirical immunosuppressive dosing towards individually-tailored therapy, a range of laboratory assays have been developed that might guide clinical decision-making. Although many of these assays remain experimental, accumulating evidence suggests the potential for these tools to guide clinical immunosuppressive dosing (Ashton-Chess, Giral, Soulillou, & Brouard, 2009). Some of the assays to be used have been developed from data obtained in the ONE Study, the Phase I trial preceding the TWO Study.

Immune monitoring will provide an opportunity to obtain insights into the action of regulatory cell

© Copyright: The University of Oxford and Oxford University Hospitals NHS Foundation Trust 2016

populations *in vivo* in the treatment arm compared with the control arm of this study. Data generated by these assays will not be used to guide clinical management of patients in the trial. The specific samples taken are detailed in Table 3. All samples will be anonymised and given a participant number before transfer to NDS TRIG laboratories for analysis or storage and later bulk analysis. Samples not analysed within the Trial timeframe will be transferred to an HTA-approved facility. Access to samples will only be by the trial team. Any samples that are not analysed or excess to requirements for the analyses below will be stored for later analysis in future ethically-approved studies. Sample analysis will be performed in the University of Oxford. Anonymous raw immunophenotyping data will be analysed in Oxford and, with the input of collaborators. T cell receptor sequencing of blood or biopsy material may be performed by an external company.

14.1. Additional Blood Collection Time Points

a. Donor: Additional bloods for immune monitoring will be taken from the donor two weeks before kidney donation at V2 in parallel to a standard pre-donation clinical visit.
b. Kidney Transplant Recipients:
   - Immune monitoring (gene expression/leucocyte and serum profiling blood samples will be taken at V2; V4; V5; V8; V12; V13; V17; V18; V19 in both control and treatment arms. Additional immune monitoring tests will be taken in the treatment arm at V7; V9; V10; V11.
   - Functional Assays will be taken at V2; V5; V12 and V19 in both arms of the trial.

14.2. Objectives of IM

1. Determine the immune status of organ recipients and measure reactivity against donor alloantigen at multiple time points post-transplant

2. Review for evidence of harm or benefit cause by administering cell therapy

14.3. Design of the IM component of the study

The exploratory immune-monitoring assays have been selected to examine key phenotypic and functional changes and to look at post-transplant biomarkers that may indicate the safety, efficacy, or mode of action of the cell product.

The following subsections briefly outline the scientific rationale for each IM assay. The assays are lettered A-F, for cross-referencing with Table 3. See *Section 19.3* for details on the IM database.

14.4. Measures of Safety

The safety aspect of the immune-monitoring may help identify the susceptibility of patients to infection. Taken at pre-defined time points, viral load measurements will provide information on the CMV, EBV, and BK virus burden of each patient over time. Viral sequencing will detail any changes in the virus related to TR001 infusion and changes in the immune constitution. HLA-DR protein quantification may give insight into innate immune responses and hence susceptibility to bacterial/fungal infection. Both assays will be performed locally – viral load will be measured as per routine clinical practice; HLA-DR will be quantified by flow cytometry at the NDS TRIG Laboratory.

© Copyright: The University of Oxford and Oxford University Hospitals NHS Foundation Trust 2016

A: Absolute quantification of HLA-DR expression by peripheral blood monocytes is a useful and reproducible surrogate marker of innate immune responses. The following ranges have been determined: Normal healthy controls >15,000 molecules per cell; immunodepression 15,000 – 8,000 molecules per cell; immunoparesis <8,000 molecules per cell. HLA-DR quantification by flow cytometry will be performed at the NDS TRIG Laboratories. The results will be uploaded into the electronic trial database, but will not be made available to local clinicians in a timeframe that might influence clinical practice.

14.5. Potential immunological indicators of rejection/unresponsiveness

The TWO Study will assess IM assay results that could (retrospectively) act as surrogate endpoints for transplant outcomes. Certain assays will investigate whether cell therapy shifts kidney transplant recipients towards a more tolerance-prone phenotype or away from a rejection-prone phenotype. Measures of rejection or tolerance will be based on a selection of biomarkers and functional assays. Any classification of individual patients according to biomarker profiling outputs will not be used with the purpose of guiding clinical decision-making. For flow cytometric analyses samples will be analysed immediately at NDS TRIG Laboratories. For gene expression, cytokine expression, T cell functional assays or metabolic profiling assays, samples will be collected, frozen and stored in the Transplantation Research Immunology Group vapour phase nitrogen facilities at the University of Oxford for later batch analysis after full recruitment has completed. Gene analyses may reveal alterations in the immune profile and are unlikely to reveal any previously undiagnosed pathologies. TCR sequencing data will be used to assess for survival of the Treg cell product and not reported in raw form. It is expected that analysis will be performed during the trial duration. Once analysis is complete, samples will be destroyed. If analysis is not complete within the trial duration, samples will be transferred to an HTA-approved facility until fully analysed.

B: Gene expression profiling of a defined set of tolerance-associated genes in whole blood will be profiled by qPCR. Blood samples will be collected in Tempus™ tubes and stored at the trial centre where qPCR tests will be performed and results kept in the immune monitoring database and the Koehler study database.

C: Leucocyte subset profiling using blood leucocyte markers will be performed by flow cytometry to quantify immune cell subpopulations in patient peripheral blood. The NDS TRIG laboratories will analyse samples using a centrally-validated antibody panel on the local flow cytometry instrument.

D: Donor-reactive T-cell frequencies will be measured using a CD154/137 assay performed before and after surgery to enable an estimation of the pre-transplant frequency of donor-reactive T-cells, and detection of post-transplant sensitisation against donor antigen. Patient & donor blood PBMC preparations will be studied in a test that will analyse T-cell activation using the assessment of CD154 (CD40L) vs. CD137 (41BB) expression on T cells after co-culture with allogenic donor antigen presenting cells. The assay enables simultaneous assessment of conventional and natural regulatory T cell reactions towards a defined allogeneic stimulus based on direct alloantigen recognition and will be performed in both arms of the trial. This assay requires blood samples from both the organ recipient and organ donor. After transplantation, samples for the CD154/CD137 assay will be collected at three further visits of the recipients. Only a single procurement from the donor is required. Results will be stored in the immune monitoring database and the Koehler study database.

© Copyright: The University of Oxford and Oxford University Hospitals NHS Foundation Trust 2016

E: Regulatory T-cell frequencies in patient blood will be measured by epigenetic analysis of the Treg-specific demethylated region (TSDR) of the Forkhead box P3 (Foxp3) gene. Results will be uploaded into the IM database (*Section 19.3*) and the Koehler study database.

F: Cytokine and metabolic profiling will be performed assessing a specified set of inflammatory and regulatory cytokines as well as low-molecular-weight metabolites to provide a picture of the dynamic changes that may take place in the immune response after cellular therapy and immunosuppression modification. This assay requires serum which will be obtained from patient blood samples and frozen for later batch analysis. Results will be kept in the immune monitoring database.

14.6. Sampling for immune monitoring assays

Patient sampling

All immune monitoring assays require whole blood as the starting material. Samples for all assays will be collected in tubes pre-labelled with unique identifiers linked to patient identifiers and handled/processed according to dedicated SOPs. All analyses will be undertaken anonymised. Assays measuring T cell function require 40mls blood from the organ donor, taken only once prior to transplantation. For patients in the control arm, immune monitoring visits will always coincide with standard clinical visits. Both groups will attend the following immune monitoring visits that coincide with clinical visits: V2 (pre-transplantation), V4 & V5 (weeks 4 & 12), V8 (week 24), V12 (week 30), V13 (week 38), V17 (week 44), V18 (week 52), and V19 (week 78). Patients in the Treg arm will also attend the following four immune monitoring visits: V7 (week 22), V9 (week 26), V10 (week 27), V11 (week 28). The amount of blood taken from patients at each visit is given in *Table 1*. All samples will be associated with their respective visit numbered entry on the eCRF.

The immune monitoring assays require precise volumes of patient blood treated with specific agents. Particular care will be taken to ensure that the correct type of blood tube and anti-coagulant is used when drawing the different blood samples (*Table 4*). Patient blood will be drawn in the specified quantities. Volumes are per patient per sampling time point. For rejection episodes outside of trial visits, a total of 10 mL of patient blood (2 x 3.0 mL in Tempus™ Tubes for qPCR; 4.0 mL in an EDTA Vacutainer for leucocyte profiling) may be collected, at the discretion of the local Investigator.

© Copyright: The University of Oxford and Oxford University Hospitals NHS Foundation Trust 2016

Table 3. Immune monitoring assays

CODE	ASSAY	TECHNIQUE	TIME	SAMPLE	VOLUME TAKEN AT EACH TIME POINT
**A**	HLA-DR	Flow Cytometry	Pre-Treg infusion and first post-Treg infusion visit	Patient blood	No additional volume required – analysed from leukocyte profiling tube
**B**	Gene Expression	qPCR, RNAseq	All IM visits	Patient blood	6.0 mL
**C**	Leucocyte Profiling	Flow Cytometry	All IM visits	Patient blood	10.0 mL
**D**	T-cell function	CD154/137 Assay	Pre-transplantation and three post-transplantation infusion visits	Patient and donor blood	40mls of donor blood is obtained at visit 2 and used throughout the study for this assay.<br><br>20.0 mL of recipient blood is required for each assay timepoint
**E**	Treg Frequencies	FOXP3 Demethylation	All IM visits	Patient blood	2.0 mL
**F**	Cytokine and metabolic profiling	ELISA, Chromatography-mass spectrometry	All IM visits	Patient blood	5.0 mL

© Copyright: The University of Oxford and Oxford University Hospitals NHS Foundation Trust 2016

Table 4. Sampling and tubes for immune monitoring assays

IMMUNE MONITORING ASSAY	PATIENT BLOOD SAMPLE REQUIREMENTS		
	Volume (per time point)	Aliquots	Blood Container
A. HLA-DR	0 ml*	*	EDTA Vacutainer
B. Gene Expression	6.0 mL	2 x 3.0 mL	Tempus™ Tube
C. Leucocyte Profiling	10.0 mL	1 x 10.0 mL	EDTA Vacutainer
D. T-cell function	20.0 mL	2 x 10.0 mL	Heparin Vacutainer
E. Treg Frequencies	2.0 mL	1 x 2.0 mL	EDTA Vacutainer
F. Cytokine and metabolic profiling	5.0 mL	1 x 5.0 mL	EDTA Vacutainer

* HLA-DR will be performed using samples from the leukocyte profiling tube

15. Discontinuation/Withdrawal of Participants from Trial Treatment

To participate in the study, a recipient must be deemed suitable for enrolment in the trial. Willing subjects will be screened against a list of criteria that must be fulfilled in order for the individual to commence participation.Organ donors may choose to withdraw their research consent at any time. If a donor withdraws consent after IM blood sampling, the donor will be informed that any untested blood belonging to the donor will be destroyed as required. If the recipient withdraws, then the donor will also be withdrawn.

Patients may be presented with options for withdrawal which include **full withdrawal** (no further trial contact, all samples and data already collected to be destroyed and no consent for ongoing or future review of medical records for trial related data), or **partial withdrawal**, where patients withdraw from active trial follow up (i.e trial visits) but the trial team are still permitted to retain and use any data or samples already taken and review medical records. However, in the case of full withdrawal of a patient who has already received Treg therapy, a period of follow up until at least their next two clinical follow up visits will be maintained to assess short-term safety. During this follow up period only access to medical records will be required and no additional research only blood tests are taken. A combination of these two levels of withdrawal may also be agreed with the participant, and documented on the Study Withdrawal CRF.

Each participant may prematurely discontinue their participation in the TWO Study if any of the following occur prior to visit 23:

- Death
- Withdrawal
- Circumstance or event that leads to patient replacement

© Copyright: The University of Oxford and Oxford University Hospitals NHS Foundation Trust 2016

Patients reserve the right to withdraw their consent at any time and for any reason, without penalty or prejudice. Should a patient decide to withdraw, all efforts will be made to discuss and document their reason for withdrawing, where circumstances allow this course of action. The date of patient withdrawal, and any missing items of data (up to the point of patient withdrawal), should be entered into the relevant electronic database pages. In the event of patient withdrawal, the reasons (where provided) will be recorded and form part of routine DSMC review.

Patients who prematurely discontinue trial participation cannot reach study completion. For these patients, protocol treatments are no longer obligatory. Standard NHS clinical follow up will continue and any relevant safety, renal function, and rejection episodes will be available to the Investigators only where consent for this ongoing access to medical records was not explicitly withdrawn by the participant at the time of their withdrawal. Following a withdrawal from the trial, the choice of immunosuppression will thereafter be led by the responsible consultant nephrologist, and routine NHS clinical follow-up will continue. All relevant data relating to death, withdrawal or replacement will be documented in the electronic database. Patient withdrawal and patient replacement are discussed in detail in the following sub-sections. In cases of withdrawal due to pregnancy, whenever possible, the pregnancy will be followed-up to term to determine if any SAEs occur.

In addition, the Investigator may discontinue a participant from the trial (or further trial treatment) at any time if they consider it necessary for any reason including:

- An intolerable adverse event
- The need for a major surgical intervention
- Rational concerns for the safety of the patient
- If insufficient cells are generated for cellular therapy after the patient has undergone two blood draws
- Grade 2 Protocol Treatment Non-compliance
- Major non-compliance with the trial visit schedule
- The classification of a patient as ‘lost-to-follow-up’
- Loss of patient mental capacity to make decisions regarding their ongoing care as defined by the Mental Capacity Act 2005 that is permanent or unlikely to be regained.
- Patient pregnancy (planned or actual)

A diagnosis of rejection (clinical and/or histopathological) is not a criterion for withdrawal, despite being a primary endpoint. Patients with allograft rejection should continue trial follow-up, even if their subsequent therapy deviates from the protocol treatment specifications.

The health, safety and well-being of patients will be the highest priority during the conduct of this trial. Whenever the participation of a patient raises valid ethical concerns, SITU will confer with the treating clinician and the outcome will be comprehensively documented.

15.1. Patient Replacement

With a relatively small patient sample size, the emergence of significant numbers of patient discontinuation in the trial may obscure the true outcome of this research. Furthermore, considering the substantial resources that are invested in Treg production and the IM aspect of this study, immune monitoring of discontinued participants would be both scientifically futile and economically wasteful.

© Copyright: The University of Oxford and Oxford University Hospitals NHS Foundation Trust 2016

To compensate for this eventuality, discontinued participants should be replaced by the recruitment of additional patients. The decision to replace individual patients will ultimately be made by the Clinical PI on the basis that some *unanticipated factor* may influence the clinical outcome in terms of the primary endpoint. Thus, potential reasons for patient replacement are diverse and may include, but are not limited to:

- Surgical complications resulting in prolonged cold ischaemia time (>480 minutes for live donors), or necessitating further surgical interventions
- Primary non-function of the allograft and/or graft nephrectomy
- Severe concomitant illness (not related to the immunosuppressive treatment, kidney transplantation, or underlying disease)
- Patient withdrawal
- Patient death prior to administration of the cell therapy product or transplantation
- Contraindication to cell infusion developing during the first 6 months post transplantation
- Inability to expand Tregs sufficiently for intervention

Figure 2 categorises the scenarios that may result in patient replacement. There are no rigid criteria for patient replacement. For example, it will not always be appropriate for a deceased or withdrawn patient to be replaced, as a patient may have registered a legitimate primary endpoint in the trial prior to death, or prior to meeting a withdrawal criterion. Alternatively, in certain circumstances patient replacement would be advisable in the event of a death or withdrawal. For this reason, patient replacement will be assessed on a case-by-case basis.

Figure 2. Diagram of potential scenarios that may lead to patient replacement.

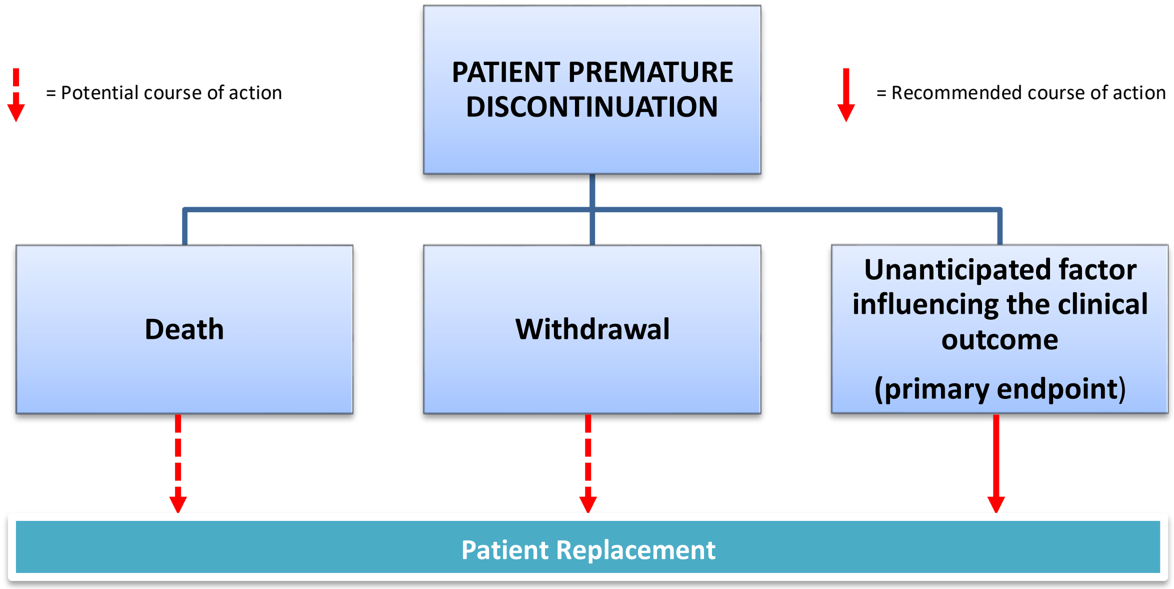

To avoid investigators biasing outcomes through inappropriate replacement of patients, clinicians will be asked to justify each replacement in a written statement to the Data and Safety Monitoring Committee for consideration at the following DSMC meeting.

© Copyright: The University of Oxford and Oxford University Hospitals NHS Foundation Trust 2016

Replaced patients prematurely discontinue their involvement in the study and a new patient may be recruited and randomised in their place.

Data on all patients randomised will be presented.

15.2. Definition of End of Trial

The end of the trial is defined as 'last patient last visit'. This will be the date that the last on-going patient completes the final visit or the date that the last on-going patient prematurely discontinues their participation due to death or withdrawal, or the date that the trial is prematurely terminated in accordance with a trial discontinuation criterion. Upon trial completion, the Sponsor's delegate will give formal notification that the trial has ended to the relevant regulatory bodies within the required timeframes. The CI will inform the REC when the trial ends.

The end of the trial for each individual transplant recipient will be the date that they complete the final trial visit or, if the patient prematurely discontinues participation in the trial, the date of death, withdrawal or replacement. The end of the trial for living kidney donors will be the day of donation on Day 0.

16. TRIAL MEDICATION

Patients in the Treg Cell Therapy Trial will be treated with two standard immunosuppressive drugs plus methylprednisolone and alemtuzumab at induction, all of which will be classed as Non-Investigational Medicinal Products (NIMPs). Treg cellular therapy (an Advanced Therapy Investigational Medicinal Product; ATIMP) will be administered at 6 months, in the patients randomised to cell therapy.

Current standard immunosuppressive therapy for new kidney transplant recipients in the UK is combination therapy with tacrolimus, mycophenolate mofetil and prednisolone following induction with basiliximab. This is largely supported by the results of the European multi-centred Symphony-ELITE Trial, which demonstrated lower acute rejection rates and better outcomes with this combination compared to previous regimens, both at one and three years (Ekberg & group, Reduced exposure to calcineurin inhibitors in renal transplantation., 2007; Ekberg, et al., 2009).

More recently, in an effort to reduce steroid exposure and the associated side-effects, many centres (including Oxford), are using alemtuzumab induction therapy (with single dose corticosteroid), which allows steroid-free maintenance immunosuppression (MMF and tacrolimus), without any increase in acute rejection rates. This practice is principally guided by the 3C study (Group T. 3., 2014).

The use of the immunosuppressive agents (alemtuzumab, tacrolimus, MMF) in this study is consistent with current NICE and RA/BTS guidelines (National Institute for Health and Care Excellence, 2004; Baker, Jardine, & Andrews, 2011), will be prescribed by a doctor and is current 'standard care' in the Oxford Transplant Centre. As such, the immunosuppressive drugs will not require labelling in line with Volume 4, Good Manufacturing Practices, Annex 13, Manufacture of Investigational Medicinal Products, July 2010 (hereafter referred to as Annex 13).

16.1. Drug Regimen (NIMPs)

Figure 3 provides a schematic overview of the medication use in the Treg Cell Therapy Trial.

© Copyright: The University of Oxford and Oxford University Hospitals NHS Foundation Trust 2016

Figure 1. Diagrammatic representation of the immunosuppressive regimen for kidney transplant recipients in the TWO Study

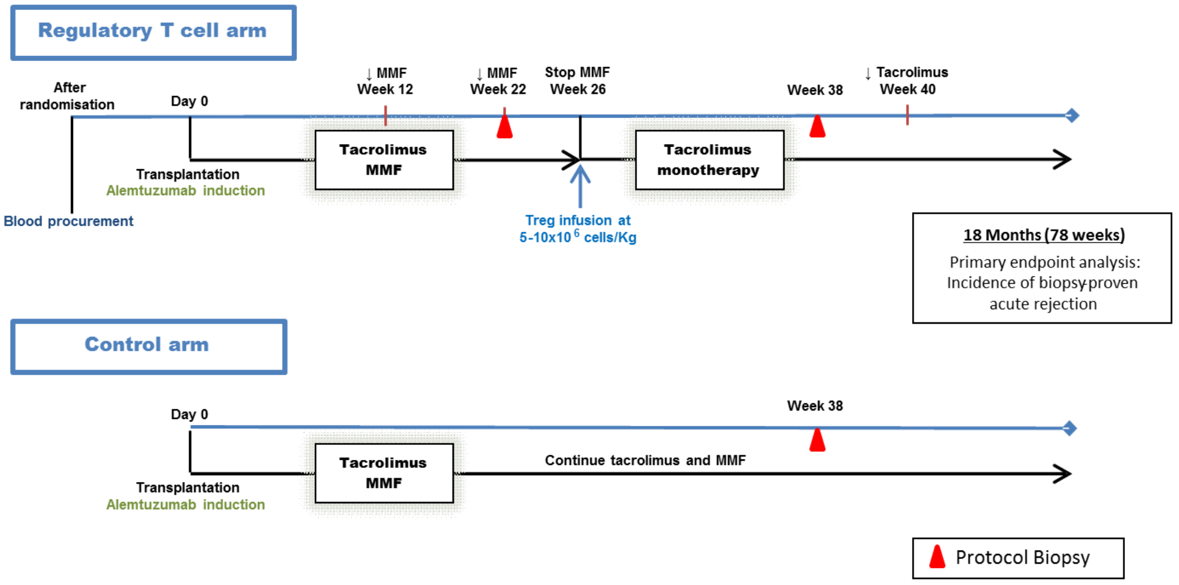

16.2. Monitoring of immunosuppression

NIMPs used in this trial have a marketing authorisation in the EU and a Summary of Medicinal Product Characteristics (SmPC) is referred to for each of the drugs in this section. Drug doses used will be entered into the eCRF at each study visit and/or data obtained retrospectively from medical records where appropriate, for example if doses are altered at non-study visits. Tacrolimus blood levels will be monitored as per standard clinical care and levels similarly entered into the eCRF as per Table 2.

16.3. Corticosteroids

A single dose of 500 mg methylprednisolone will be given intravenously after the transplant operation has started, prior to reperfusion of the allograft with recipient blood.

In the event of a rejection episode, intolerance to a study medication necessitating cessation of the drug, or at the discretion of the treating consultant nephrologist (due to unforeseen circumstances), oral and/or IV corticosteroids may be added to the treatment regimen.

16.4. Alemtuzumab

Alemtuzumab is a humanised monoclonal antibody against CD52, which causes rapid lymphodepletion after administration. It is currently licensed for use in patients with multiple sclerosis, but is available on a named-patient basis for use in transplant recipients. Based on the results of the 3C study it is used widely in transplant centres across the world, is endorsed in RA/BTS guidelines (Baker, Jardine, & Andrews, 2011) and indeed is the *standard of care* locally in the Oxford Transplant Centre (Kidney Transplant Protocol (version 7.0), September 2016, Oxford Transplant Centre).

© Copyright: The University of Oxford and Oxford University Hospitals NHS Foundation Trust 2016

16.5. Tacrolimus

Tacrolimus immunosuppression should be initiated on the day of surgery (D0). Therapy should be adjusted to achieve whole blood drug trough levels that fall within 5-10 ng/mL, for the first 9 months, reducing to 4–6 ng/mL at week 40 (Visit 14) if the week 38 (Visit 13) protocol biopsy shows no evidence of rejection.

Initial dosing will be based on body weight. For immediate release formulations this will be at a dose of 0.05 mg/Kg BD, rounding the dose to nearest 0.5 mg. If the patient is Afro-Caribbean the initial tacrolimus dose will be doubled to 0.1 mg/Kg BD. For once a day prolonged release formulations (Envarsus) initial dosing will be 105 micrograms/kg once daily increased to 175 micrograms/kg once daily for Afro-Caribbean patients.

Tacrolimus will preferentially be prescribed as a prolonged release once a day formulationbut other formulations of tacrolimus are permitted. Once a day prolonged release tacrolimus is not currently the standard formulation in the Oxford Transplant Centre however it is licensed for use in renal transplantation and has been used successfully in patients managed in Oxford previously. This formulation may theoretically be preferable in the context of Treg infusion due to reduced peak serum concentrations. Tacrolimus will be prescribed by brand name as per MHRA guidance.

16.6. Mycophenolate Mofetil (MMF)

After return from theatre (Day 0), the patient will start oral MMF 750mg BD. The MMF dose will be reduced to 500 mg BD in both treatment arms at week 12 (Visit 5). At week 22 (Visit 7), patients in the intervention arm will have a renal biopsy. If this demonstrates no evidence of rejection the patient will be contacted within 48 hours of the procedure, and be instructed to reduce their MMF to 250 mg BD. Patients in the intervention arm will then be instructed to stop their MMF 72 hours prior to the visit 9 study appointment. Patients in the control arm will continue MMF at 500 mg BD. This is summarised in *Table 5.*

Table 5 MMF dosing by treatment arm and time point

Time Point	Treatment arm	
	Control Group	Intervention Group
**D0**	750 mg BD	750 mg BD
**W12 (visit 5)**	500 mg BD	500 mg BD
**W22 (visit 7)**	500 mg BD	250 mg BD
**W26 (visit 9)**	500 mg BD	STOP

Doses of MMF may be modified from that required in response to leucopenia, thrombocytopenia, anaemia or gastrointestinal side effects (e.g. diarrhoea). This will be at the discretion of the treating Consultant Nephrologist. In the event of a reduction in absolute neutrophil count (ANC), the following dose modifications will be made:

© Copyright: The University of Oxford and Oxford University Hospitals NHS Foundation Trust 2016

ANC ($x10^9$/L)	MMF dose modification (max. dose)
≥2	750 mg BD
1.5 – 1.99	500 mg BD
1 – 1.49	250 mg BD
<1	Stop MMF

16.7. Paracetamol

Patients in the Treg arm will receive a prophylactic dose of paracetamol 1g PO to mitigate possible febrile reactions at the time of Treg infusion (V9).

16.8. Chlorpheniramine

Patients in the Treg arm will receive prophylactic treatment with chlorpheniramine 4mg PO to prevent possible urticaria or pruritus at the time of Treg infusion (V9).

16.9. Supply of ATIMP (See also section 10.11 above)

Purified, expanded polyclonal autologous Treg cell products will be produced by the GMP facility, at Guy's & St Thomas' Hospital (GSTT), London, on a per- and named-patient basis and labelled by the GMP facility. For each patient the Treg cell product (designated TR001) will be delivered to the transplant ward at a pre-prepared dose based on patient body weight (10 x 10^6 cells per Kg). A sample of the TR001 product in excess of that required for administration (a Reference sample), will remain cryopreserved at Guys and St Thomas's Hospital for retrospective assessment in the case of suspected unexpected adverse events.

16.10. Labelling of ATIMP

An example of the TR001 product label produced by the GSTT GMP unit that will be used can be seen below.

Figure 3. Sample label for TR001 cell product

© Copyright: The University of Oxford and Oxford University Hospitals NHS Foundation Trust 2016

TWO Study Cell Therapy **Trial Subject ID:**
Patient name: **DOB:**

Trial EudraCT number: 2017-001421-41

TR001 Batch number:

Sponsor: University of Oxford, UK
Investigator: Dr Paul Harden. Oxford transplant Centre, Churchill Hospital, Oxford, OX3 7LE. Telephone: +44 (0) 7540772355

Frozen liquid cell suspension for intravenous administration

1 dose of 4.8 mL, 10 × 10^6 cells dose/kg: ___________

Use before: DD / MMM / YYYY

Store frozen in vapour phase liquid nitrogen

Thaw and use as directed in the Clinical Trial Protocol and SOP

FOR TWO STUDY: Treg CELL THERAPY TRIAL CLINICAL TRIAL USE ONLY **V1.0 4Jan2018**

16.11. Storage of ATIMP

The TWO Study TR001 ATIMP product will be transported to the transplant unit at least one working day prior to the planned administration in a dry shipper validated to maintain <-150°C for up to 11 days. Transport will be by an accredited specialist courier in a specified vehicle. The cell product will be stored in a secure room at the Churchill Hospital in the dry shipper for no more than 10 days. If the ATIMP is not used in this time frame it will be returned via a temperature monitored and logged dry shipper to GSTT for secure storage. Prior to removing the cell product from the dry shipper, an authorised person will check for absence of temperature monitor alarms and download the data-logger information from the dry-shipper. Authorised staff will review the report from the data-logger to ensure that there were no major temperature excursions during transport and storage. This information will be transferred electronically in a spreadsheet format to the GMP Unit manufacturing site as part of the post-use QP certification. Staff from the GMP unit will then verify with authorised staff at the transplant unit (both by telephone and email) that the transport seals can be broken, and that the product can be used.

16.12. Dosage and administration of the ATIMP

Each patient will be pre-treated with 1g paracetamol PO and 4 mg chlorpheniramine PO one hour prior to the cell infusion. Thereafter the patient will receive a TR001 dose based on body weight (5-10 x 10^6 cells per Kg). Once thawed in a water bath, the TR001 product is reconstituted in 4.5-5% human albumin solution (Human Albumin Biotest 5%, PL 04500/0011) to a volume of 100 mL and given intravenously at a rate of 5 mL/min. The infusion is given via a standard giving set with a 200 µm filter.

16.13. Accountability of the ATIMP

A full accountability record will be maintained at the trial site. The product will be administered as a single infusion by a senior consultant or clinical fellow. Any dose reductions or alterations will be recorded and reported in the trial database. A specific paper CRF will be written to record clinician confirmation that that both that the product was suitable for administration and that the recipient was

© Copyright: The University of Oxford and Oxford University Hospitals NHS Foundation Trust 2016

eligible to receive the ATIMP (TR001) on the date of administration, and to ensure that the correct vial is administrated to the correct patient when it arrives in the Transplant Unit.

16.14. Compliance with Trial Treatment

To measure patient compliance with the immunosuppression, trough blood levels for tacrolimus will be measured at each trial visit. Patients obtain their medication via the transplant pharmacy, who will be asked to highlight any significant issues with supply and demand for repeat prescriptions, indicating under-utilisation and potential non-compliance with supplied medication, as per standard NHS care.

16.15. Non-compliance with the immunosuppressive therapy

As far as clinically feasible, all attempts should be made to maintain trial participants on the protocol-specified regimen for the duration of their involvement in the study. Despite best efforts, non-compliance with the protocol treatment specifications may arise at any time. For example, a clinical decision to alter the specified regimen (in response to adverse drug reactions, an intolerable AE, sub-optimal immunosuppression), or patient non-compliance with the prescribed medication, may cause individual patient treatment to deviate beyond the limits defined in this protocol. This protocol will assess the consequences of changes to the protocol-specified treatment in a reactive manner, and classify non-compliance into two grades, based on the severity of the deviation rather than the nature of the initiating event (see definitions below).

16.16. Grade 1 Protocol Treatment Non-Compliance

Grade 1 non-compliance with the protocol-specified treatment should be documented and relayed to the PI . It should not affect trial follow-up and the patient should continue to be assessed according to the visit schedule. Grade 1 non-compliance includes:

- Minor dosing deviation (e.g. missed doses of up to 2 days)
- Minor deviation from the target trough levels for tacrolimus (according to NHS standards)
- Temporary interruption of the protocol treatment regimen (e.g. missed Visits or Visits delayed by up to 2 weeks)
- Minor modification to the protocol treatment regimen (e.g. drug substitution)

Optimal care for all patients enrolled in the TWO Study is paramount. Therefore, the local Investigator reserves the right to modify or interrupt the treatment regimen in cases where strict adherence to the trial treatment protocol might compromise the safety and/or integrity of the patient. It is envisaged that minor dose adjustments and/or transient dosing delays will be sufficient precautionary measures in most circumstances (e.g. for mild AEs, mild ARs). Changes in drug doses should be made with reference to the protocol and the relevant SmPC. Special care should be exercised if adjusting the tacrolimus beyond protocol specifications, due to the time it takes to reach steady state. Changes in the trough level will lag behind dose adjustments; it is recommended that any dose modifications are limited to a maximum of twice per week for tacrolimus.

Where an active clinical intervention results in a Grade 1 non-compliance, the investigators should aim to return the patient to the protocol treatment specifications at the earliest possible time point following patient recovery/stabilisation, providing that no further risks would be associated with such action.

© Copyright: The University of Oxford and Oxford University Hospitals NHS Foundation Trust 2016

In rare cases, an enrolled patient may be intolerant of tacrolimus and require substitution with cyclosporine or an alternative immunosuppressive drug. This type of deviation will normally be considered Grade 1 non-compliance, SITU and the CI should always be informed in the event of any drug substitution.

16.17. Grade 2 Protocol Treatment Non-Compliance

Patients that are Grade 2 non-compliant could be withdrawn from the study (see protocol *Section 15*). Furthermore, these patients may be replaced in the analysis by the recruitment of a new patient (see *Section 15.1*). Grade 2 non-compliance with the protocol-specified treatment includes:

- Persistent and cumulative Grade 1 deviations
- Extended interruption of the protocol treatment regimen
- Substantial modification to the protocol treatment regimen
- Complete cessation of protocol treatment

A treatment interruption/modification due to a diagnosis of rejection for any cause must not be classified as Grade 2 protocol treatment non-compliance. Rejection is an end-point and these patients should continue follow-up and must not be withdrawn from the trial.

Investigators reserve the right to discontinue protocol treatment on the grounds of a clinical decision that is in the best interest of the patient. Ideally, the responsible clinician at the site should discuss a proposed treatment cessation with the clinical PI prior to removing a patient from the protocol-specified regimen. Notes will be written and a decision documented in the site file and TMF.

The clinical PI should always be consulted before a protocol treatment deviation is assigned a Grade 2 classification. The final decision on whether a deviation should be classified as Grade 1 or 2 rests with the PI.

16.18. Accountability of the Trial Treatment

Accountability records will be kept for the ATIMP (see section 16.14). NIMPs will be used within the remit of their licences and/or constitute standard post-operative care of renal transplant recipients. NIMPs are controlled by the OUH Central Pharmacy. The Transplant Unit Pharmacist will control batch numbers and temperature loggers. Prescriptions will be written by clinicians managing the patients in the trial team. OUH Patient Drug Sheets will be used to record and monitor any changes to drugs prescribed. Any changes will also be captured in medical notes and the CRF based on the amended drug charts on EPR.

16.19. Concomitant Medication

Data relating to concomitant therapies will be collected at each trial visit and recorded in the electronic database. Recommended concomitant therapies in the setting of renal transplantation are covered by British Transplant Society (BTS) and UK Renal Association (RA) guidelines. This trial will allow BTS/RA-approved concomitant therapies administered as per the standard practice of the Oxford Transplant Centre. Therapies for concomitant illnesses or pre-existing medical conditions not restricted by trial eligibility criteria are also permitted.

© Copyright: The University of Oxford and Oxford University Hospitals NHS Foundation Trust 2016

16.20. Post-Trial Treatment

As the ATIMP is to be given as a single dose within the trial, there is no provision to supply this beyond the end of the trial. Patients will continue their NIMPs after trial cessation as per standard clinical practice, with prescriptions issued by their attending nephrologist, and dispensed by the transplant pharmacy.

17. SAFETY REPORTING

17.1. Definitions

Adverse Event (AE)	Any untoward medical occurrence in a participant to whom a medicinal product has been administered, including occurrences which are not necessarily caused by or related to that product.
Adverse Reaction (AR)	An untoward and unintended response in a participant to an investigational medicinal product which is related to any dose administered to that participant.<br>The phrase "response to an investigational medicinal product" means that a causal relationship between a trial medication and an AE is at least a reasonable possibility, i.e. the relationship cannot be ruled out.<br>All cases judged by either the reporting medically qualified professional or the Sponsor as having a reasonable suspected causal relationship to the trial medication qualify as adverse reactions.
Serious Adverse Event (SAE)	A serious adverse event is any untoward medical occurrence that:<br>• results in death<br>• is life-threatening<br>• requires inpatient hospitalisation or prolongation of existing hospitalisation<br>• results in persistent or significant disability/incapacity<br>• consists of a congenital anomaly or birth defect.<br>Other 'important medical events' may also be considered serious if they jeopardise the participant or require an intervention to prevent one of the above consequences.<br>NOTE: The term "life-threatening" in the definition of "serious" refers to an event in which the participant was at risk of death at the time of the event; it does not refer to an event which hypothetically might have caused death if it were more severe.
Serious Adverse Reaction (SAR)	An adverse event that is both serious and, in the opinion of the reporting Investigator, believed with reasonable probability to be due to one of the trial treatments, based on the information provided.

© Copyright: The University of Oxford and Oxford University Hospitals NHS Foundation Trust 2016

Suspected Unexpected Serious Adverse Reaction (SUSAR)	A serious adverse reaction, the nature and severity of which is not consistent with the information about the medicinal product in question set out:<br>• in the case of a product with a marketing authorisation, in the summary of product characteristics (SmPC) for that product<br>• in the case of any other investigational medicinal product, in the investigator's brochure (IB) relating to the trial in question.

NB: to avoid confusion or misunderstanding of the difference between the terms "serious" and "severe", the following note of clarification is provided: "Severe" is often used to describe intensity of a specific event, which may be of relatively minor medical significance. "Seriousness" is the regulatory definition supplied above.

Any pregnancy occurring during the clinical trial and the outcome of the pregnancy should be recorded and followed up for congenital abnormality or birth defect, at which point it would fall within the definition of "serious".

17.2. Causality

The relationship of each adverse event to the trial medication must be determined by a medically qualified individual according to the following (binary) definitions:

Related: The adverse event follows a reasonable temporal sequence from trial medication administration. It cannot reasonably be attributed to any other cause.

Not Related: The adverse event is probably produced by the participant's clinical state or by other modes of therapy administered to the participant.

17.3. Adverse Events (AE)

Any non-serious AEs occurring following consenting to participate in the TWO Study by all participants that are observed by the Investigator or reported by the participant that are thought to be related to trial medication (i.e. either the NIMPs or the TR001 product) or trial specified procedures will be recorded on adverse event CRFs. Other non-serious AEs will not be recorded, as they are common in this population and, if not related to study medication, analysis of these events is unlikely to yield useful information regarding the study treatment.

The following information will be recorded: description, date of onset and end date, severity, assessment of causality by trial medication, other suspect drug or device and action taken. Follow-up information should be provided as necessary.

The severity of events will be assessed on the following scale: 1 = mild, 2 = moderate, 3 = severe.

AEs considered related to the trial medication as judged by a medically qualified investigator or the Sponsor will be followed either until resolution, or the event is considered stable or the end of study participation. Standard NHS clinical transplant care continues beyond V23.

© Copyright: The University of Oxford and Oxford University Hospitals NHS Foundation Trust 2016

It will be left to the Investigator's clinical judgment to decide whether or not an AE is of sufficient severity to require the participant's removal from treatment. A participant may also voluntarily withdraw from treatment due to what he or she perceives as an intolerable AE. If either of these occurs, the participant must undergo an end of trial assessment and be given appropriate care under medical supervision until symptoms cease, or the condition becomes stable.

17.4. Reporting Procedures for Serious Adverse Events

The delivery of the Sponsor's responsibility for Pharmacovigilance (as defined in Regulation 5 of the Medicines for Human Use (Clinical Trials) Regulations 2004) has been delegated to the Surgical Interventions Trials Unit (SITU), within the Oxford Clinical Trials Research Unit (OCTRU), Oxford University.

All SAEs occurring during the trial that are observed by the Investigator or reported by the participant, whether or not attributed to trial medication, will be recorded on a paper SAE form. These will be submitted to a secure dedicated TWO-study email address (safety-twostudy@nds.ox.ac.uk) that will be constantly monitored by SITU. SAEs will be captured for each participant for the duration of the trial, which is defined from obtaining informed consent to trial completion at visit 23 (week 260).

All SAEs must be reported on the SAE reporting form to SITU within 24 hours of the trial team becoming aware of the event. Planned hospitalisations as part of standard clinical care and trial visits will not be reported as an SAE, unless there are any untoward medical occurrences. SITU will perform an initial check of the report & request any additional information required. All SAEs will be reviewed centrally by the sponsor's delegate (Nominated person) for expectedness. An independent transplant nephrologist will review all SAEs as part of DSMC review. Additional and further requested information (follow-up or corrections to the original case) will be detailed on a new SAE Report Form.

All SAE Report Forms will be processed by SITU as per the detailed instructions in OCTRU's SOP governing safety reporting for a clinical trial of an IMP (CTIMP).

All SAEs as line listings will be reviewed at the Data and Safety Monitoring Committee (DSMC) meeting.

17.5. Expectedness

Expectedness of SAEs will be assessed according to the current approved Reference Safety Information (RSI). The clinical and non-clinical safety information in the IB has been compiled from the data collected during the preceding ONE study.

Expectedness will be reviewed by the Nominated Person and determined according to the following documents.

Alemtuzumab

Summary of Product Characteristics: Lemtrada, 12mg concentrate for solution for infusion, manufactured by Sanofi (Genzyme), Section 4.8 of SmPC attached.

TR001 Cell Therapy

Section 5.2 of the Investigator's Brochure.

© Copyright: The University of Oxford and Oxford University Hospitals NHS Foundation Trust 2016

Tacrolimus

Summary of Product Characteristics: Envarsus 0.75mg, 1mg and 4mg prolonged-release tablets, manufactured by Chiesi Ltd, Section 4.8 of SmPC attached, or Adoport 0.5mg, 0.75mg, 1mg, 2mg and 5mg hard capsules, manufactured by Sandoz Limited, Section 4.8 of SmPC attached.

Mycophenolate Mofetil

Summary of Product Characteristics: Cellcept manufactured by Roche, Section 4.8 of SmPC attached.

Methylprednisolone

Summary of Product Characteristics: Intravenous injection or tablets, Section 4.8 of SmPC attached.

Paracetamol

Summary of Product Characteristics: 500mg capsules or tablets, Section 4.8 of SmPC attached.

Chlorpheniramine

Summary of Product Characteristics: 4m tablets, Section 4.8 of SmPC attached.

17.6. SUSAR Reporting

All SUSARs will be reported by SITU to the relevant Competent Authority (MHRA) and to the REC and other parties as applicable. For fatal and life-threatening SUSARS, this will be done no later than 7 calendar days after the Sponsor or delegate is first aware of the reaction. Any additional relevant information will be reported within 8 calendar days of the initial report. All other SUSARs will be reported within 15 calendar days.

Principal Investigators will be informed of all SUSARs for the relevant IMP for all studies with the same Sponsor, whether or not the event occurred in the current trial.

17.7. Data and Safety Monitoring Committee

Membership of the Data and Safety Monitoring Committee (DSMC) is given at the start of this protocol; the panel being chosen from those leading in the field of clinical transplantation and/or with experience of previous cell therapy trials in the ONE Study consortium. They will conduct a review of all SAEs as line listings for the trial reported during the previous 6 months and cumulatively. They will also be informed of any SARs or SUSARs as they occur by e-mail notification to the DSMC members. The aims of this committee include:

- To pick up any trends, such as increases in un/expected events, and take appropriate action
- To seek additional advice or information from investigators where required
- To evaluate the risk of the trial continuing and take appropriate action where necessary

© Copyright: The University of Oxford and Oxford University Hospitals NHS Foundation Trust 2016

17.8. Development Safety Update Reports

SITU, on behalf of the CI, will submit (in addition to the expedited reporting above) DSURs once a year throughout the clinical trial, or on request, to the Competent Authority (MHRA in the UK), Ethics Committee, HRA (where required), Host NHS Trust and Sponsor.

18. STATISTICS

18.1. Description of Statistical Methods

Full details of the statistical analysis will be detailed in a separate statistical analysis plan (SAP) which will be drafted early in the trial and finalised prior to the primary analysis data lock. Stata (StataCorp LP) or other appropriate validated statistical software will be used for analysis. A summary of the planned statistical analysis is included here.

Two analysis sets will be defined:

- Intention to-treat population: all patients who signed informed consent and were transplanted will be analysed in the groups to which they were randomised
- Per-protocol population: all patients who signed informed consent, were transplanted and were treated according to protocol specifications. Full details of exclusions from this population will be detailed in the SAP.
- All donors who signed informed consent will provide baseline data to help interpret the immune monitoring data in the control and treatment arms of the recipient participants.

Descriptive statistics will be used to describe the demographics between the treatment groups. Withdrawn patients will also be described fully. Comparative analysis will be undertaken to provide an indication as to whether a definitive phase 3 randomised trial would be appropriate.

For continuous variables, the difference in the means and the corresponding 80% confidence interval will be reported for each treatment group and overall. For continuous variables, t-tests unadjusted or multivariable linear models adjusted for important factors will be applied if normally distributed to compare the intervention and control group. If not normally distributed, non-parametric techniques will be used.

For categorical variables, the number (and percentage) of patients in each category will be reported for each treatment group and overall. For categorical variables, chi-squared tests will be used for comparing treatment groups or multivariable logistic models adjusted for important factors if the variables are normally distributed.

The primary outcome is biopsy proven acute rejection episode and the time to first biopsy proven acute rejection will be analysed using survival analysis techniques. Kaplan-Meier survival curves will be presented graphically. Cox proportional Hazards models will be used both unadjusted and adjusted for important factors. The log-rank test will be used to identify significance. Acute rejection rates at 18 months will be reported for both groups and as a difference in proportions, alongside the hazard ratios and 80% confidence interval will be reported. Patients who have been withdrawn or lost-to follow-up will be censored at their last known rejection-free time. Analysis adjusting for competing risks of allograft failure or death will be considered.

© Copyright: The University of Oxford and Oxford University Hospitals NHS Foundation Trust 2016

18.2. The Number of Participants

As a Phase IIb study this is principally aimed at proving the feasibility and efficacy of Treg therapy to facilitate immunosuppression minimisation to tacrolimus monotherapy and to determine progression to a Phase III trial. The control arm will generate contemporaneous data regarding rejection rates on tacrolimus/MMF and facilitate interpretation of the immune monitoring data from patients receiving cellular therapy. Therefore 34 patients will be recruited into each arm of the study, and based on the 18-month rejection rate amongst patients receiving alemtuzumab followed by tacrolimus/MMF dual therapy in the 3C study being 11% (unpublished, 3C study group), a rejection rate between 6 and 18 months of ≤12% (4/34) will be regarded as successful, thereby supporting further investigation in a Phase III trial. Data from up to 68 kidney donors recruited to the study will inform the immune monitoring analyses and in particular provide information on donor-derived antibody development and Treg/effector T cell antigen-specific responses through use of isolated antigen-presenting cells in *in vitro* assays. In line with section 15 of the protocol, participants who are unable to receive their allocated intervention may be replaced.

18.3. The Level of Statistical Significance

This early phase study has used 20% statistical significance. 80% confidence intervals will be reported.

18.4. Criteria for the Termination of the Trial

The TWO Study Treg therapy trial may be terminated prematurely. The sponsor and CI reserve the right to stop the trial at any time, for any justifiable reason.

In the event of premature discontinuation, SITU will promptly notify the CI, the Sponsor, the responsible regulatory authorities (MHRA). A detailed written explanation of the reasons for early termination will be provided. The affected trial participants will also be informed promptly and appropriate follow-up will be arranged. The CI will also inform the REC.

The clinical trial may be prematurely terminated in the following circumstances:

- A recommendation from the DSMC or TSC
- A request from the regulatory authority
- Failure to meet patient recruitment targets
- Serious and/or persistent non-compliance with the trial protocol
- Inadequate cooperation of the trial centre with the sponsor or the sponsor's representatives
- When the sponsor/CI is aware of important new information that adversely effects the conduct of the study
- Findings uncovered during monitoring visits, trial audits or inspections that compromise the suitability of the site to act as a trial centre

A DSMC assessment will take place after the recruitment of 6 patients into either arm or 6 months from the start of recruitment, whichever is sooner. Subsequent meetings will be held at each sub-Milestone as defined by the funder (annually in February).

A DSMC meeting will also be convened to consider termination of the trial if all of the following criteria are met:

© Copyright: The University of Oxford and Oxford University Hospitals NHS Foundation Trust 2016

- 4 biopsy-proven acute rejection episodes in 4 separate patients
- All 4 patients are in the same arm of the study
- Episodes occur within the first 15 patients recruited to that arm
- Episodes occur between 6 and 18 months post-transplant as per primary endpoint

Furthermore, if the late rejection rate, defined as occurring after 6 months post-transplant, in the Treg treatment arm is greater than 26% at any point during follow-up then the DSMC will meet to consider termination.

Recruitment rates will be continually reviewed throughout the trial by the DSMC and TSC. Poor recruitment rates will have a significant impact on the feasibility of the trial, and factors to mitigate this will be reviewed pro-actively as required. Continued funding by the awarding body (MRC) is also contingent on achieving recruitment target milestones, therefore recruitment of <80% for any given milestone will trigger a formal assessment by the TSC and DSMC. Key target milestones (time from ethical and regulatory approval) are given in table 6.

Table 6. Recruitment targets by time from study initiation

Milestone	Time (from initiation)	Study Arm (number of patients recruited)		No. of patients received cell product
		Standard Care	Cell Therapy	
1	24 months	6	6	6
2	36 months	18	18	18
3	48 months	34	34	34

18.5. Procedure for Accounting for Missing, Unused, and Spurious Data.

Missing data will be minimised by careful data management and staff training. Missing data will be described with reasons given where available; the number and percentage of individuals in the missing category will be presented by treatment arm. The nature and mechanism for missing variables and outcome will be investigated, and if appropriate multiple imputations will be used. Any imputation methods will be fully described in the SAP.

Primary, secondary and exploratory outcomes will be analysed as both per protocol and by intention to treat.

18.6. Procedures for Reporting any Deviation(s) from the Original Statistical Plan

All deviations from the original statistical analysis plan will be provided in the final clinical study report/publication, after discussion with the CI and notification of the Sponsor.

It is foreseeable that this may occur if the trial is terminated prematurely and/or an inadequate number of participants are recruited. In this context an exploratory or descriptive analysis may be deemed more appropriate.

© Copyright: The University of Oxford and Oxford University Hospitals NHS Foundation Trust 2016

19. DATA MANAGEMENT

19.1. Source Data

Source documents are where data are first recorded, and from which participants' electronic database data are obtained. These include, but are not limited to, paper and electronic hospital records (from which medical history and previous and concurrent medication may be summarised into the electronic database), clinical and office charts, laboratory and pharmacy records, diaries, radiographs, and correspondence.

Paper Case Report Forms (CRFs) will be considered source data if the CRF is the site of the original recording (e.g. there is no other written or electronic record of data). All documents will be stored safely in confidential conditions. On all trial-specific documents, other than the signed consent, the participant will be referred to by the trial participant number/code and date of birth, not by name.

19.2. Access to Data

Direct access will be granted to authorised investigators and trial personnel, together with authorised representatives from the Sponsor, host institution and the regulatory authorities to permit trial-related monitoring, audits and inspections.

19.3. Data Recording and Record Keeping

All study data, including some pre-defined IM assays (Gene expression, leucocyte profiling, T cell function & Treg frequency) and safety reporting data, will be entered on to the electronic database (TRI@L-IT web-based platform, programmed by KOEHLER eClinical (Freiburg, Germany; vitessa v7.5.4). This has been validated according to ICH GCP (Section 5.5) requirements, and Standard Operating Procedures shall be maintained.

The participants will be identified by a unique trial specific number, date of birth and/or code in any database. The name and any other identifying detail will not be included in any trial data electronic file. The trial database and data files will be stored on a password protected platform. All trial data will be stored in line with the Medicines for Human Use (Clinical Trials) Amended Regulations 2006 and the Data Protection Act 1998. Initial data will be collected on a paper CRF and then transferred to the eCRF.

The electronic study database will be archived for 30 years by the University of Oxford. At the end of the trial KOEHLER eClinical will provide the sponsor delegate and PIs with an encrypted CD/DVD-ROM containing the completed electronic database which will be backed up and stored securely by the University of Oxford.

Traceability documentation for the infused ATIMP will be stored for a minimum of 30 years by the GSTT GMP unit, OCTRU, the NDS TRIG laboratory and OUH Transplant Unit as per European Commission guidelines ENTR/F/2/SF/dn D(2009) 35810

An immune monitoring database using Microsoft Excel will be held by the University of Oxford in secure, backed up and encrypted servers. This database will hold data from the immune monitoring assays in an anonymised format and data from this database will not be used to guide clinical treatment. Key pre-determined data related to the experimental outcomes (Gene expression, leucocyte profiling, T cell

© Copyright: The University of Oxford and Oxford University Hospitals NHS Foundation Trust 2016

function & Treg frequency) will be uploaded from this database to the eCRF held by Koehler. This database will be backed up via the University's HFS system, stored on magnetic tape in an automated robotic tape library situated in a climate-controlled secure location. Three copies of data are made to three separate tapes with one copy held in the automated tape library and two copies stored in separately located fire-proof safes.

To enable peer review and/or audits from health authorities, all essential source and study documentation (including quality data and logs) must be securely archived after study completion, in accordance with current regulatory requirements (minimum 30 years). The CI and the trial centre should take adequate measures to prevent accidental or premature destruction of these documents.

The CI will act as custodian for the trial data.

20. QUALITY ASSURANCE PROCEDURES

The trial will be conducted in accordance with the current approved protocol, GCP guidance, relevant regulations and standard operating procedures.

20.1. Monitoring

Monitoring will be performed according to GCP by the University of Oxford's Clinical Trials and Research Governance team. Data will be evaluated for compliance with the protocol and accuracy in relation to source documents. Following written standard operating procedures, the monitors will verify that the clinical trial is conducted and data are generated, documented and reported in compliance with the protocol, GCP and the applicable regulatory requirements. In addition to the site monitoring conducted by CTRG, a central monitoring and audit plan will be implemented according to SITU and OCTRU SOPs.

20.2. Data & Safety Monitoring Committee (DSMC)

The DSMC will meet regularly throughout the trial at time-points related to the grant milestones as defined above. The DSMC will review the safety data generated, including all adverse events, and make recommendations as to whether the protocol should be amended to protect patient safety. Recommendations of the DSMC will be discussed between the PIs, TSC, and the Sponsor.

20.3. Trial Steering Committee (TSC)

The role of the Trial Steering Committee (TSC) is to provide the overall supervision of the trial. The TSC will monitor trial progress and conduct, and will advise on scientific credibility. The TSC will consider and act, as appropriate, upon the recommendations of the Data and Safety Monitoring Committee (DSMC) and ultimately carries the responsibility for deciding whether the trial needs to be stopped on grounds of safety or efficacy. Membership of the TSC is given at the start of this protocol.

20.4. Trial Management Group (TMG)

The Trial Management Group (TMG) consists of those individuals responsible for the operational management of the trial such as the lead investigators (CI, PI and assistant investigators), key members of the scientific and clinical team (scientists, clinicians, and research nurses), the clinical

© Copyright: The University of Oxford and Oxford University Hospitals NHS Foundation Trust 2016

trial coordinator, and representatives from OCTRU (clinical trial managers, statisticians, quality assurance managers and regulatory advisers).

The TMG will meet every 1-2 months throughout the lifetime of the Phase 2 study and will:

- Supervise the conduct and progress of the study, and adherence to the study protocol
- Assess the safety as compiled by SITU and assessed by the DSMC, and efficacy of the interventions during the study
- Evaluate the quality of the study data
- Review relevant information from other sources (e.g. related studies)
- Escalate any issues for concern to SITU, specifically where the issue could compromise patient safety or the integrity of the study or quality of the study data

20.5. Project Management Group (PMG)

The Project Management Group (PMG) will have responsibility for reporting to the funding body as per the funder's reporting timetable. Within this remit, the PMG will have oversight for and manage all aspects of finance, staffing, contracts, intellectual property, and regulatory compliance associated with the trial. The PMG will meet regularly throughout the lifetime of the trial.

20.6. Serious Breaches

The Medicines for Human Use (Clinical Trials) Regulations contain a requirement for the notification of "serious breaches" to the MHRA within 7 days of the Sponsor becoming aware of the breach.

A serious breach is defined as "A breach of GCP or the trial protocol which is likely to affect to a significant degree –

(a) the safety or physical or mental integrity of the subjects of the trial; or

(b) the scientific value of the trial".

In the event that a serious breach is suspected the Sponsor must be contacted within 1 working day. In collaboration with the CI, the serious breach will be reviewed by the Sponsor and, if appropriate, the Sponsor will report it to the REC committee, Regulatory authority and the NHS host organisation within seven calendar days.

21. ETHICAL AND REGULATORY CONSIDERATIONS

21.1. Declaration of Helsinki

The Investigator will ensure that this trial is conducted in accordance with the principles of the Declaration of Helsinki (2008), and the Declaration of Istanbul (2008).

21.2. Guidelines for Good Clinical Practice

The Investigator will ensure that this trial is conducted in accordance with relevant regulations and with Good Clinical Practice.

© Copyright: The University of Oxford and Oxford University Hospitals NHS Foundation Trust 2016

21.3. Approvals

The protocol, informed consent form and participant information sheet will be submitted as required to an appropriate Research Ethics Committee (REC), HRA (where required), regulatory authorities (MHRA in the UK), and host institution(s) for written approval.

The Investigator will submit and, where necessary, obtain approval from the above parties for all substantial amendments to the original approved documents.

21.4. Reporting

The CI shall submit once a year throughout the clinical trial, or on request, an Annual Progress Report to the REC, HRA (where required), host organisation and Sponsor. In addition, an End of Trial notification and final report will be submitted to the MHRA, the REC, host organisation and Sponsor.

21.5. Participant Confidentiality

The trial staff will ensure that the participants' anonymity is maintained. The participants will be identified only by a participant ID number +/- date of birth on all trial documents and any electronic database. All documents will be stored securely and only accessible by trial staff and authorised personnel. The trial will comply with the General Data Protection Regulation (GDPR) and Data Protection Act 2018, which requires data to be anonymised as soon as it is practical to do so..

To comply with HTA requirements for ATIMP cell production, patients who donate whole blood for Treg manufacture will have their blood labelled with name, date of birth, NHS number and unique study code identifier prior to shipping, and a copy of their signed consent form together with virology/serology results will be sent securely and separately to the GMP facility. A sample of blood may be retained by the GMP unit for future analysis and traceability. Any such storage will be under an appropriate HTA licence.

21.6. Expenses and Benefits

Reasonable travel expenses for any visits additional to normal care will be reimbursed on production of receipts, or a mileage allowance provided as appropriate.

21.7. Other Ethical Considerations

Potential considerations in this trial are the possibility of a delay to the transplantation surgical procedure due to anaemia resulting from procurement of blood for ATIMP manufacture. However, such a delay has not been observed in our previous Phase I trial in 12 patients. A second procurement of blood due to failed manufacture may also result in anaemia and a subsequent delay to surgery, although in this instance the risk will be explained to the patient who is free to decline a second procurement, withdraw from the trial and undergo transplantation according to standard clinical care without Treg therapy.

No other ethical considerations have been identified in relation to this trial.

22. FINANCE AND INSURANCE

© Copyright: The University of Oxford and Oxford University Hospitals NHS Foundation Trust 2016

22.1. Funding

The TWO Study Treg Cell Therapy Trial is an investigator-initiated, non-commercial clinical trial funded by a Medical Research Council (UK) Biomedical Catalyst: Developmental Pathway Funding Scheme award.

22.2. Insurance

The University has a specialist insurance policy in place which would operate in the event of any participant suffering harm as a result of their involvement in the research (Newline Underwriting Management Ltd, at Lloyd's of London). NHS indemnity operates in respect of the clinical treatment that is provided.

23. PUBLICATION POLICY

All information, data and results obtained from the TWO Study are confidential. Agreement from the Sponsor and TSC will be required prior to the public disclosure of any study-related data.

The results from the TWO Study will be published in peer-reviewed scientific/medical journals and presented at scientific/clinical symposia and congresses.

© Copyright: The University of Oxford and Oxford University Hospitals NHS Foundation Trust 2016

24. REFERENCES

Al-Awwa, I., Hariharan, S., & First, M. (1998). Importance of allograft biopsy in renal transplant recipients: correlation between clinical and histological diagnosis. *Am J Kidney Dis*, 31 (6 suppl 1):S15 - 18.

Ashton-Chess, J., Giral, M., Soulillou, J., & Brouard, S. (2009). Can immune monitoring help to minimize immunosuppression in kidney transplantation? *Transpl Int*, 22(1):110 - 119.

Bacchetta, R., Passerini, L., Gambineri, E., Dai, M., Allan, S., Perroni, L., . . . Roncarolo, M. (2006). Defective regulatory and effector T cell functions in patients with FOXP3 mutations. *J Clin Invest*, 116(6):1713 - 22.

Baker, R., Jardine, A., & Andrews, P. (2011). Renal Association clinical practice guideline on post-operative care of the kidney transplant recipient. *Nephron Clin Pract*, 118 (s1): c311-47.

Barth, R., Janus, C., Lillesand, C., Radke, N., Pirsch, J., Becker, B., . . . Knechtle, S. (2006). Outcomes at 3 years of a prospective pilot study of Campath-1H and sirolimus immunosuppression for renal transplantion. *Transpl Int*, 19(11):885 - 92.

Battaglia, M., Stabilini, A., Migliavacca, B., Horejs-Hoeck, J., Kaupper, T., & Roncarolo, M. (2006). Rapamycin promotes expansion of functional CD4+CD25+FOXP3+ regulatory T cells of both healthy subjects and type 1 diabetic patients. *J Immunol*, 177(12):8338 - 47.

Bennett, C., Christie, J., Ramsdell, F., Brunkow, M., Ferguson, P., Whitesell, L., . . . Ochs, H. (2001). The immune dysregulation, polyendocrinopathy, enteropathy, X-linked syndrome (IPEX) is caused by mutations of FOXP3. *Nat Genet*, 27(1):20 - 1.

Bloom, D., Chang, Z., Fechner, J., Dar, W., Polster, S., Pascual, J., . . . Knechtle, S. (2008). CD4+CD25+FOXP3+ regulatory T cells increase de novo in kidney transplant patients after immunodepletion with Campath-1H. *Am J Transplant*, 8(4):793 - 802.

Brunstein, C., Miller, J., Cao, Q., McKenna, D., Hippen, K., Curtsinger, J., . . . Wagner, J. (2011). Infusion of ex vivo expanded T regulatory cells in adults transplanted with umbilical cord blood: safety profile and detection kinetics. *Blood*, 117(3):1061 - 70.

Burkhalter, F., Oettl, T., Descoeudres, B., Bachmann, A., Guerke, L., Mihatsch, M., . . . Steiger, J. (2012). High incidence of rejection episodes and poor tolerance of sirolimus in a protocol with early steroid withdrawal and calcineurin inhibitor-free maintenance therapy in renal transplantation: experiences of a randomised prospective single-center study. *Transplant Proc*, 44: 2961-65.

Bushell, A., van der Net, J., Game, D., Hilton, R., Thirkell, S., Hester, J., . . . Lombardi, G. (2016). The UK ONE study trial: safety and feasibility of regulatory T (Treg) cell therapy in renal transplantation. *Transplantation*, 100(7S): S120.

Calne, R. (2009). Transplantation: current developments and future directions. *Rev Neurosci*, 20(3-4):267 - 73.

Calne, R., Friend, P., Moffatt, S., A, B., Hale, G., Firth, J., . . . Waldmann, H. (1998). Prope tolerance, perioperative campath 1H, and low-dose cyclosporin monotherapy in renal allograft recipients. *Lancet*, 351: 1701 - 2.

© Copyright: The University of Oxford and Oxford University Hospitals NHS Foundation Trust 2016

Chan, K., Taube, D., Roufosse, C., Cook, T., Brookes, P., Goodall, D., . . . McLean, A. (2011). Kidney transplantation with minimised maintenance: alemtuzumab induction with tacrolimus monotherapy - an open label, randomised trial. *Transplantation* , 92: 774-80.

Chandran, S., Tang, Q., Sarwal, M., Laszik, Z., Putnam, A., Sigdel, T., . . . Vincenti, F. (2016). Polyclonal Treg adoptive therapy for control of subclinical kidney transplant inflammation (TASK pilot trial). *Am J Transplant*, 16(suppl 3): 348.

Cippa, P., & Fehr, T. (2011). Spontaneous tolerance in kidney transplantation - an instructive, but very rare paradigm. *Transpl Int*, 24(6):534 - 535.

de Mattos, A., Olyaei, A., & Bennett, W. (2000). Nephrotoxicity of immunosuppressive drugs: long-term consequences and challenges for the future. *Am J Kidney Dis*, 35(2):333 - 46.

Di Ianni, M., Falzetti, F., Carotti, A., Terenzi, A., Castellino, F., Bonifacio, E., . . . Velardi, A. (2011). Tregs prevent GVHD and promote immune reconstitution in HLA-haploidentical transplantation. *Blood*, 117(14):3921 - 8.

Ekberg, H., & group, f. t.-S. (2007). Reduced exposure to calcineurin inhibitors in renal transplantation. *N Eng J Med*, 357:2562 - 2575.

Ekberg, H., Bernasconi, C., Tedesco-Silva, H., Vitko, S., Hugo, C., Demirbas, A., . . . Halloran, P. (2009). Calcineurin inhibitor minimisation in the Symphony study: observational results 3 years after transplantation. *Am J Transplant*, 9(8): 1876 - 85.

Fleiner, F., Fritsche, L., Glander, P., Neumayer, H., & Budde, K. (2006). Reporting of rejection after renal transplantation in large immunosuppressive trials: biopsy-proven, clinical, presumed, or treated rejection? *Transplantation*, 81(5):655 - 59.

Fontenot, J., Gavin, M., & Rudensky, A. (2003). Foxp3 programs the development and function of CD4+CD25+ regulatory T cells. *Nat Immunol*, 4(4):330 - 6.

Game, D., Hernandez-Fuentes, M., Chaudhry, A., & Lechler, R. (2003). CD4+CD25+ regulatory T cells do not significantly contribute to direct pathway hyporesponsiveness in stable renal transplant patients. *J Am Soc Nephrol*, 14(6):1652 - 61.

Golshayan, D., & Pascual, M. (2008). Minimisation of calcineurin inhibitors to improve long-term outcomes in kidney transplantation. *Transpl Immunol*, 20(1-2):21 - 28.

Golshayan, D., Jiang, S., Tsang, J., Garin, M., Mottet, C., & Lechler, R. (2007). In vitro-expanded donor alloantigen-specific CD4+CD25+ regulatory T cells promote experimental transplantation tolerance. *Blood*, 109(2):827 - 35.

Group, K. W. (2009). KDIGO Clinical practice guideline for the care of kidney transplant recipients. *Am J Transplant*, 9 (Suppl3):S1 - 155.

Group, T. 3. (2013). Campath, calcineurin inhibitor reduction and chronic allograft nephropathy (3c) study: background, rationale, and study protocol. *Transplant Res*, 2:7.

© Copyright: The University of Oxford and Oxford University Hospitals NHS Foundation Trust 2016

Group, T. 3. (2014). Alemtuzumab-based induction treatment versus basiliximab-based induction treatment in kidney transplantation (the 3C study): a randomised trial. *Lancet*, 384: 1684 - 1690.

Gutierrez-Dalmau, A., & Campistol, J. (2007). Immunosuppressive therapy and malignancy in organ transplant recipients: a systematic review. *Drugs*, 67(8):1167 - 98.

Haas, M., Sis, B., Racusen, L., Solez, K., Glotz, D., Colvin, R., . . . committee, B. m. (2014). Banff 2013 meeting report: inclusion of c4d-negative antibody-mediated rejection and antibody-associated arterial lesions. *Am J Transplant*, 14(2):272 - 83.

Halloran, P. (2004). Immunosuppressive drugs for kidney transplantation. *J Engl J Med*, 351(26): 2715 - 29.

Hester, J., Schiopu, A., Nadig, S., & Wood, K. (2012). Low-dose rapamycin treatment increases the ability of human regulatory T cells to inhibit transplant arteriosclerosis in vivo. *Am J Transplant*, 12(8):2008 - 16.

Hori, S., Nomura, T., & Sakaguchi, S. (2003). Control of regulatory T cell development by the transcription factor FOXP3. *Science*, 299(5609):1057 - 61.

Issa, F., Hester, J., Goto, R., Nadig, S., Goodacre, T., & Wood, K. (2010). Ex vivo-expanded human regulatory T cells prevent the rejection of skin allografts in a humanized mouse model. *Transplantation*, 90(12):1321 - 7.

Jovanovic, V., Lair, D., Soulillou, J., & Brouard, S. (2008). Transfer of tolerance to heart and kidney allografts in the rat model. *Transpl Int*, 21(3):199 - 206.

Karuthu, S., & Blumberg, E. (2012). Common infections in kidney transplant recipients. *Clin J Am Soc Nephrol*, 7 (12):2058 - 70.

Kasiske, B., Guijarro, C., Massy, Z., Wiederkehr, M., & Ma, J. (1996). Cardiovascular disease after renal transplant. *J Am Soc Nephrol*, 7(1):158 - 65.

Kendrick, E. (2001). Cardiovascular disease and the renal transplant recipient. *Am J Kidney Dis*, 38(s6): S36 - 43.

Kingsley, C., Nadig, S., & Wood, K. (2007). Transplantation tolerance: lessons from experimental rodent models. *Transpl Int*, 20(10):828 - 841.

Knechtle, S., Pirsch, J., Fechner, H., Becker, B., Friedl, A., Colvin, R., . . . Sollinger, H. (2003). Campath-1H induction plus rapamycin monotherapy for renal transplantation: results of a pilot study. 3(6): 722 - 30.

LAchenbruch, P., Rosenberg, A., Bonvini, E., Cavaille-Coll, M., & Colvin, R. (2004). Biomarkers and surrogate endpoints in renal transplantation: present status and considerations for clinical trial design. *Am J Transplant*, 4(4):451 - 57.

Lebranchu, Y., Thierry, A., Toupance, O., Westeel, P., Etienne, I., Thervet, E., . . . Hurault de Ligny, B. (2009). Efficacy on renal function of early conversion from cyclosporine to sirolimus 3 months after renal transplantation: Concept Study. *Am J Transplant*, 9: 1115-1123.

© Copyright: The University of Oxford and Oxford University Hospitals NHS Foundation Trust 2016

London, N., Farmery, S., Will, E., Davison, A., & Lodge, J. (1995). Risk of neoplasia in renal transplant patients. *Lancet*, 346(8972):403 - 6.

Marek-Trzonkowska, N., Mysliwiec, M., Dobyszuk, A., Grabowska, M., Derkowska, I., Juscinska, J., . . . Trzonkowski, P. (2014). Therapy of type 1 diabetes with CD4(+)CD25(high)CD127- regulatory T cells prolongs survival of pancreatic islets - results of one year follow-up. *Clin Immunol*, 153(1):23 - 30.

Marek-Trzonkowska, N., Mysliwiec, M., Dobyszuk, A., Grabowska, M., Derkowska, I., Juscinska, J., . . . Trzonkowski, P. (2014). Therapy of type 1 diabetes with CD4(+)CD25(high)CD127-regulatory T cells prolongs survival of pancreatic islets - results of one year follow-up. *Clin Immunol, 153*(1), 23-30.

Markmann, J., Guinan, E., Geissler, E., Cole, ,. G., Germana, S., Kim, J., & Sawitzki, B. (2016). Initial experience with donor specific Tregs in kidney transplantation. *Am J transplant*, 16(suppl 3):271.

Marreiter, R., Klempnauer, J., Neuhaus, P., Muehlbacher, F., Boesmueller, C., & Calne, R. (2008). Alemtuzumab (campath-1H) and tacrolimus monotherapy after renal transplantation: results of a prospective randomized trial. *Am J Transplant*, 8: 1480-85.

Mathis, A., Dave, N., Knipp, G., & Friedman, G. (2004). Drug-related dsylipidaemia after renal transplantation. *Am J Health Syst Pharm*, 61(6): 565 - 85.

Meier-Kriesche, H., Schold, J., Srinivas, T., & Kaplan, B. (2004). Lack of improvement in renal allograft survival despite a marked decrease in acute rejection rates over the most recent era. *Am J Transplant*, 4(3):378 - 83.

Merrill, J., Murray, J., Harrison, J., & Guild, W. (1956). Successful homotransplantation of the human kidney between identical twins. 160 (4): 277-82.

Montero, N., & Pascual, J. (2015). Immunosuppression and post-transplant hyperglycaemia. *Curr Diabetes Rev*, 11(3):144 - 54.

Morris, P. (2004). Transplantation - a medical miracle of the 20th century. *N Engl J Med*, 351 (26): 2678 - 80.

Nadig, S., Wieckiewicz, J., Wu, D., Warnecke, G., Zhang, W., Luo, S., . . . Wood, K. (2010). In vivo prevention of transplant arteriosclerosis by ex vivo-expanded human regulatory T cells. *Nat Med*, 16(7):809 - 13.

Naesens, M., Kuypers, D., & Sarwal, M. (2009). Calcineurin inhibitor toxicity. *Clin J Am Soc Nephrol.*, 4(2): 481 - 508.

Nakivell, B., Borrows, R., Fung, C., O'Connell, P., & Allen, R. C. (2003). The natural history of chonic allograft nephropathy. *N Eng J Med*, 349(24):2326 - 33.

National Institute for Health and Care Excellence. (2004). Immunosuppresive therapy for renal transplantation in adults. *Technology Appraisal Guidance 85*.

© Copyright: The University of Oxford and Oxford University Hospitals NHS Foundation Trust 2016

Opelz, G., Dohler, B., & Report, C. T. (2008). Influence of time of rejection on long-term graft survival in renal transplantation. *Transplantation*, 85(5):661 - 66.

Orlando, G., Hematti, P., Stratta, R., Burke, G. 3., Di Cocco, P., Pisani, F., . . . Wood, K. (2010). Clinical operational tolerance after renal transplantation: current status and future challenges. *Ann Surg*, 252(6):915 - 28.

Pascual, J., Bloom, D., Torrealba, J., Brahmbhatt, R., Chang, Z., Sollinger, H., & Knechtle, S. (2008). Calcineurin inhibitor withdrawal after renal transplantation with alemtuzumab: clinical outcomes and effect on T-regulatory cells. *Am J Transplant*, 8(7):1529 - 36.

Pascual, M., Vallhonrat, H., Cosimi, A., Tolkoff-Rubin, N., Colvin, R., Delmonico, F., . . . Williams, W. J. (1999). The clinical usefulness of the renal allograft biopsy in the cyclosporine era: a prospective study. *Transplantation*, 67(5):737 - 41.

Ponticelli, C., & Scolari, M. (2010). Calcineurin inhibitors in renal transplantation still needed but in reduced doses: a review. *Transplant Proc*, 42(6):2205 - 8.

Qin, S., Cobbold, S., Pope, H., Elliott, J., Kioussis, D., Davies, J., & Waldmann, H. (1993). "Infectious" transplantation tolerance. *Science*, 259(5097):974 - 7.

Rama, I., & Grinyo, J. (2010). Malignancy after renal transplantation: the role of immunosuppression. *Nat Rev Nephrol*, 6 (9):511 - 19.

Roussey-Kesler, G., Giral, M., Moreau, A. S., Legendre, C., Noel, C., Pillebout, E., . . . Soulillou, J. (2006). Clinical operational tolerance after kidney transplantation. *Am J Transplant*, 6(4):736 - 46.

Sayegh, M., & Carpenter, C. (2004). Transplantation 50 years later - progress, challenges, and promises. *N Eng J Med*, 351 (26): 2761 - 66.

Schena, F., Pascoe, M., Alberu, J., del Carmen Rial, M., Oberbauer, R., Brennan, D., . . . Group, f. t. (2009). Conversion from calcineurin inhibitors to sirolimus maintenance therapy in renal allograft recipients: 24-month efficact and safety results from the CONVERT Trial. *Transplantation*.

Skaro, A. L., Mathew, J., Gallon, L., Hie, J., Hansen, C., Stare, D., . . . Leventhal, J. (2016). Results of a phase 1 trial of Treg adoptice cell transfer (TRACT) in de novo living donor kidney transplant recipients. *Am J Transplant*, 16(suppl3): 271.

Sutherland, A., Akhtar, M., Zilvetti, M., Brockmann, J., Ruse, S., Fuggle, S., . . . Friend, P. (2014). Alemtuzumab and sirolimus in renal transplantation: six-year results of a single-arm prospective pilot study. *Am J Transplant*, 14(3):677 - 84.

Tan, H., Donaldson, J., Basu, A., Unruh, M., Randhawa, P., Sharma, V., . . . Shapiro, R. (2009). Two hundred living donor kidney transplantations under alemtuzumab induction and tacrolimus monotherapy: 3-year follow-up. *Am J Transplant*, 9: 355-66.

Tronkowski, P., Zilvetti, M., Chapman, S., Wieckiewicz, J., Sutherland, A., Friend, P., & Wood, K. (2008). Homeostatic repopulation by CD28-CD8+ T cells in alemtuzumab-depleted kidney transplant recipients treated with reduced immunosuppression. *Am J Transplant*, 8(2): 338-47.

© Copyright: The University of Oxford and Oxford University Hospitals NHS Foundation Trust 2016

Trzonkowski, P., Zilvetti, M., Friend, P., & Wood, K. (2006). Recipient memory-like lymphocytes remain unresponsive to graft antigens after CAMPATH-1H induction with reduced maintenance immunosupression. *Transplantation*, 82(10):1342 - 51.

Villanueva, M., Munoz, A., Casasola, C., Africa, R., Danguilan, R., & Ona, E. (2008). Alemtuzumab induction with tacrolimus monotherapy in de novo renal transplantation. *Transplant Proc*, 40: 2222-25.

Weir, M., Mulgaonkar, S., Chan, L., Shidban, H., Waid, T., Preston, D., . . . Pearson, T. (2010). Mycophenolate mofetil-vased immunosuppression with sirolimus in renal transplantation: a randomized, controlled Spare-the-Nephron trial. *Kidney Int*, 79: 897-907.

Welberry Smith, M., Cherukuri, A., Newstead, C., Lewington, A., Ahmad, N., Menon, K., . . . Baker, R. (2013). Alemtuzumab induction in renal transplantation permits safe steroid avoidance with tacrolimus monotherapy: a randomized controlled trial. *Transplantation*, 96: 1082-88.

Welberry Smith, M., Newstead, C., Ahmad, N., Lewington, A., Tibble, S., Lodge, J., . . . Baker, R. (2008). Poor tolerance of sirolimus in a steroid avoidance regimen for renal transplantation. *Transplantation*, 85: 636-639.

Yanik, E., Gustafson, S., & Kasiske, B. (2015). Sirolimus use and cancer incidence among US kidney transplant recipients. *Am J Transplant*, 15:129 - 36.

© Copyright: The University of Oxford and Oxford University Hospitals NHS Foundation Trust 2016

25. APPENDIX A: AMENDMENT HISTORY

<table>
<tr><th>Amendment No.</th><th>Protocol Version No.</th><th>Date issued</th><th>Author(s) of changes</th><th>Details of Changes made</th></tr>
<tr><td>1</td><td>V4.0</td><td>15Aug2018</td><td>M.Brook</td><td>Objectives and Outcomes
Additional information regarding intended analysis of transplant biopsy samples added.
Addition of quality of life to outcomes measures and associated use of SF-36 and EQ-5D-5L.
Change to storage of and access to biopsy samples
Clarification of location of storage
Change to medications
nIMPs detailed elsewhere in protocol now added to synopsis
Clarification that immediate release and modified release formulations acceptable
Safety Reporting & review
Adjustment to reporting procedures for SAEs.
Change of criteria for DSMC review and frequency of DSMC reviews.
Databases
Defined immune monitoring assays to be uploaded to Koehler database.
SAE data to be uploaded onto Koehler database
For full details see separate document kept in eTMF (TWO_ProtocolAmendment1Changes_v1.0_15Aug2018) Available on request from Dr M. Brook (matthew.brook@nds.ox.ac.uk) or any member of the TMG.</td></tr>
<tr><td>2</td><td>v5.0</td><td>18Mar2019</td><td>M Brook
J Colston</td><td>Objectives and Outcomes
Additional exploratory outcome added:
Assessment of viral infections within transplant recipients by next generation sequencing of detected viruses.
Sample handling
Clinically-indicated EDTA viral monitoring samples will be used to perform next generation sequencing where viral illness occurs.
Period in which blood procurement can take place has been changed from 6 weeks pre-transplant to 3 – 6 weeks.</td></tr>
</table>

© Copyright: The University of Oxford and Oxford University Hospitals NHS Foundation Trust 2016

Protocol amendments must be submitted to the Sponsor for approval prior to submission to the REC committee or MHRA.

© Copyright: The University of Oxford and Oxford University Hospitals NHS Foundation Trust 2016

Figure S1

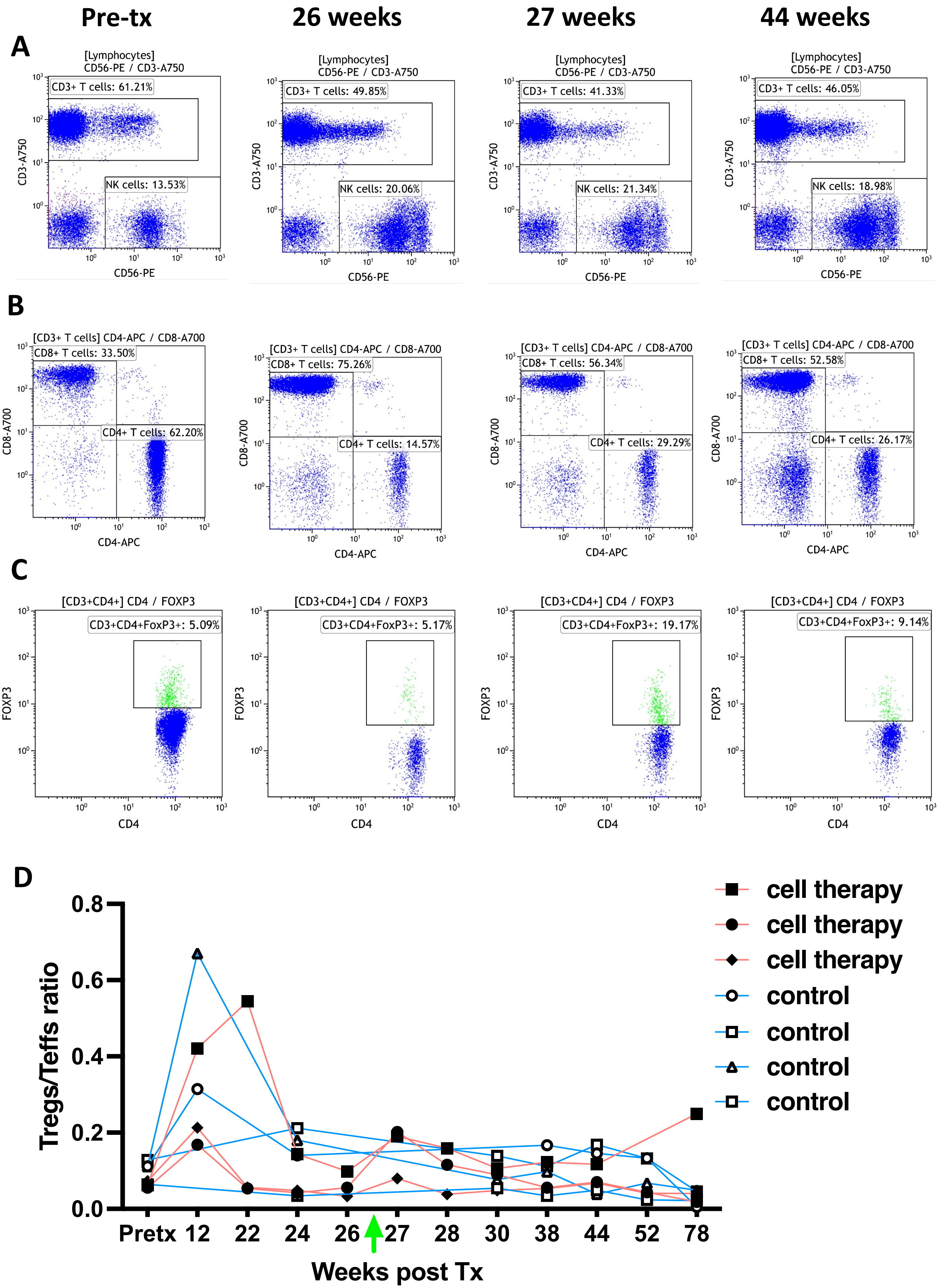
Pre-tx
26 weeks
27 weeks
44 weeks
A
[Lymphocytes] CD56-PE / CD3-A750
CD3+ T cells: 61.21%
NK cells: 13.53%
CD3+ T cells: 49.85%
NK cells: 20.06%
CD3+ T cells: 41.33%
NK cells: 21.34%
CD3+ T cells: 46.05%
NK cells: 18.98%
CD3-A750
CD56-PE
B
[CD3+ T cells] CD4-APC / CD8-A700
CD8+ T cells: 33.50%
CD4+ T cells: 62.20%
CD8+ T cells: 75.26%
CD4+ T cells: 14.57%
CD8+ T cells: 56.34%
CD4+ T cells: 29.29%
CD8+ T cells: 52.58%
CD4+ T cells: 26.17%
CD8-A700
CD4-APC
C
[CD3+CD4+] CD4 / FOXP3
CD3+CD4+FoxP3+: 5.09%
CD3+CD4+FoxP3+: 5.17%
CD3+CD4+FoxP3+: 19.17%
CD3+CD4+FoxP3+: 9.14%
FOXP3
CD4
D
Tregs/Teffs ratio
0.0
0.2
0.4
0.6
0.8
Pretx 12 22 24 26 27 28 30 38 44 52 78
Weeks post Tx
cell therapy
cell therapy
cell therapy
control
control
control
control

Figure S1.

(A-C)) Representative flow cytometry dot plots depicting circulating $CD3^+$ T (A), $CD8^+$ and $CD4^+$ T cells (B) and $FoxP3^+CD4^+$ Tregs (C) assessed before transplantation and at 26, 27 and 44 weeks post transplantation in the peripheral blood of one of the patients receiving cell therapy. **(D)** Ratio of Tregs ($FoxP3^+CD4^+CD3^+$) to Teff ($FoxP3^{neg}CD4^+CD3^+$) cells in the peripheral blood at the indicated time points.

Figure S2

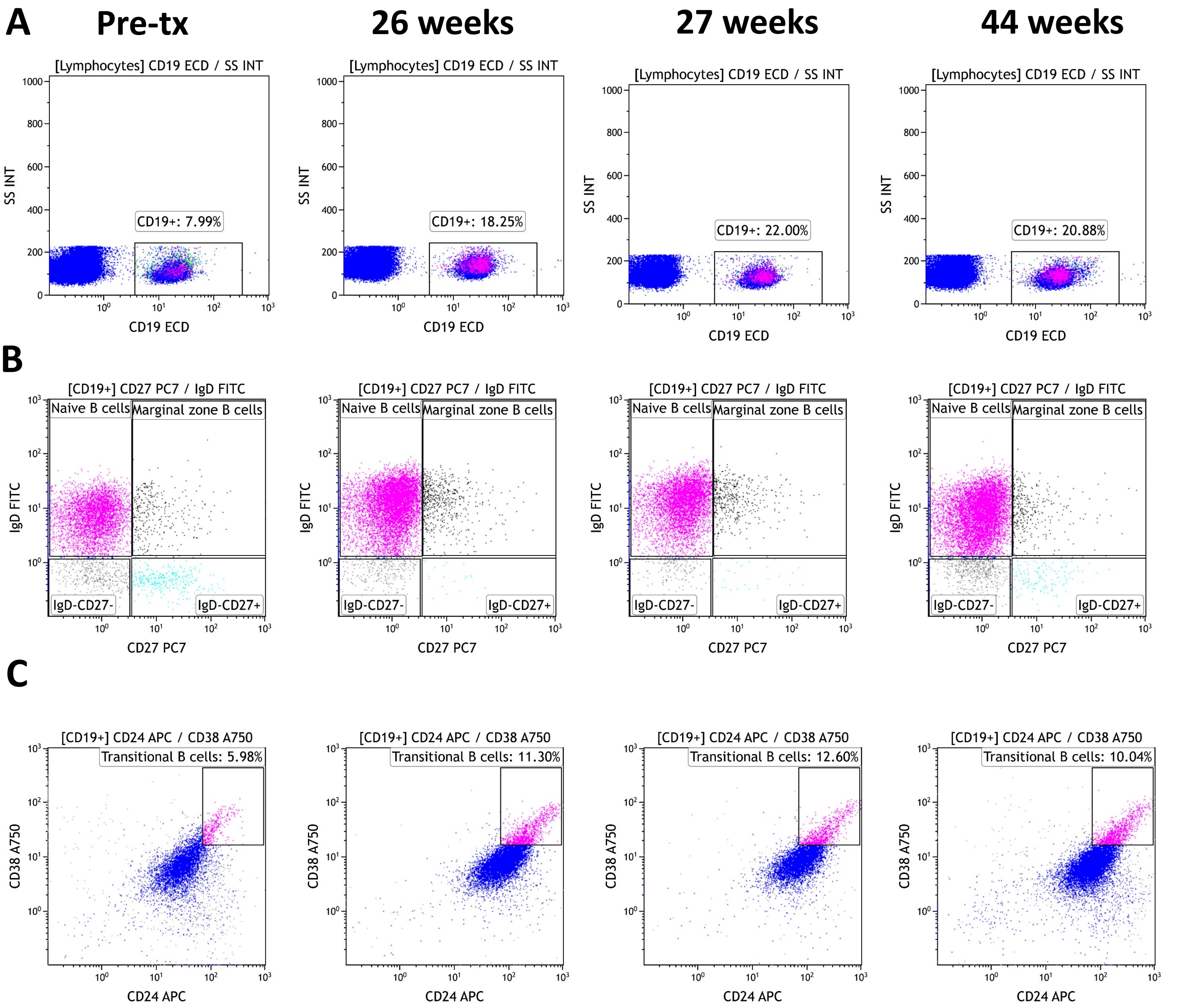

A
Pre-tx
26 weeks
27 weeks
44 weeks
[Lymphocytes] CD19 ECD / SS INT
SS INT
CD19 ECD
CD19+: 7.99%
CD19+: 18.25%
CD19+: 22.00%
CD19+: 20.88%
B
[CD19+] CD27 PC7 / IgD FITC
IgD FITC
CD27 PC7
Naive B cells
Marginal zone B cells
IgD-CD27-
IgD-CD27+
C
[CD19+] CD24 APC / CD38 A750
CD38 A750
CD24 APC
Transitional B cells: 5.98%
Transitional B cells: 11.30%
Transitional B cells: 12.60%
Transitional B cells: 10.04%

Figure S2.

(A-C) Representative flow cytometry dot plots depicting circulating $CD19^{+}$ B cells (A), naïve and memory B cell subpopulations (B) and $CD24^{hi}CD38^{hi}$ transitional B cells (C) assessed before transplantation and at 26, 27 and 44 weeks post transplantation in the blood of one of the patients receiving cell therapy.
